# An automated aquatic rack system for rearing marine invertebrates

**DOI:** 10.1186/s12915-020-00772-w

**Published:** 2020-05-04

**Authors:** Jonathan Q. Henry, Maryna P. Lesoway, Kimberly J. Perry

**Affiliations:** Department of Cell & Developmental Biology, University of Illinois, 601 South Goodwin Ave., Urbana, IL 61801 USA

**Keywords:** Mariculture, Aquaculture, Automation, Automated feeding, Aquarium, *Crepidula*

## Abstract

**Background:**

One hundred years ago, marine organisms were the dominant systems for the study of developmental biology. The challenges in rearing these organisms outside of a marine setting ultimately contributed to a shift towards work on a smaller number of so-called model systems. Those animals are typically non-marine organisms with advantages afforded by short life cycles, high fecundity, and relative ease in laboratory culture. However, a full understanding of biodiversity, evolution, and anthropogenic effects on biological systems requires a broader survey of development in the animal kingdom. To this day, marine organisms remain relatively understudied, particularly the members of the Lophotrochozoa (Spiralia), which include well over one third of the metazoan phyla (such as the annelids, mollusks, flatworms) and exhibit a tremendous diversity of body plans and developmental modes. To facilitate studies of this group, we have previously described the development and culture of one lophotrochozoan representative, the slipper snail *Crepidula atrasolea*, which is easy to rear in recirculating marine aquaria. Lab-based culture and rearing of larger populations of animals remain a general challenge for many marine organisms, particularly for inland laboratories.

**Results:**

Here, we describe the development of an automated marine aquatic rack system for the high-density culture of marine species, which is particularly well suited for rearing filter-feeding animals. Based on existing freshwater recirculating aquatic rack systems, our system is specific to the needs of marine organisms and incorporates robust filtration measures to eliminate wastes, reducing the need for regular water changes. In addition, this system incorporates sensors and associated equipment for automated assessment and adjustment of water quality. An automated feeding system permits precise delivery of liquid food (e.g., phytoplankton) throughout the day, mimicking real-life feeding conditions that contribute to increased growth rates and fecundity.

**Conclusion:**

This automated system makes laboratory culture of marine animals feasible for both large and small research groups, significantly reducing the time, labor, and overall costs needed to rear these organisms.

## Background

Marine organisms account for 50–80% of all life on earth (WoRMS, www.marinespecies.org) and include representatives from nearly all phyla. A number of marine invertebrate species have been used as models to study biological processes in the lab. This includes representatives from all three of the major bilaterian clades: the deuterostomes (e.g., echinoderms, hemichordates, and ascidians), ecdysozoans (e.g., arthropods), and lophotrochozoans (the Spiralia [1], e.g., annelids and mollusks). Also included are more basal animals such as the cnidarian, *Nematostella vectensis* [2]; the sponge, *Amphimedon queenslandica* [3]; and the ctenophore, *Mnemiopsis leidyi* [4]. Collectively, these species serve as useful representatives for each of their respective phyla and permit evolutionary biological comparisons for studies of cell, developmental, and molecular biology. However, challenges remain in maintaining marine organisms outside of laboratories with access to flowing seawater. Additionally, many of these animals have a biphasic life cycle with periods of obligatory planktonic larval development. These conditions limit long-term culture and the development of genetic tools for explorations of gene function, including rearing inbred and transgenic lines. Much has been described about maintaining marine aquaria for the hobbyist [5, 6]; however, in this paper, we describe the development of an automated marine aquatic system optimized for rearing large numbers of marine organisms in the laboratory setting. This system is especially well suited for rearing filter-feeding marine invertebrates, but can be used to culture most other marine species.

The impetus for developing this aquatic system came from our studies using marine slipper snails in the genus *Crepidula*. Different species of *Crepidula* have been used as subjects for developmental studies for over 100 years, beginning with Conklin’s cell lineage work at the Marine Biological Laboratory in Woods Hole, MA [7]. *Crepidula* has contributed greatly to our understanding of many subjects, including development, evolution, larval biology, metamorphosis, invasion biology, sex change/sequential hermaphroditism, population genetics, biomineralization, and responses to climate change [1, 8–10]. We have been developing one species in particular, the black-footed slipper snail *Crepidula atrasolea*, as a useful representative of the Lophotrochozoa [9]. In many respects, this snail presents fewer challenges for experimental manipulation and long-term culture compared to other spiralians.

*C. atrasolea* is a southern, warm-water species with rapid, direct development and relatively short generation times. Like other *Crepidula* species, *C. atrasolea* snails have internal fertilization, and females brood encapsulated embryos, which hatch as “crawl-away” juveniles [11]. These snails mature relatively quickly and can live for several years in the lab. Furthermore, adults are reproductive year-round. Their small size (shells reaching 1–2 cm long in the lab) allows large numbers of animals to be maintained in a fairly limited space. They are easily reared through successive generations in recirculating system aquaria using artificial seawater [9]. Furthermore, these snails are filter feeders, and they readily survive on commercial preparations of phytoplankton (see below). As *C. atrasolea* is a direct developer, there is no need to provide external food sources prior to reaching juvenile stages. Compared to many other systems, the eggs and embryos of *Crepidula* snails offer significant advantages for experimental cell and molecular biology [1, 8–10, 12–14]. Our recent success in applying CRISPR/Cas9 and electroporation of linearized plasmids in *C. atrasolea* offers us the ability to prepare inbred transgenic lines expressing key fluorescently labeled, cell-specific markers and molecular biosensors.

The major challenge for working with any marine organism, including slipper snails, is the time and effort required to maintain and rear enough animals to support an active research program. While some groups are beginning to address issues of standardizing laboratory culture for marine organisms, for example, the annelid *Platynereis dumerilii* [15] and the hydrozoan *Clytia hemispherica* [16], these systems have limited automation and require manual feeding. To address these challenges, we have developed an automated marine aquatic system that monitors and regulates seawater quality and incorporates automated feeding. Our main goal was to develop equipment and conditions to quickly raise and maintain large numbers of inbred and, ultimately, transgenic lines of *C. atrasolea*. We also wanted to develop a system that could be used by other labs working with other marine organisms. To accomplish this, we designed and assembled the following: a self-contained, recirculating system that can be used in any lab (including those without access to natural, flowing seawater); a system with automated monitoring and control of major seawater quality parameters; a system requiring few water changes with efficient management of waste products (i.e., ammonia, nitrites, and nitrates); a system with automated logging and reporting of all system parameters, including the ability to send users alerts related to any problems that may develop; and a system that incorporates automated feeding. This aquatic system can be used by any lab wanting to rear various marine organisms, but is particularly well-suited for filter-feeding marine invertebrates that consume phytoplankton. By reducing the amount of labor needed to rear these animals, we have freed investigators to conduct more of their actual research.

Depending on their size, this aquatic system can be used to rear many types of marine organisms, but with automated feeding of liquid food, it is especially well-suited for rearing filter-feeding animals. *Crepidula* snails are relatively small, and one can rear large numbers, without creating a biological load that exceeds the capacity of the filters. In fact, one can use this system to raise larger animals, as the supplier of parts used to assemble this system (Iwaki Aquatics, Holliston, MA) can provide tanks of various sizes exceeding 30 l. However, animals that require solid food would need to be fed manually, and provisions would also have to be made to remove uneaten food. Automated delivery of solid, commercial, frozen, or live food would entail a more elaborate setup, and we have not yet explored those options. The system described here is not well-suited for animals that undergo mass, free spawning to release large quantities of gametes (such as sea urchins). The level of filtration provided by the system is sufficient to ensure that gametes are unable to cross between tanks; however, such occurrences often trigger widespread spawning in multiple animals, and would likely overload the capacity of the filtration system. Ideally, the user should remove spawning animals or interrupt flow to individual tanks for spawning, returning them to the system once spawning is complete. Unlike sea urchins, *Crepidula* snails have internal fertilization and brood their embryos until hatching. Other marine species that would be amenable to growth in this aquatic system include the annelid *Hydroides*, the ascidian *Ciona intestinalis*, the gastropod *Tritia* (=*Ilyanassa*) *obsoleta*, and possibly the small-bodied cephalopod *Euprymna scolopes*, to name a few. As all of the parameters of the system (e.g., temperature, salinity, flow rate) can be altered to suit the preferences of the species being reared in the system, even the brackish water cnidarian *Nematostella victensis* should be able to be grown in this system.

## Methods/results/discussion

### The concept of aquatic rack systems

**Fig. 1:**
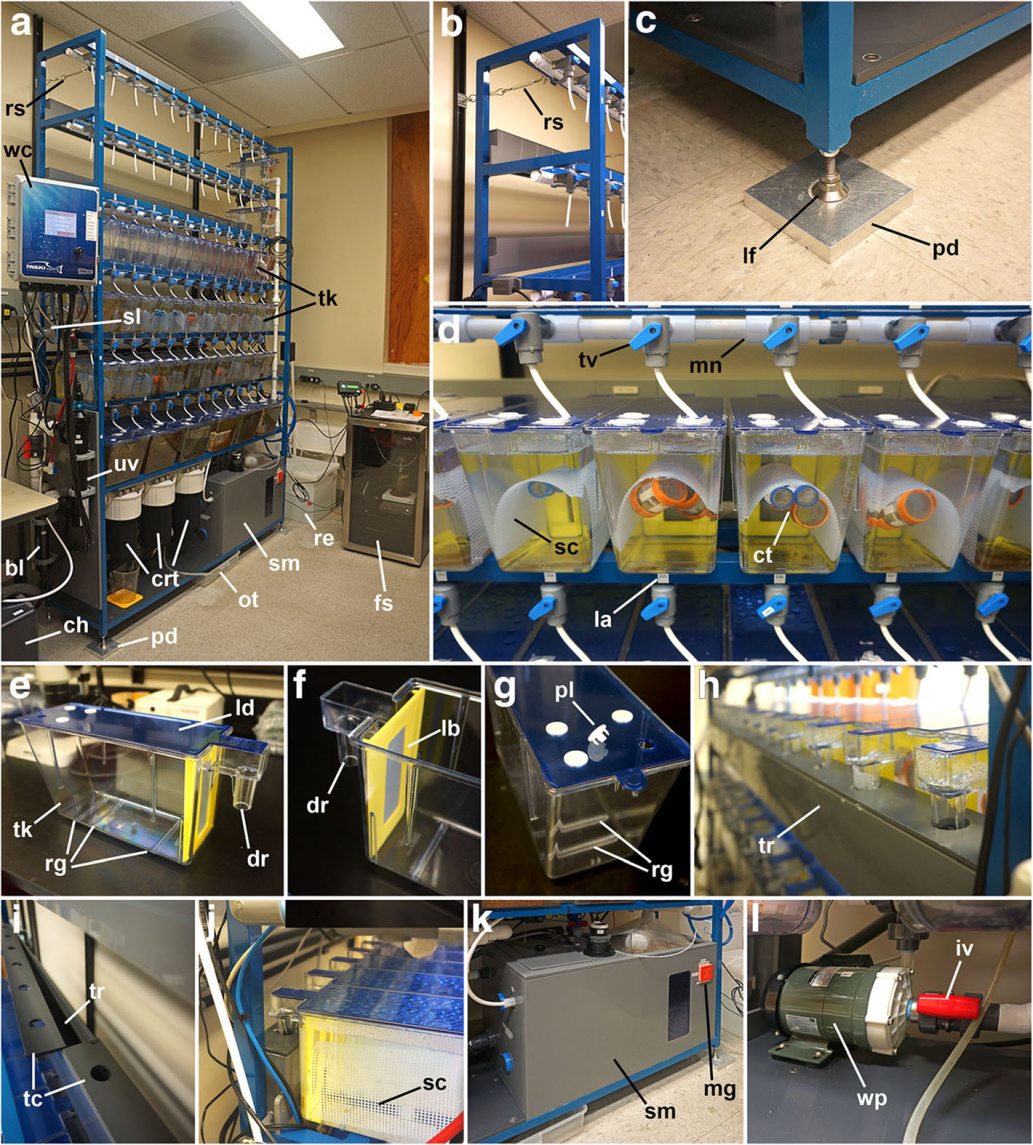
**a**–**l** Overview of the rack system, individual tanks, drain trough, and water pump. **a** Rack system to the left and associated feeding system to the right. Part of the external chiller can be seen on the floor in the bottom, far left corner. **b** Detail of the wall restraints that prevent tipping. **c** Detail of the foot pad to prevent sliding and to distribute weight (see Additional file 3A). **d** Row of 3-l tanks showing the manifold and valves that supply water to these tanks. Some culture tubes are seen inside these tanks, which are held down by the curved plastic mesh. **e** Empty 3-l tank with lid. **f** Detail of the overflow drain and larval baffle. **g** Detail of the plastic plugs used to cap unused holes in the lid. **h** Drain trough that receives the tanks’ overflow drain tubes. **i** Detail of the PVC covers for the drain troughs (see Additional file 3B-C). **j** Another view of the curved plastic restraining screen. **k** Sump. **l** Main water pump. This pump is secured to the bottom PVC deck with four bolts and rubber shock mounts. bl, ballast; ch, chiller; crt, cartridge filters; ct, culture tubes; dr, overflow drain; fs, feeding system; iv, water pump intake valve; la, labels; lb, larval baffles; ld, tank lid; lf, leveling feet; mg, magnet; mn, manifold; ot, overflow tray; pd, foot pad; pl, plugs; re, reservoirs; rg, plastic ridges; rs, rack restraint; sc, plastic restraining screen; sl, sensor side loop; sm, sump; tc, trough covers; tk, tanks; tr, drain trough; tv, tank valve; uv, UV lamp; wc, Walchem 900 controller; wp, water pump

**Fig. 2:**
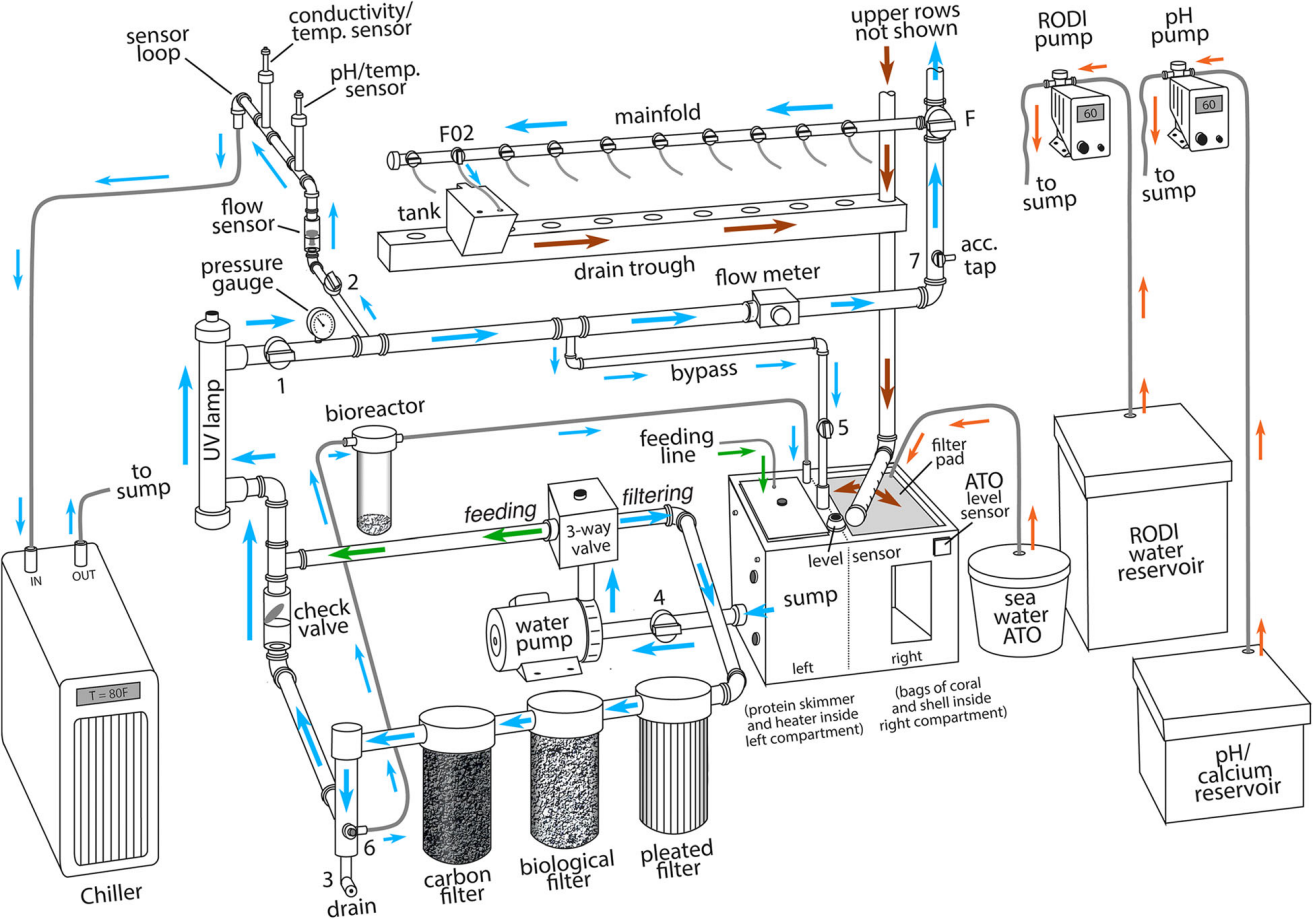
Exploded diagram showing the main components of the aquatic system and fluid flow. Various parts are as labeled. The various valves are also labeled and/or numbered, as referred to in the text and Figure S2 in Additional file 1, SOP. Parts are shown in their relative, approximate locations but are not drawn to scale. Blue arrows show the main routes of the seawater flow. The size of the arrows indicates the relative amount of flow through the various passage ways. The electromechanical 3-way valve can divert seawater either to the right towards the filters during the filtering mode or to the left through the feeding bypass loop during the feeding mode. Green arrows show the delivery of food and flow of seawater through the feeding bypass loop. The one-way check valve prevents seawater containing food from traveling backwards through the filters during the feeding mode. Brown arrows show the return of seawater collected in the drain troughs that travels back to the sump via the drain pipes and slotted diverter. Orange arrows show the flow of seawater, RODI water, and other reagents that are periodically added to the system (sump). The bypass valve #5 can be opened to prevent pump cavitation and to reduce the flow of water to the tanks, as needed. So far, we have not had to use the UV lamp or the second pH/calcium dosing pump (calcium supplements have been added to the system manually). The feeding system and its associated parts (e.g., sensors) are not shown here

Recirculating aquatic rack systems consisting of a parallel array of individual, isolated tanks are widely used for rearing aquatic animals in the laboratory setting. Water in these systems is continuously recirculated between individual tanks and a central sump, passing through various filters along the way (Fig. 1a, Fig. 2). For instance, aquatic rack systems are commonly used to rear freshwater zebrafish and *Xenopus* frogs [17–19] and widely used for commercial aquaculture [20, 21]. These systems typically incorporate mechanical and biological filtration and some percentage of daily water changes to remove accumulated waste products [22]. Such systems work very well for housing large numbers of animals. The presence of many smaller tanks allows one to isolate animals, which is particularly useful for maintaining larger numbers, different species, or different lines (e.g., inbred lines, mutants, or transgenic lines). This configuration also permits controlled mating for breeding purposes. Therefore, we chose to adopt a similar system for rearing marine invertebrates with more specialized features, which are detailed below.

A number of companies currently manufacture aquatic rack systems for freshwater species including Iwaki Aquatics (Holliston, MA), Aquaneering (San Diego, CA), Techniplast (Buguggiate, VA, Italy), and Aquatic Enterprises (Bridgewater, MA). A number of engineers who worked for companies that formerly made marine systems (including Marine Biotech, Aquatic Habitats (a division of Aquatic Ecosystems, Inc.), and Pentair (all based in Apopka, FL)) have since moved to Iwaki Aquatics (Holliston, MA). Given their heritage and level of expertise, we collaborated with them for the final design and fabrication of a marine aquatic rack system. This system is similar to that used for housing zebrafish, but incorporates many additional, custom modifications. Our main goal was to build a system that was highly automated and incorporates automated feeding, in order to reduce labor and make it easier to rear *Crepidula* snails. A companion set of documents, including the Aquatic System Standard Operating Procedure “SOP” and a Parts and Suppliers List, are provided in Additional files 1 and 2, respectively. Additional file 1 includes Figures S1-S4. Many details related to the construction, operation, and servicing of this system are provided in those documents. This includes wiring diagrams (.fzz files, which can be opened with Fritzing Software [23]) and Arduino Sketches (.ino files, which can be opened and installed with Arduino Software (IDE) [24]) for use in programming the microcontrollers. While we developed this aquatic rack system with Iwaki Aquatics, Inc. (Holliston, MA), enough detail is provided so that a skilled builder could assemble one for themselves.

The physical rack supports or shelving units are made of welded and powder-coated aluminum and hold up well to the weight and corrosive properties of seawater (Fig. 1a). The typical rack can be constructed with either five or six shelves depending on the configuration of tanks and has a footprint of approximately 5′-6″ wide × 1′-4″ deep. The configuration with six shelves is 7′-8″ tall and needs to be anchored either to the wall or to the ceiling to prevent it from accidentally tipping over (see Fig. 1b). Smaller racks with fewer shelves can also be constructed, which could even be installed on a sturdy table or benchtop. Other components, such as the tanks and plumbing, are made of various types of plastic and glass that can withstand saltwater (see Fig. 1a). There are very few metal components in direct contact with the seawater, which are made of either titanium, stainless steel, or ceramic ferrite, and these are described in the SOP (Additional file 1, Section 31: Metal Components Exposed to Sea Water). These parts have very good corrosion resistance. When filled to capacity (about 80 gal or 302 l of seawater), the system is very heavy (approximately 1100 lb or 500 kg). Therefore, the floor must be strong enough to support this weight. As the rack is supported by only four small round leveling feet, it could eventually cause impressions in the flooring. As a precaution and to distribute this weight, we machined a set of four ½-inch-thick, 4″ × 4″ aluminum pads with central recesses for the leveling feet (see Fig. 1c, Additional file 3A). Ideally, this type of system should be installed on lower levels in a room with drainage in the floor, to reduce the potential for damage caused by leaks and accidental flooding.

### The individual tanks and culture tubes

The rack holds multiple individual polycarbonate tanks (see Fig. 1a, d, e). Tanks provided by Iwaki are manufactured by Pentair Aquatic Ecosystems (Apopka, FL), as well as other manufacturers. These tanks sit on the metal crossbars (shelves) of the aluminum rack and are secured by ridges molded into their bottoms that engage these crossbars to prevent them from moving (Fig. 1e). These tanks are easily removed for access and cleaning, and come in many different sizes. The rack can be designed to hold tanks as large as 32 l. The particular system described here consists of six 10-l tanks on the bottom shelf and sixty 3-l tanks on the top five shelves (12 tanks per shelf, see Fig. 1a). These tanks accept slotted or Nylon mesh “larval” baffles (Fig. 1f). The larval baffles are available from Iwaki in three mesh sizes (400, 700, and 1000 μm). While smaller mesh sizes could be installed by the user, they could have an impact on flow through the system and would require more frequent cleaning. These baffles slide into molded channels in the back of each tank, and their purpose is to prevent animals and embryos from reaching the rear drain tube where they could travel to other parts of the system. For our particular purposes, we use baffles with 400-μm openings, which are small enough to prevent *C. atrasolea* embryos or juvenile snails from escaping the tanks. Even if the juvenile snails could escape from these tanks, it is not possible for them to travel to other tanks, as the animals would ultimately be stopped by either the coarse filter pad located over the sump or the 50-μm pleated filter (described below, see Fig. 2).

Each tank is equipped with a removable lid that has four ½″ diameter holes (Fig. 1e, g). These holes accept ¼″ OD tubing from the manifold valves that supply water to each tank. As there is only one valve/tube for each of the small tanks and two for the larger tanks, we cap the unused holes with plastic snap caps to limit evaporation (see Fig. 1g). Seawater flow to the tanks is regulated by adjusting the PVC valves fitted to the manifolds (Fig. 1d, see Additional file 1, SOP, Section 4: Water Flow). Each tank has a single round drain tube at the rear, which directs the overflowing seawater to drain troughs (Fig. 1e, h). As supplied, these long troughs are open along the top. To limit evaporation, we machined a pair of recessed 1/4″-thick PVC plastic covers for each trough, with holes to accommodate individual tank drain tubes (see Fig. 1h, i; Additional file 3B-C). Another PVC cover caps the main drain conduit at the very top of the system, which is provided by Iwaki Aquatics, Inc. To keep track of each tank, their locations are marked using small adhesive plastic labels (P-touch TZe tape, Brother, Nagoya, Japan, see Fig. 1d).

The *Crepidula* snails are kept in small screened culture tubes, as previously described [9] (Fig. 1d), with open ends covered by fiberglass screens. The increased flow rates in the automated system have the added benefit of preventing biofilms from accumulating on these fiberglass screens. Because the culture tubes are positively buoyant and can collect air bubbles, we keep them submerged using a curved, stiff piece of Nylon plastic mesh (Darice Canvas Designer Ultra Stiff Plastic Canvas, Strongsville, OH). Pieces are cut into rectangles, 7–1/4″ × 9″ and curved to form an arch that is pressed into the tanks to keep the tubes submerged (see Fig. 1d, j). The stiffness of this particular mesh helps it grip the sides of the tanks to keep it secure. The configuration of culture tubes placed inside individual tanks provides two levels of isolation for the adults and makes it easier to keep track of particular snails. Culture tubes are also labeled to keep track of the animals inside them. It should be noted, however, that newly hatched juveniles can pass through the fiberglass screens (which have mesh openings of about 1 mm^2^), and thus may travel between culture tubes housed in the same tank. Sufficiently large openings are necessary for efficient water flow through the tubes. Each 3-l tank can readily hold at least 10 tubes, and the 10-l tanks can hold at least 30 tubes, though we generally keep them at a lower density. As each tube can hold a single animal, a mated pair, or even several adults, one can house hundreds to many thousands of adult snails in this aquatic rack system. Given that these animals are rather small, even at full capacity, the bio-load will be relatively light and should not exceed the generous filtering capacity of the system (described below).

### Seawater

For growing *Crepidula* snails, we use Instant Ocean Reef Crystals artificial sea salt (Spectrum Brands, Inc., Blacksburg, VA). This seawater is mixed using reverse osmosis deionized (RODI) water to a specific gravity of 1.024 and a final pH of 8.2 to 8.3 at 27 °C. We also add Replenish (Seachem, Madison, GA), following the manufacturer’s instructions, to restore general hardness to RODI water. Instant Ocean Reef Crystals sea salt is formulated with higher concentrations of calcium and trace elements. We found that snails raised using the standard formulation of Instant Ocean sea salt developed shell defects [9].

### Water circulation

Ultimately, all of the recirculated water from the tanks returns by gravity back to the sump located at the bottom of the system (Fig. 1k, Fig. 2). Water circulation is provided by a seal-less magnetic drive pump (MD-70RLZT, Iwaki, Aquatics, Holliston, MA) with a polypropylene centrifugal impeller (Fig. 1l). This particular 2/7 hp pump has a rather steep pump curve with a maximum capacity of 11.4 gal/min and a maximum head pressure of 66.6 ft. This pump can deliver a maximum system pressure of 42.7 psi. This pump was specifically chosen, as it can deliver higher pressures while maintaining relatively high flow rates through individual tanks during both filtration and feeding cycles. Note that the intake for this pump, which is located in the left compartment of the sump, has a slotted plastic strainer to prevent large objects from being sucked into the pump. When the filters are being used under normal operation, the flow rate is typically around 30 lpm. This rate climbs to about 33 lpm during a feeding cycle when the filters have been diverted (note that these values depend on many different factors). The flow rate is adjustable depending on how the various valves are set and the age/status of the 50-μm pleated filter. The flow rate will drop as this filter becomes clogged. When only the bottom three rows of tanks are being used, the system has a volume of about 45 gal (170 l), and this volume is exchanged once every 4–8 min. This allows for 7–13 water cycles every hour. The pump should never be allowed to run dry. Several safety features (flow sensors) are built into the system to prevent this from occurring, which are described in detail below and in Additional file 1, SOP.

If the main pump fails or the flow meter detects a problem, water flow through the system would stop. However, the individual tanks drain from the top (Fig. 1e, f), which was designed to ensure that the tanks will maintain their water level even if the pump stops or the sump should run dry from a leak. *Crepidula* snails and many other marine organisms can tolerate stagnant water for many hours, or even a day or two. The flow sensors and sump ultrasonic level detector would communicate any such problems to the users in time to address these problems. The only issues would be that the tanks’ water temperature would slowly change to ambient temperature, waste products would begin to accumulate, and the oxygen saturation level would change, placing stress on the animals.

### Filtration

**Fig. 3:**
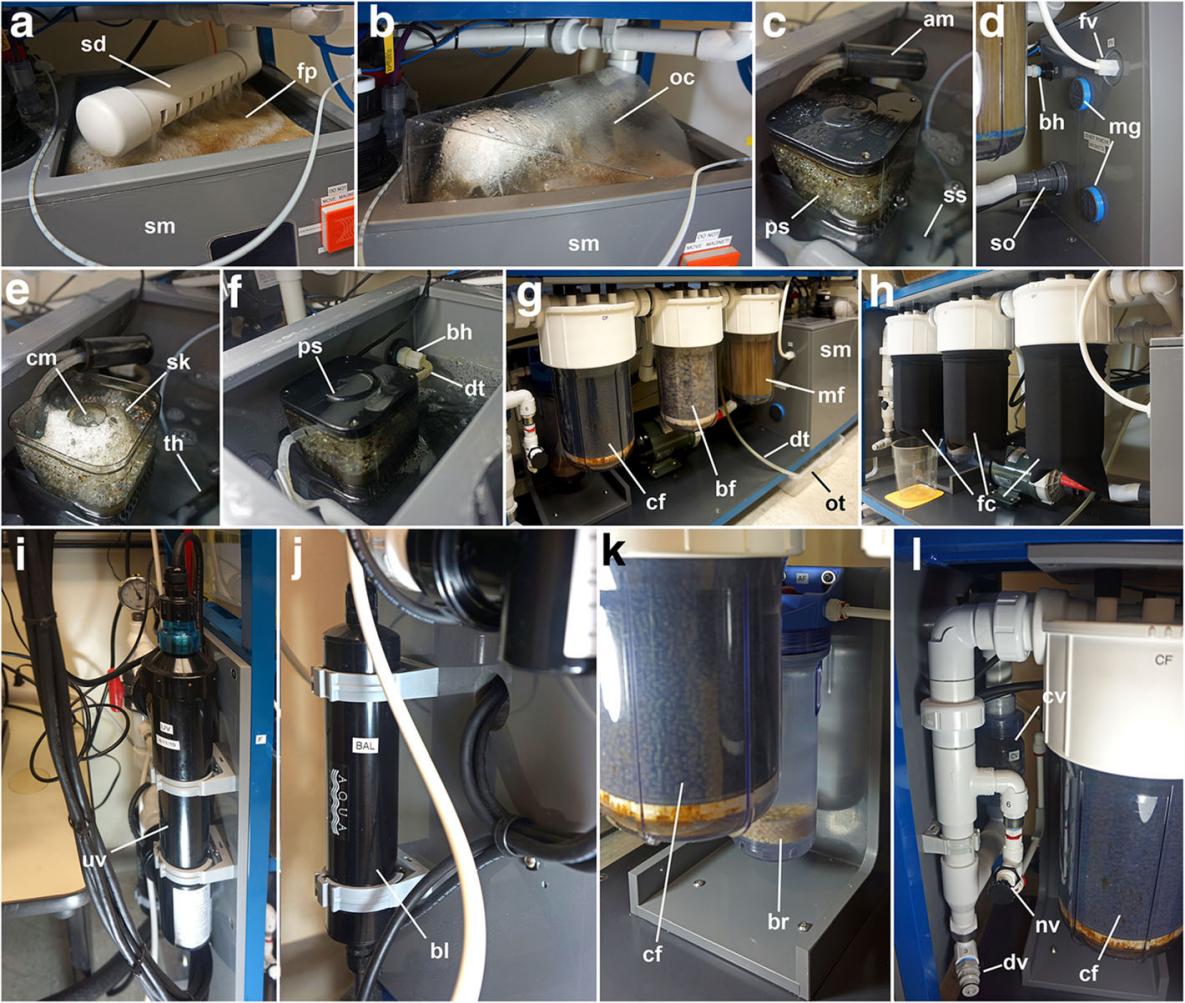
**a**–**l** Overview of the water filtration system. **a** Slotted drain diverter returning water to the right compartment of the sump through the coarse filter pad (origami cover has been removed). **b** View with the origami cover installed over the slotted drain diverter and large sump opening (see Additional file 3D). **c** Detail of the protein skimmer located in the left compartment of the sump. Small plastic sump lid has been removed to reveal the protein skimmer. The air muffler prevents water from entering the airline to the skimmer. Part of the perforated skimmer stand can be seen that supports the skimmer at the correct height. **d** Detail of the left side of the sump showing two magnets that hold the skimmer in place. The bulkhead compression fitting is also seen, through which the skimmer cup drain tubing passes to exit the sump. **e** View of the skimmer cup with its cover removed to show the chimney and the foamy skimmate, rich with organic materials. The titanium heater is also seen crossing the floor of the sump. **f** Another view of the protein skimmer. The skimmer cup drain tube is seen passing to the bulkhead compression fitting. **g** View of the three large canister filters. The skimmer cup drain tubing is seen passing to an overflow collecting tray located on the floor under the rack system. **h** Opaque black elastic covers for the canister filters, which are used to prevent algal growth. **i** UV lamp. **j** Ballast for the UV lamp. **k** View of the bioreactor located just behind the activated carbon filter. This filter canister is anchored by a PVC stand, and a small amount of the PHA pellets can be seen at the bottom of the filter bowl. This filter also has an opaque black elastic cover, which has been removed for this photo. **l** View of the small needle valve that controls water flow to the bioreactor to keep the PHA pellets in motion. The main system drain and the large one-way check valve are also seen. The latter prevents water from traveling backwards through the filters during a feeding cycle. am, air intake muffler; bf, biological filter; bh, bulkhead compression fitting; bl, ballast; br, bioreactor; cf, activated carbon filter; cm, chimney; cv, reverse flow check valve; dt, drain tubing; dv, main drain valve; fc, opaque filter covers; fp, coarse filter pad; fv, filler valve; mg, magnets; mf, fine mechanical filter; nv, needle valve; oc, origami cover; ps, protein skimmer; sd, slotted drain diverter; sk, skimmate; so, sump outlet; ss, skimmer stand; th, titanium heater. Other labels are the same as those used in Fig. 1

Water collected by the drain troughs returns directly to the sump through a slotted diverter (Figs. 2 and 3a, b). A plastic “origami” style cover limits splashing and evaporation (Fig. 3b, Additional file 3D). This water then passes through seven stages of filtration. First, it passes through a fibrous filter pad (Figs. 2 and 3a), which is suspended over the right compartment of the sump (1). This pad is located just below the main drain line’s slotted diverter. We find it useful to drape a thin piece of plastic (e.g., Saran wrap, S.C. Johnson & Son, Inc., Racine, WI) over the diverter to help limit splashing and to direct the water to the filter pad (see Additional file 1, SOP, Section 28: Sump Covers). This coarse mechanical filter pad is designed to trap only large debris and has an effective filtration size limit of around 200 μm. We replace the filter pad when we note that it is discolored and more water cascades over the pad than is passed through the filter. In our hands, the pad is replaced after 2–3 months of use, but the frequency will depend on the system load.

Once in the sump, seawater is filtered by the protein skimmer (2). The sump contains a compact Tunze protein skimmer (Tunze Comline DOC Skimmer, Penzberg, Germany) that fits just inside the left compartment of the sump (Figs. 2 and 3c). The protein skimmer is held in place by two strong rare-earth magnets supplied with the skimmer (see Fig. 3d). The skimmer is essential for removing organic waste and uneaten algae before these have a chance to break down and contribute to higher levels of ammonia, nitrites, and nitrates. The skimmer operates through the principal of foam fractionation (air-stripping), in which fine solids and dissolved organic compounds are adsorbed at the air-water interface of fine bubbles and concentrated in the resulting foam (Fig. 3e). To generate this foam, the skimmer produces a large quantity of very fine bubbles (to ensure lots of surface area). This particular skimmer is equipped with a DC motor and motor controller for precise speed regulation and production of fine air bubbles. The rising foam or “skimmate” floats to the top and is separated from the water to be trapped in a collecting cup (Fig. 3e). The skimmer is held at the correct height using a small adjustable perforated plastic platform with threaded plastic legs (skimmer stand), part of which can be seen in Fig. 3c. This platform sits right on top of the titanium heater (Fig. 3e). The height of the sump’s seawater in relation to the skimmer’s water intake is critical (Fig. 3c, d). Too little water and no skimmate will form. Too much water will also affect the formation of skimmate and cause excess water to be ejected into the collecting cup. As an added benefit, the numerous fine bubbles produced by the protein skimmer also help to aerate the seawater.

Skimmate needs to be removed from the collection cup regularly. Its rate of formation depends on several factors, including the size and amount of bubbles and the concentration of dissolved organics. We currently clean the skimmer cup and lid once each week or as needed. A thick sludge will build up inside these parts that must be wiped clean. Skimmate forms more effectively when the collecting cup’s “chimney” is clean (Fig. 3e). As supplied, the removable Tunze skimmer collecting cup does not have a port for attaching a drain line. Tunze sells a so-called Holiday Cup with such a drain port, but we made our own by installing a 3/8″ bulkhead compression fitting (see Fig. 3d, f). It is necessary to have the drain port installed in the proper location for the effective placement of the drain tubing. This fitting accepts silicone tubing (0.188″ ID × 0.375″ OD, Dow Corning, Midland, MI), which then exits the sump through another bulkhead compression fitting located just above the high water line on the left side (Fig. 3f). Great care must be taken to install these fittings so that the tubing is routed with the shortest path and does not get kinked. In addition, the effluent must flow downward and out of the system when the exposed external end of silicone tubing is lowered. With this configuration, one can use gravity to empty the collecting cup manually when it is full or simply run the tubing into a larger container or tray placed on the floor for continuous collection of the skimmate (Fig. 3g). One issue that might be encountered is that the sump bulkhead fitting (Fig. 3f) could become submerged below the high water line, whenever the system is placed in standby mode or the power is interrupted. Under these circumstances, some water could leak from the system if the fittings are not properly secured. During normal operation, this should not present a problem, as the fittings should be located just above the high water mark (assuming they have been properly installed).

Seawater in the sump is then pumped to a fine pleated mechanical filter (3) housed in a transparent canister. This filter has a very large surface area with a mesh size of 50 μm to remove fine suspended materials (Figs. 2 and 3g). This filter is replaced once the flow rate drops to about 20 lpm (flow should not be permitted to drop below 20 lpm). In practice, we find these filters each last 2–3 months, though as animal and feeding loads increase, filter replacements will become more frequent.

Next, the water passes to the main biological filter (4, see Figs. 2 and 3g). One of the problems that must be addressed in recirculating marine aquaria is the requirement to remove waste products, including the ammonia produced by the animals (via nitrification). Ammonia is very toxic, but nitrifying bacteria will convert ammonia to nitrites (also toxic) and finally nitrates, which are less toxic. The main biological filter is a substrate that includes a mix of 800 g of Matrix (Seachem, Madison, GA) and 100 g of de*Nitrate (Seachem, Madison, GA). These highly porous media serve as a substrate for aerobic and anaerobic bacteria to process ammonia, nitrite, and nitrate. This quantity of media has over 1400 m^2^ of surface area to support bacterial growth. Nitrates will steadily accumulate to toxic levels unless additional steps are taken to remove these compounds (either by periodic water changes or through denitrification, see below).

The water next passes to an activated carbon filter (5, see Figs. 2 and 3g). This canister contains approximately 0.5–1 kg (1–2 lb) of activated carbon pellets (Kent Reef Carbon Pellets, Kent Marine, Franklin, WI). Activated carbon is a porous substance with a very high capacity for absorbing organic materials. The carbon pellets can also serve as a substrate for nitrifying bacteria. There is much debate about the use of activated carbon in marine aquarium systems. Activated carbon is highly effective at removing organic materials; however, some authorities recommend using far less. Activated carbon can remove important trace elements. In addition, and depending on the source, activated carbon can affect the pH and elevate the levels of phosphate in the seawater. To prevent algal growth within the various canister filters, we cover the transparent bowls with sleeves made from opaque (dark) Nylon Spandex fabric (Fig. 3h).

Most of the water leaving the activated carbon filter passes to a plastic canister containing a UV light protected by a quartz sleeve (6, see Figs. 2 and 3i) before it is distributed to the individual tanks. The electronic ballast is located behind this lamp (Fig. 3j). The UV light helps sanitize the water by killing circulating microorganisms. The sanitizing efficiency of UV light is related to UV intensity and water flow rate (e.g., exposure time). UV output diminishes with use, and therefore, the UV bulb should be replaced yearly. In addition, the quartz sleeve that encloses the UV bulb must also be inspected and cleaned whenever the bulb is changed (see Additional file 1, SOP, Section 12. Servicing the UV Lamp). The sleeve only needs to be cleaned when deposits have formed. Note, that we have not yet found a need to use the UV light in our system. However, UV sterilization may be required in coastal environments where natural seawater may be available for use in the system, or if disruptive algae, ciliates, or other protozoans are present in significant and harmful levels, and/or exposure to airborne contaminants (e.g., fungal spores) become problematic.

The final stage of filtration helps remove nitrates from the system. Generally, nitrates are removed from marine aquaria via regular, partial water changes [25]. To reduce this requirement in our system, some of the water leaving the activated carbon filter is diverted to a rear side loop containing a fluidized bioreactor (7, see Figs. 2 and 3k). This smaller filter bowl contains a very small quantity (approximately 60 g, see Additional file 1, SOP, Section 10.4: Servicing the Nitrate Bio-Reactor) of biodegradable polyhydroxyalkanoate (PHA) polymer pellets (Vertex Aquaristik Pro-Bio Pellets, Huntington Beach, CA). These polyester pellets provide a source of organic carbon to support bacterial growth of anaerobic and aerobic bacterial strains that accomplish the removal of nitrates. Only a small amount of these pellets can be used to limit the formation of potentially toxic levels of hydrogen sulfide, and they must be kept moving (slowly) to prevent sticking and excess formation of the bacterial biofilm. Too much PHA media, too little water flow, or insufficient levels of oxygenation can create toxic levels of hydrogen sulfide. A small needle valve allows for precise adjustment of the water flow (Figs. 2 and 3l). It is desirable to keep this filter in the dark or covered, as mentioned above. Water from this side loop is returned directly to the sump (Fig. 2), near to the inlet of the protein skimmer, which helps remove the released biofilm and other byproducts of these bacteria. In addition, the inclusion of some de-nitrate granules in the biofilter (5, see above) also helps to remove nitrates. Despite these precautions, sudden increases in the levels of toxic ammonia or nitrites, or excess levels of nitrates may occur in any system. For these potential events, the aquatic rack system includes a main drain port, as well as a filler port that can be used for water exchanges (Figs. 2 and 3d, l).

### Establishing the biological filter

When starting a new system, one must inoculate the biofilter with nitrifying bacteria and cycle the system. Many references explain how to complete the ammonia cycle and monitor these byproducts (e.g., [26, 27]). This process simply establishes a population of bacteria that are able to convert toxic animal waste (ammonia) into less harmful products [28]. Ammonia provides a substrate for ammonia-oxidizing bacteria, which convert ammonia into nitrite, which in turn provides a substrate for nitrifying bacteria which convert nitrite to nitrate. While adding a hardy fish to an uncycled aquarium is one way to provide a source of ammonia to establish the biological filter, this is highly stressful to the fish and is not recommended. The addition of regular doses of ammonium chloride solution is sufficient to support the growth of nitrifying bacteria and does not require the discomfort and possible death of fish to establish the biological filter. For details on establishing the biological filter, see Additional file 1, SOP, Section 38: Inoculating the System. Briefly, ammonia levels are increased to 2–3 ppm until nitrate levels begin to increase, and low doses of ammonia, in the form of either ammonium chloride (half the initial dose, 1–1.5 ppm ammonia) or wastes from the hardy fish species, are added until both ammonia and nitrite levels return to 0. You will know that the system has successfully cycled when the addition of a full dose of ammonia (2–3 ppm) is rapidly converted to nitrate, and ammonia and nitrite levels remain at 0 ppm. At this point, you may begin adding animals to the system. This should be done gradually, so as not to overload the biological filter. Commercial ammonia-removing products should not be used in the system while the biological filter is being established. In addition, the UV lamp must also be turned off during this process.

The addition of commercial products containing ammonia- and nitrite-oxidizing bacteria starter cultures may be used to introduce the bacteria, although some authorities question their effectiveness [29]. There are several commercial products available to inoculate the biological filters. We used both: Seed Bacteria (Aquavitro, Seachem, Madison, GA) and Dr. Tim’s One and Only Live Nitrifying Bacteria (Dr. Tim’s Aquatics, LTD, Moorpark, CA). Instructions for using these products are provided by the manufacturers (see Additional file 1, SOP, Section 38: Inoculating the System). In any case, patience is required, as these bacteria grow slowly. The full establishment of the biological filter can take several weeks to months. In the case of our system, the initial cycling of the biological filter took 6 weeks, with the addition of commercial bacterial cultures.

We have found that this aquatic rack system provides a much greater level of filtration, compared to our previous setup [9], which consisted of nothing more than a 30-gal glass aquarium with an under-gravel, biological filter. The filters in the aquatic rack system greatly reduce the buildup of nitrates in the seawater, lessening the requirement to carry out seawater changes. After the first 6 months of operation of this system, we found no detectable levels of ammonia, nitrites, or nitrates, and no need for water changes. Levels of nitrates in our earlier glass aquaria continued to climb, and we recorded levels over 160 ppm, which necessitated periodic water changes. We estimate the current population of the rack system at 2000 animals, in a volume of approximately 45 gal (170 l). At the time of submission (10 months of operation), we find no detectable levels of ammonia or nitrite. Nitrate levels have increased slightly, with maximum levels measured at 5–10 ppm, well below the levels recorded in our previous system, and within safe operational parameters.

### Sensor systems and the maintenance of water quality

**Fig. 4:**
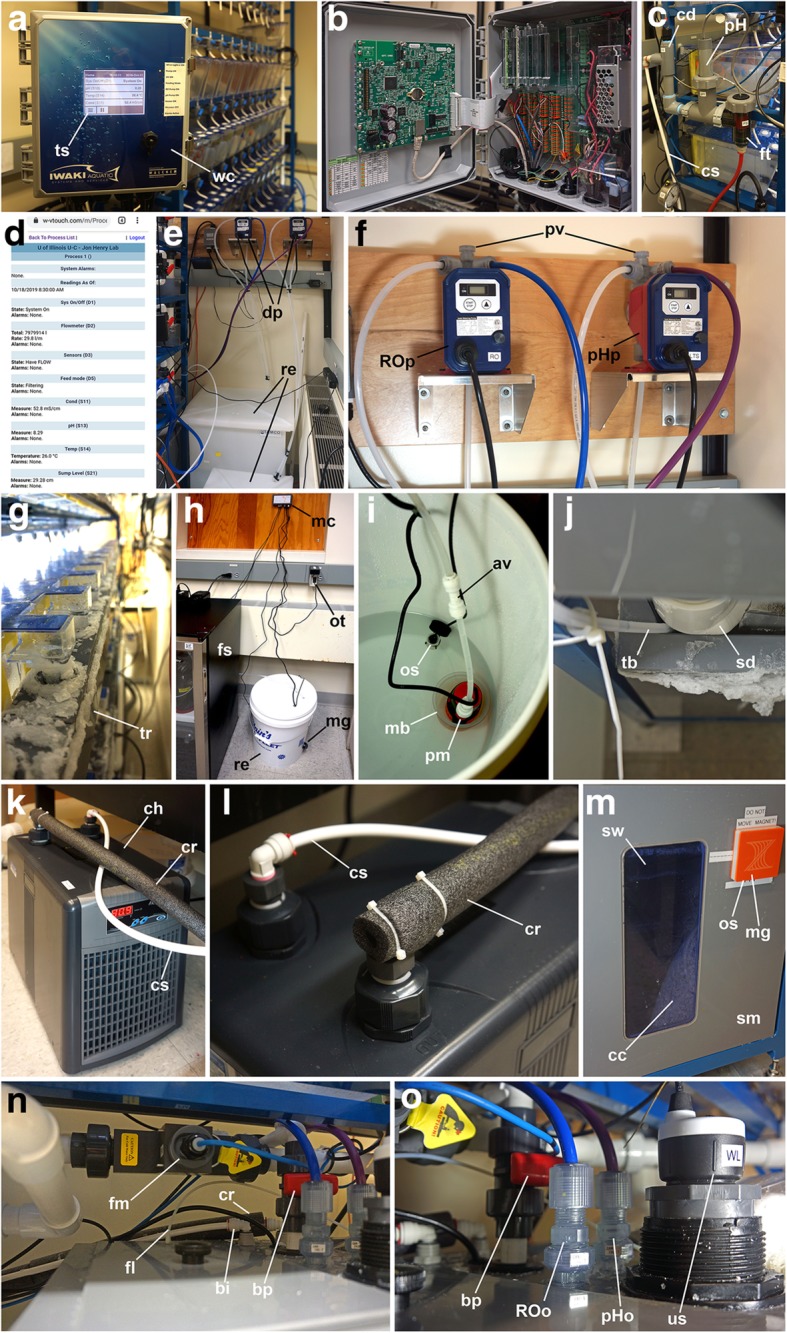
**a–o** Overview of the Walchem 900 controller, main sensors, dosing pumps, and the external chiller. **a** Walchem 900 controller and touch pad interface. **b** View inside the Walchem controller showing many electrical connections to the various sensors and dosing pumps, etc. **c** View of the sensor side loop. Water for the side loop is supplied by the red plastic tubing and passed to the external chiller by the white plastic tubing. **d** Example showing some of the information provided by the system, which can be viewed online. **e** View of the RODI water and pH/calcium dosing pumps and their reservoir, which are situated to the right of the rack system. **f** Close up view of the RODI water and pH/calcium dosing pumps, which are mounted on the wall. **g** Example of salt creep on one of the drain troughs. **h** View of the automatic seawater top off (ATO) unit. **i** View inside the seawater reservoir for the ATO unit showing the small submerged pump and optical water level sensor. **j** Location of the ATO unit supply line that directs seawater to the back right corner of the sump, just behind the slotted drain diverter. The tubing is secured with a pair of zip ties. Note evidence of salt creep. **k** View of the external chiller. Water for the chiller is supplied via the sensor side loop. The cold return line is covered with foam insulation. **l** Close-up view showing the custom fittings and right angle push connectors for the chiller’s water lines (see Additional file 3E). **m** View of the small window located in the sump, through which one can readily asses the water level. The Nylon mesh bags containing crushed coral can be seen inside the sump. The square magnet that secures the optical water level sensors for the ATO unit is also visible. The dual optical sensors are located inside the sump. **n** View of the flow meter and various connections to the sump. The food line is seen entering the sump through a small hole drilled in the PVC plastic lid that covers the left compartment. **o** View of the Echopod ultrasonic water level sensor and other connections to the sump. av., anti-siphon valve; bi, bioreactor return line; bp, bypass valve; cc, mesh bags containing crushed coral; cd, conductivity sensor; ch, chiller; cr, chiller return line; cs, chiller supply line; dp, dosing pumps; fl, feeding line; fm, flow meter; ft., float switch; mb, Nylon mesh basket; os, optical water level sensor; ot, outlet timer switch; pH, pH probe; pHo, pH dosing pump outlet; pHp, pH dosing pump; pm, ATO seawater dosing pump; pv priming valves; re, reservoir; ROo, RODI dosing pump outlet; ROp, RODI water dosing pump; sd, salt deposits; sw, sump window; SWo; seawater dosing pump outlet; tb, tubing from ATO unit; ts, touch screen; us, ultrasonic water level sensor. Other labels are the same as those used in Figs. 1 and 3

This aquatic rack system is equipped with a Walchem 900 controller with various sensors (Iwaki Aquatics, Holliston, MA) to monitor the system parameters and to adjust water quality (Fig. 4a–c). Together, this equipment monitors and regulates temperature, salinity, and pH. Other sensors monitor water flow and sump water level, which are also described below. Seawater levels in the sump are controlled separately, as described below. The Walchem 900 controller communicates with the feeding system’s Arduino microcontroller to activate a bypass valve during feeding and to control power to the protein skimmer (see further discussion below). The Walchem 900 controller also has an ethernet connection to the local network to communicate system parameters and any alarms that may be triggered when there are problems. A program and user application designed by Iwaki Aquatics (www.w-vtouch.com) allows one to monitor all conditions in real-time and to receive push notifications by email and text messages to announce any problems. Users can log into the system online from anywhere in the world to observe and change the system parameters and view the recorded data (Fig. 4d). Users define set points for regulating various seawater parameters and set various high and low levels for triggering alarms. When an alarm is triggered, users are immediately alerted to the specific problems so that they can have time to correct them before the animals are harmed. All system parameters are recorded, and running logs are sent to users on a weekly basis.

### Maintaining the proper salinity and water level

It is essential to maintain the proper salinity for the health of any marine organism. Normal seawater salinity ranges from 33 to 37 ppt or a specific gravity (SG) of 1.020 to 1.029. Conductivity is monitored by a probe in the sensor side loop (Figs. 2 and 4c). We maintain salinity at 33 ppt or a SG of 1.024. Evaporation from the system continuously reduces water volume and increases salinity. The rate of evaporation is related to several factors, including the exposed water surface area, and the relationship between water temperature and the room temperature and humidity. To correct this situation, the sensor loop has a conductivity sensor to monitor the salinity, and an electromagnetic diaphragm pump automatically adds RODI water to lower the salinity, as needed (Figs. 2 and 4e, f). The large RODI water reservoir typically holds enough water to last for at least 2 weeks before needing to be refilled. In addition, we limit evaporation by covering any exposed openings by the addition of covers for the drain troughs and the exposed openings of the sump. The latter includes the folded, polycarbonate “origami” cover (Fig. 3b) located over the drain diverter. It is fashioned from a single piece of 1/8″-thick polycarbonate folded on a sheet metal brake (see Additional file 3D for the diagram). Note that this opening is not completely sealed, and this allows some air circulation at the corners (Fig. 3b), which is necessary for oxygenation in this system. It should be noted that there is an unintended side effect of adding these covers, which is that they tend to trap more heat inside the system (see Additional file 1, SOP, Section 27: Preventing Evaporation, for details and warnings).

With flowing water and other active forms of aeration, there is some splashing, spray, and mist. This causes some seawater to leave the system and to penetrate various seams where it evaporates and leaves condensed sea salts, a condition commonly referred to as “salt creep” (Fig. 4g). Once enough salt builds up along various seams, tank lids, etc., it begins to act like a wick. This accelerates further salt buildup, and some water may begin to drip out of the system. From time to time, these salt deposits will need to be removed. We currently do so every 1 to 2 months, as needed. This gradual loss of salt slowly reduces the system’s water volume, which must be replaced. As mentioned above, a critical water level is required for the proper operation of the protein skimmer. To maintain a constant water level and to replace lost salts, an automatic top off system is used. While the sump is equipped with a mechanical float valve for topping off the system, these types of mechanical valves are somewhat unreliable. Instead, we have installed an automatic top off unit that uses redundant optical sensors to detect changes in the water level and a small electromechanical pump (Figs. 2 and 4h). Many different systems are available on the market, and we chose to use the ATK system manufactured by Neptune Systems (Morgan Hill, CA). This system operates in a stand-alone fashion and has redundant optical sensors and other smart features to prevent accidental overfilling of seawater. Instructions for setting up the pump are provided by the manufacturer and described in Additional file 1, SOP, Section 33: Automatic Sea Water Top-Off System. The sensors are held in place by powerful magnets. Depending on the rate of salt loss, this unit only runs once every 3 to 5 days. Seawater is stored in a 5-gal plastic bucket (Figs. 2 and 4i). This reservoir typically supplies enough seawater for at least 2 weeks. Because this unit would run during a feeding cycle when the water level in the sump temporarily drops, we have put the ATK on a timer so that power is only supplied once a day for 30 min during a filtering cycle. An additional optical sensor (OS-1-M, Neptune Systems, Morgan Hill, CA) is also used to monitor the level of seawater in the reservoir bucket (Fig. 4i). This sensor is also held in place with a small magnet and plugs directly into the ATK microcontroller (port number 4). When the water gets too low, an alarm is sounded and the pump will not run. However, the alarm can only be heard during the narrow 30-min window when the unit is actually powered, and the level of seawater in the top-off bucket should be monitored by the user. The seawater is pumped directly to the back corner of the sump, just behind the slotted drain diverter (Figs. 2 and 4j).

Note that there are some simple steps one can take to help limit salt creep, which seems to be mainly related to areas with splashing seawater. For example, one can lengthen the plastic tubing that supplies seawater to each tank. If the ends are located below the water surface, there will be less splashing and less salt creep will be found around the tank lids and plugs. The disadvantage of this is that one is not then able to readily observe the rate of water flow to each tank.

### Maintaining the proper temperature

It is very important to maintain a constant temperature that is optimal for rearing a particular species. It is generally easier and more cost-effective to maintain a low room temperature and regulate water temperature using a heater. *C. atrasolea* snails are warm water snails found in the southern waters of Florida, USA. While they can withstand a fairly wide range of temperatures, we maintain these snails at 27 °C. There are two temperature sensors located within the sensor side loop. One is associated with the pH probe and the other with the conductivity probe (Figs. 2 and 4c). Temperature regulation is mainly accomplished using a titanium heater located on the floor of the sump (Figs. 2 and 3e). Titanium is rather impervious to the effects of seawater. Other components of the aquatic system also generate heat. These include the main water pump, the UV light, and, to a much lesser extent, the friction encountered by the flow of water through the system. On the other hand, cooling can occur via the large open surface area of the water in the system and exposed surfaces of the tanks and plumbing. Cooling also takes place via evaporation, and this will be related to the temperature differences and the relative humidity in the room. Depending on the conditions, a chiller may also be necessary to lower the water temperature, especially if the aquatic system cannot be housed in an air-conditioned room or there is any possibility that the room temperatures will climb above 22–23 °C, or for maintaining temperate species that require much lower water temperatures. Iwaki can incorporate a chiller in the aquatic rack system where the titanium cooling coils would be installed inside the sump. This situation would be ideal, as the Walchem 900 controller can be used to regulate both the heater and the chiller. On the other hand, the presence of the cooling coils may interfere with the placement of other equipment in the sump, such as the protein skimmer. An external titanium chiller entails less expense and is the option we chose (e.g., Artica Titanium Chiller, DBA-075, JBJ Aquarium, Inglewood, CA, see Figs. 2, 4k). Depending on the model, one may have to provide pressurized water to the chiller, which can be accomplished by running a water line from the sensor side loop. We simply hooked the chiller to the water that is being expelled from the sensor side loop using existing connections and some extra 3/8″ plastic tubing (Figs. 2 and 4c, k, l).

With this arrangement, one must note that there are some important concerns that must be addressed. In our case, the external chiller is present to serve only in an emergency when the room temperature gets too warm. This arrangement has been fully tested and works very well for this purpose. On the other hand, we do not know how this external chiller would do operating for longer durations in hot environments. The external chiller has its own thermostat, separate from the circuit used to control the heater. Therefore, the temperature set points must be adjusted so that the heater and chiller do not end up fighting one another. The main system heater temperature control is set to go on at 26.75 °C and off at 27.25 °C, which is around 79–80 °F. There are two temperature sensors in the system: one for the conductivity sensor and another for the pH probe, and either one can be set to control the heater. To avoid activating the heater, and vice versa, the chiller is set to turn on only when the temperature reaches 82 °F (27.78 °C) and will go off at 81 °F (27.22 °C). Note that this setting is stored permanently in a memory should the chiller lose power. Users must follow the manufacturer’s instructions to calibrate the chiller temperature sensor. To use the chiller with the 3/8″ tubing supplying the sensor side loop, custom threaded pipe fittings had to be machined to replace the barbed hose elbows supplied by Artica (see Fig. 4l and Additional file 3E). They are machined from solid PVC plastic, drilled, and tapped for ¼″ right angle polypropylene compression fittings (McMaster Carr, Elmhurst, IL) to receive the 3/8″-diameter tubing from the sensor side loop and return the chilled water to the sump (Figs. 2 and 4 k, l, n). These new PVC fittings use the same rubber gaskets and hose compression nuts supplied by Artica.

### Maintaining the proper pH

pH is monitored by a probe in the sensor side loop (Figs. 2 and 4c). Normal seawater pH ranges from 7.5 to 8.4. We maintain the seawater at 8.3. Buffers in the artificial sea salts do a good job maintaining this pH. In addition, we placed two Nylon mesh bags containing crushed shell and coral debris in the right sump compartment to help buffer the seawater (Figs. 2 and 4m). These also provide additional refugia for bacteria and a source of calcium for snail shell deposition. On a few occasions after we initially set up the system, we saw the pH drop by 0.1 unit. When this happened, we added 5 g of Marine Buffer (Seachem, Madison, GA) per 40 gal of seawater to help elevate the pH by 0.1 unit. So far, pH has been maintained at a fairly constant level (8.3). The Iwaki aquatic rack system is equipped with a second reservoir and diaphragm pump for pH adjustments or to add calcium to the system (Fig. 4e, f). Typically the stock solutions that can be added to buffer the pH (e.g., Kalkwasser and Na^++^ bicarbonate) are fairly concentrated. Fearing potential problems, we have not used this feature. In the future, we may use this equipment to supplement Ca^++^ levels in the system on a weekly timed basis, which we now perform manually. Particularly, for animals that secrete calcareous materials, calcium levels, pH, and KH (general water hardness) should be measured regularly (weekly) and adjusted as necessary (see Additional file 1, SOP, Section 11: Water Quality).

### Monitoring water flow

A simple electromagnetic float valve monitors the presence of water flow through the sensor side loop (Figs. 2 and 4c). A separate flow meter also monitors the actual flow rate through the entire system (Figs. 2 and 4n). If these sensors detect reduced flow, an alarm would be sent, and the main water pump will be turned off. Likewise, the sump has an ultrasonic water level sensor to detect for low water or flooding conditions (Figs. 2 and 4 o, Flowline DL10-01 Ecopod, Los Alamitos, CA). If the sump water level falls below a certain level, the main water pump will be shut off.

### Automated feeding

**Fig. 5:**
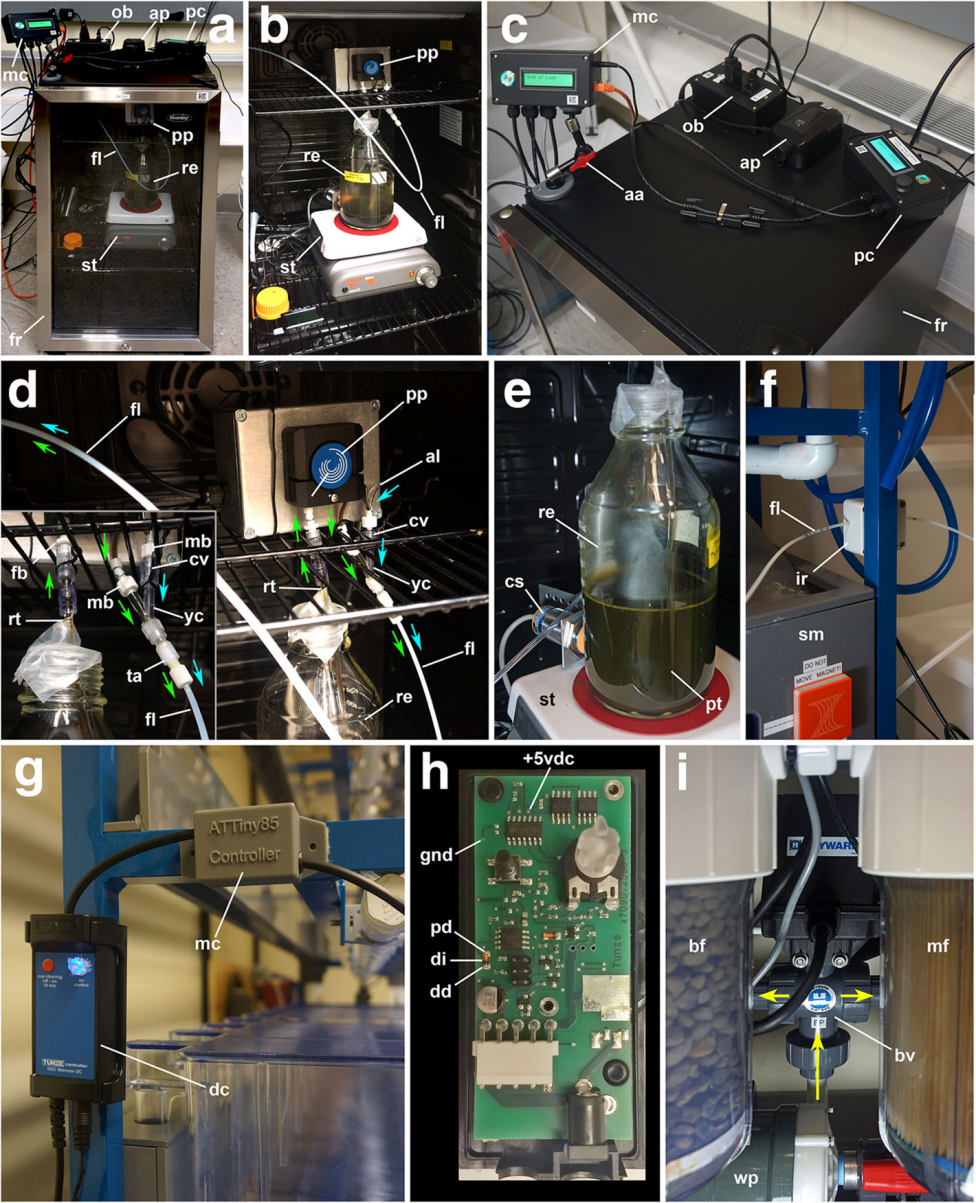
**a**–**i** Automated feeding system overview (see also Additional files 3, 4, 5, 6, 7, 8, 9, 10, 11, 12). **a** Front view of the feeding system and mini-refrigerator. **b** View inside the mini-refrigerator showing the equipment used to deliver the food. **c** Top view showing the feeding system microcontroller, 120-V AC outlet relay box, air pump, and peristaltic pump controller. The power cords for the stir plate and air pump are plugged into the relay box, and the two outlets are switched on and off separately by the microcontroller. **d** Close-up view of the peristaltic pump and associated Luer lock fittings that make up the food pump (see also Additional files 13, 14). Green and blue arrows show the directions of food and air flow, respectively. **e** The 1-l glass food reservoir sits on the stir plate. A non-contact, capacitance sensor monitors the level of liquid food inside the bottle (see Additional file 3F-H). The height of the bracket holding this sensor is adjustable to set where the alarm will trip (e.g., low food level). The bracket slides on a metal post that is screwed into a threaded hole located on the base of the stir plate. **f** View of the IR sensor that detects the presence and absence of food in the Teflon feeding line (see Additional files 9, 10, 11, 12). **g** View of the DC motor controller for the protein skimmer and a small microcontroller unit that regulates power to the skimmer (see Additional files 15, 16, 17, 18). **h** High magnification view of the Tunze DC motor control board showing the points where 30-gauge wire connections are made to the microcontroller (see Additional file 15). The small diode is removed and used to build the microcontroller. **i** View of the motorized bypass valve located behind the mechanical and bio-filters. Yellow arrows show two possible routes of water flow from the main water pump, below. The motorized bypass valve can direct water either to the right and through the various filters or to the left and through the bypass loop (during feeding). aa, articulated arm; al, air line; ap, air pump; bv, motorized main bypass valve; cs, capacitance sensor; dc, dc motor controller; dd, distal diode pad; di, diode; fr, mini-fridge; gnd, ground connection; ir, IR food sensor; lf, Luer lock fittings; mc, microcontroller; ob, outlet relay box; pc, peristaltic pump controller; pd, proximal diode pad; pp, peristaltic pump; pt, phytoplankton; rt, reservoir tubing; st, stir plate; ta, Teflon tubing adapter; yc, Y connector; +5vdc, + 5-V supply connection. Other labels are the same as those used in Figs. 1, 3, and 4

*Crepidula* snails are filter feeders that mainly eat suspended phytoplankton. As such, they presumably have nearly continuous access to food in the wild. However, it is not practical to manually feed animals repeatedly throughout the day. To reduce the workload and supply regular feedings, we have incorporated an automated feeding system (Fig. 5a, b). In a recirculating system, it is not possible to supply food continuously, as the filters must also run to remove organic waste materials. We balance these requirements by switching the water flow between the filtration loop and a bypass feeding loop (see below). Fortunately, the design and fabrication of robotic systems for the systemic delivery of liquid foods are much easier to accomplish compared to the delivery of solid foods to individual tanks. What makes this approach viable is the availability of commercial preparations of phytoplankton and other liquid foods that can be kept in the refrigerator at 4 °C for several months. While one can grow live algae, commercial preparations of preserved algae, such as Phytofeast and Shellfish Diet (Reef Nutrition, Campbell, CA), offer great convenience. Their suspended, liquid form makes it possible to deliver precise doses of food to the aquatic system.

The feeding station consists of a small beverage cooler housing the food reservoir, a stir plate, a peristaltic pump, and Luer lock fittings needed to pump the food (Fig. 5b). This equipment is controlled by an external customized microcontroller, which is mounted to the top of the refrigerator, together with a remote relay box and a custom peristaltic pump controller (Fig. 5c). Associated sensors monitor the food supply and food delivery. Details regarding the construction and programming of these microcontrollers and peripheral components are provided in Additional file 1 (SOP, Section 34: Feeding System), 2, 3F-H, and 4-14. This microcontroller communicates directly with the Walchem 900 controller during feeding cycles to activate the bypass valve and cut power to the protein skimmer. For very precise and reliable pumping of this algae, we chose to use a small peristaltic pump (Fig. 5d). This peristaltic pump is mounted in an aluminum box and uses a precision stepper motor to ensure precise delivery of smaller, consistent volumes. The peristaltic pump is controlled by a second custom microcontroller unit which can be programmed using its own front panel interface for a set speed (rpms) and a number of steps (volume) (Fig. 5c, Additional file 1, SOP, Section 34: Feeding System, 2, 13-14). An input from the feeding system microcontroller triggers a single pumping cycle. The peristaltic pump can withstand the low temperatures of the refrigerator in which it and the food are housed. The commercial food stock (e.g., Phytofeast, Reef Nutrition, Campbell, CA) needs to be maintained at a recommended temperature of 4 °C to prevent it from spoiling. This food can be kept at even colder temperatures closer to 0 °C without fear of freezing. To keep the phytoplankton fully suspended in the reservoir (a one liter Pyrex bottle, no. 1395, Corning, Germany), a magnetic stir plate (Corning, Tewksbury, MA) and Teflon-coated stir bar are used (Fig. 5e). The stir plate is plugged into a remote relay outlet box, which is switched on and off by the feeding microcontroller (Fig. 5c, and Additional files 4, 5). This microcontroller activates the stir plate for 30 s once every hour and is timed to occur 5 min before food is pumped to the sump. The outlet silicone tubing is fitted with a barbed hose connection and a single Luer lock Y connector (Fig. 5d, and see below). The food is then passed to the aquatic system through 1/8″ OD Teflon tubing. A special flangeless compression fitting is used to attach the 1/8″ Teflon tubing to the Luer lock Y connector (HPLC waste line adapter, CPLabSafety, San Francisco, CA, Fig. 5d). This tubing is directed to the left sump compartment through a hole drilled in the top of the small removable PVC sump cover located directly above the aquatic system’s main water pump intake (Figs. 2 and 4 n). To purge the Teflon feeding line and ensure full delivery of the food, a small aquarium air pump is used, which is activated automatically by the feeding system’s microcontroller after the food is pumped into the tubing (Fig. 5c, d). The air passes to the Luer lock Y connector through a one-way check valve (Qosina, Ronkonkoma, NY; Cole-Parmer, Vernon Hills, IL, Fig. 5d). The check valve prevents food from passing to the airline. Like the stir plate, this air pump is plugged into the remote relay outlet box (Fig. 5c). The feeding system is controlled by an Arduino microcontroller equipped with an ethernet shield for internet access. An illuminated LCD display and color-changing LEDs on the power switch communicate the time, feeding mode, and each step that takes place during food delivery (Fig. 5c). Sensors monitor the food levels (a non-contact capacitance sensor, Fig. 5e, Additional files 3F-H, 4, 5, 6, 7, 8) in the reservoir and successful food delivery through the feeding tube (IR detector, Fig. 5f, Additional files 9, 10, 11, 12) and alert the user of any problems that may occur via text messages or emails. This feeding system is very reliable, and we have had no problems with the delivery of the food (see Additional file 1, SOP, Section 34: Feeding System).

During each cycle, feeding is permitted for 24 min, and water is then redirected back to the filters for 1 h: 36 min. The volume of food and the feeding schedule can be adjusted, as needed within the program. Given a flow rate of around 33 lpm, with a system volume of 45 gal, it should take 5–6 min for the water to be exchanged throughout the system. Indeed, spectrophotometric measurements show that it takes about 5 min for the food to become evenly distributed within a system that contains 45 gal. This level of food is maintained for the remaining duration of feeding, and once the filters are reactivated, the level drops over the next 30 min until the food is essentially removed from the system, having been consumed, and/or trapped by the filters. These parameters (e.g., food concentration, length of feeding cycle) will need to be adjusted according to the size and number of animals in the system.

As mentioned above, the protein skimmer is critical for the removal of excess, uneaten food, and other organic materials, which otherwise add nitrogen to the system. However, to ensure that food is not removed during feeding, the skimmer is turned off automatically. To accomplish this, we designed a small enclosed microprocessor control circuit to interrupt the normal signal provided by the DC motor controller (Fig. 5g). The interface consists of an optocopler and a small ATTiny85 microcontroller. The parts list, circuit diagram (.fzz Fritzing diagram), program, and .stl files for 3D printing the plastic enclosure are included in Additional files 2, 15, 16, 17, 18, respectively. Figure 5h shows the points where connections are made to the Tunze DC motor controller PC board using a 30-gauge insulated wire that go the ATTiny85 controller (Fig. 5g, Additional file 15). Power to the skimmer motor is automatically restored when the feeding cycle ends.

As the food can get trapped by the various filters, we have incorporated a bypass loop which is activated during feeding (Figs. 2 and 5i). A motorized valve redirects water flow away from the filtration loop through a separate line. This water is prevented from returning backwards through the filters by a one-way check valve (Figs. 2 and 3l). When activated, the bypass valve allows water containing food to recirculate only through the small holding tanks and the sump, where the food is added. If the UV lamp is used, the water that circulates during feeding will be exposed to the UV light, as that lamp must continue to be cooled during use. Otherwise, the plastic housing would melt, water would leak, and the lamp would fail. UV lamp life is significantly shortened if it is turned on and off repeatedly, so it should be left on at all times or not used at all (see below). The effects of this UV irradiation on the food are unknown, and as mentioned above, we have not used the UV lamp equipped with our system.

Determining the correct amount of food to add, number of doses, and time intervals requires one to monitor the health, growth, and fecundity of the animals, as well as the effectiveness of the biological filtration. For *Crepidula* snails, feeding can be assessed by the active formation of mucus food strings which are swallowed by the animals [30]. Another way to monitor the effectiveness of feeding is by looking at the quantity and type of fecal pellets produced by the animals. For *Crepidula atrasolea*, direct visualization of fecal material inside the digestive tract (intestines located under the translucent shell) and the accumulation of fecal pellets on the right side of the shell indicates feeding. The color and quantity of these pellets can also be used as an indicator (Pers. Obs., JQH). We have noticed that when animals have less food, this material tends to spend a longer time in the digestive tract. There are also fewer pellets, which may be smaller in size or thinner and ropy, and these tend to be brown or olive in color. When animals receive more food, it tends to spend less time in the digestive tract—the pellets are more numerous, larger, and dark green in color, just like the algal food. These observations suggest that animals digest the available food more thoroughly at lower phytoplankton concentrations, but we have not tested this empirically. In conditions of excess food, poor-quality food, or high levels of inorganic sediment, non-ingested food in the form of pseudofeces collects at the anterior margin of the shell [30–32]. Close observation of feeding in *C. fornicata* shows that the gill is highly efficient at collecting particulate matter, and feeding is continuous via a mucous string formed at the distal ends of the gill filaments [33]. Pseudofeces are produced at a low rate, which includes rejection of the mucous strand [33]. As food concentration increases, the production of pseudofeces increases. Direct quantification of feeding in a related species, *Crepipatella peruviana* (=*Crepidula fecunda*), showed that feeding is continuous and optimal in this species at 1.4 × 10^5^ cells/ml [32]. Above this concentration, rejection rates and the formation of pseudofeces increase dramatically [30, 32]. However, at lower concentrations, very little food is rejected, and continuous feeding appears to be more efficient. While direct particle measurements and gill morphology do not support particle selection capabilities in *C. fornicata* [33–35], there is evidence of selection for size and quality of microalgae in *C. peruviana* [31]. We have observed another indicator of health and nourishment related to the size of the foot, which appears to shrink in malnourished animals. One can also monitor the growth directly by measuring the size of the shells and looking at the deposition of new growth rings. Other investigators have also tracked shell thickness, dry body weight, etc. to track animal health and productivity [36]. In addition, one can monitor the brood sizes produced by mature females. Healthy, well-fed animals tend to be larger, producing more eggs and larger broods [37]. After some trial and error, we have found that 3.0 ml of diluted food (diluted to 75% concentration with sterile dH_2_O, 2.24 × 10^9^ cells/ml), delivered every 2 h, is sufficient to rear snails in this system (using a 45-gal system volume). Direct cell counts measured food in the system at 4.6 × 10^4^ cells/ml shortly after food has been injected into the sump and mixed and 1.5 × 10^4^ cells/ml immediately before food is added, which represents the point of lowest food concentration. Over the course of the day, animals are fed at least 5.5 × 10^5^ cells/ml. In comparison, we and other users feeding in static systems typically feed animals once per day, at much higher concentrations ranging from 3.86 × 10^6^ cells/ml [38] to 1.5 × 10^5^ cells/ml for juveniles [39]. In our previous static feeding arrangement [9], we fed adults 7.5 × 10^6^ cells/ml (10–20 full-size females and assorted smaller animals). Juveniles in small Petri dishes are fed 2 × 10^6^ cells/ml daily prior to transfer to the aquarium system. Feeding in *C. peruviana* has been tested across a range of food concentrations from 1.5 × 10^4^ to 2 × 10^5^ cells/ml [32]. While these experiments were not used to measure growth rates, the highest value had a chlorophyll-a concentration 2.5 times that reported in the natural habitat during spring algal blooms [32]. Time to sex change may also provide an index of animal health, with increased food availability corresponding to increased growth rates and earlier completion of the sexual transition to female [40]. Mérot and Collin [40] tested a range of food concentrations from 2.4 × 10^6^ to 1.5 × 10^8^ cells/ml in *Crepidula* cf. *marginalis* and *Crepidula incurva*, which was delivered over the course of the day to limit potential food rejection at higher concentrations. Food concentration did not affect the size at which animals became female in either species, but low food concentrations increased the length of the transitional period in *C. marginalis*, while there was a delayed onset of sex change with no alteration in the length of the transitional period in *C. incurva* [40]. We are currently measuring the shell deposition rates, time to first brood formation, and brood size for *C. atrasolea* in this system (these data will be presented in a future publication, Lesoway, Perry, and Henry, *In Preparation*).

The amount of food added ensures an ample concentration will be circulating through the system during the feeding cycles. Of course, given the large volume of the system, much of the food is ultimately not consumed, especially when there are fewer animals housed in it. One can maintain a very large number of animals in a single aquatic rack system, and the upper carrying limit is yet to be determined. We currently use one half of the available tank space and estimate the total snail population at 2000 individuals. As the water volume and/or the number of animals increases, more food may be needed to support their growth and reproductive output. As animals grow larger and become more numerous, one has to make corresponding adjustments in terms of the volume of food that is added during feedings. Likewise, given their tremendous reproductive capacity, one will ultimately need to remove some animals from the system to ensure that the carrying capacity is not exceeded.

Note that the stock commercial foods are very concentrated and rather viscous. The high density of the food provided by the manufacturer helps to keep it in suspension, which prolongs its lifespan. However, to make it easier to deliver, the food is diluted to 75% concentration with sterile RODI water (3 parts food to 1 part sterile dH_2_O). The stir plate and Teflon-coated stir bar automatically resuspend the food (once every hour and 5 min prior to feeding, Fig. 5e). With the volume and intervals of food being delivered in our system, a 1-l food reservoir holds enough food to last 28 days. One must be very careful not to contaminate the food. The food and all parts of the feeding system are contained within the small refrigerator (at 4 °C), see Fig. 5a, b, with the exception of the exposed portion of the Teflon feeding line that leads to the sump (Fig. 5f). The air pump helps to purge the line of food, but a small amount of food will remain in the tubing. The feeding line should be kept as short as possible, and elevating the feeding system above the sump allows gravity to help clear the Teflon feeding line. It is advisable to clean and sanitize parts of the feeding system on a regular basis. Possible sources of contamination could include air from the air pump, the exposed open termination of the feeding line located in the sump compartment, and the small amount of the food that sits in the Teflon line at room temperature between each feeding, which could allow bacteria to grow over time. We have so far not found contamination to be an issue. One could install a small HEPA filter on the air pump’s intake, if necessary.

### Keeping track of animals

One challenge with rearing large numbers of animals is to keep track of them. In addition to labeling tanks and tubes (Fig. 1d), one can also label the shells of larger mature animals (females) with small micro-QR or Datamatrix codes. In a future paper, we will describe special tools and a useful relational database we developed to record the system parameters and to follow individual snails and their offspring (Lesoway, Perry, and Henry, *In Preparation*). The modular nature of an aquatic rack system makes it easy to separate animals and their lines.

### Servicing the system

Instructions for servicing the system are included in the SOP (see Additional file 1, SOP). All parts of the system are modular and easily disassembled for cleaning, repairs, or replacement. Only a few basic tools are needed to service the system, which are also described in the SOP (see Figure S1 in Additional file 1, SOP, Section 2: Tools for Servicing the System). These include thin silicone rubber grip pads (similar to those used to open stubborn jars in the kitchen), which are handy for loosening and tightening the PVC unions. Likewise, a small filter wrench (Channellock part no. 209) is useful for stuck PVC unions. A large and a small housing spanner wrenche are needed for loosening and tightening the filter bowls (Pentair parts 150296-75 and 150295-75), respectively. Two 12″ adjustable wrenches are required for loosening and tightening various plastic pipe fittings. A specialized valve adjustment tool is needed for adjusting the tension on the large ball valves. In addition, an IR non-contact thermometer is also useful for monitoring the temperature of the main water pump housing.

Parts of the feeding system need to be cleaned and sanitized using sterile water and 70% ethanol on a regular basis to prevent the growth of harmful bacteria. In addition, one needs to periodically remove any accumulated debris and algae that will grow in the aquatic system. Parts should be cleaned mechanically and rinsed with fresh water only.

Under no circumstances should one use detergents, bleach, or other cleaners to clean parts of the system. These agents are extremely toxic to marine invertebrates. Users should also be sure to cleanse their hands thoroughly with fresh water and avoid using soap. Users should also not use lotion or wear perfume, etc., which can be transferred to the seawater and poison the system.

Some parts of the system are “consumable” and will need regular replacement, including the mechanical filter media, the UV bulb, and possibly the quartz sleeve. This is all described in Additional file 1, SOP. The main circulating pump may also need replacement. Run time and problems associated with cavitation and resulting shockwaves can lead to wear and tear on the motor and pump impeller (sonic sensors could be installed to automatically detect cavitation), which is also affected by the level of dissolved gasses (e.g., oxygen), but this noisy condition is readily detected by the human ear (see Additional file 1, SOP, Section 21: Pump Impeller Cavitation).

### Potential problems

Certain problems are inherent to any marine aquarium system, which are discussed in many useful references, and these will not be described here [5, 6]. No system can be completely hands-free, though this one is near to that point. The system can run for days or weeks at a time, with little or no human intervention. However, one must still monitor the system parameters (which can be accomplished remotely) and water quality, and refill the reservoirs and top off the food, as needed. In an automated system with this level of complication, there is always the possibility of some failures, and someone needs to be on standby in case there is a problem that needs to be addressed. Generally, users will be alerted to most problems via the automated push notifications. While many issues will not turn out to be catastrophic, some failures could lead to the death of the animals. For example, unregulated temperature control, resulting from a sensor error or a bad relay, could lead to low or high temperatures that may be fatal for the animals. Likewise, a problem with the dosing pumps could fail to deliver the proper amount of RODI water, which could lead to dangerously high or low salinity levels. Failures in the feeding system could affect the delivery of food, which could starve the animals or deliver too much food. The latter could lead to the buildup of toxic levels of ammonia, nitrites, or nitrates that could harm the animals. One must be prepared for these and other problems and be ready to correct them. For example, if the main pump fails, one must be ready with a backup to replace it quickly. It is prudent to stock replacement parts for just such emergencies. We try to keep extra filters, plumbing fittings, syringe pump fittings, and other parts on hand, along with the proper tools needed to install them. For most problems, users would ultimately be alerted by text messages and emails to allow enough time to fix them.

It should be noted that this system does not incorporate any sensors to automatically monitor the levels of ammonia, nitrites, or nitrates. Users are required to monitor these on a regular basis using commercial test kits (see Additional file 1, SOP, Section 11: Monitoring Water Quality).

The plastic parts used in this system are very durable and well-made and designed for a long lifespan. For the sump and drain troughs, all plastic seams are glued and thermo-welded. Leaks could form in the system, and most likely, these would occur between threaded parts, such as the PVC unions, various valves, and other parts in the plumbing system that could become loose or crack over time. It is wise to inspect for any small leaks. In the event of a substantial leak, this would eventually be detected by the ultrasonic water level sensor located in the sump, which will shut down the system. Small or large leaks can also be detected by installing various audible water leak detectors on the floor beneath the aquatic rack system.

### Inconsistency of system sensor readings and their calibration

One should never rely only on the system’s internal sensor readings. Some sensor readings will drift over time and need periodic recalibration. This is very important, as incorrect readings will lead to inappropriate adjustments of various water quality parameters (e.g., conductivity, temperature, and pH). The temperature sensors seem to be reliable and fairly consistent, and the system maintains conductivity within ± 2 mS, but measurements obtained for conductivity and pH tend to drift over time. Extreme changes can be lethal. These sensor readings need to be verified and sensors re-calibrated regularly. We generally check water quality parameters (e.g., pH, salinity, nitrogen, Ca^++^, KH) on a weekly basis (see Additional file 1, SOP, Section 11: Monitoring Water Quality). Based on our own external measurements, we calibrate the pH and conductivity sensors every 3–4 months. To do this, one must have fresh calibration solutions, as well as an independent set of instruments to check the temperature, pH, and conductivity (see Additional file 1, SOP, Section 15: Servicing the Sensors).

Other issues can affect sensor readings. For instance, the temperature will affect both the conductivity and the pH readings. In general, the temperature sensors, float switch, and paddle wheel flow meter are fairly dependable, though it is possible for the float and paddle wheel to get stuck. In addition, readings for the sensors in the sensor loop will fluctuate dramatically if the water flow changes or is shut off. Both the pH and conductivity probe have built-in temperature probes for self-compensation. However, once the flow of water is interrupted to the sensor loop, the temperatures will begin to change. This will impact the pH and conductivity readings, but those probes also rely on running water to make accurate measurements. An independent set of instruments is needed to verify sensor readings. We use a standard glass lab thermometer or a certified digital thermometer to verify the water temperature (see Additional file 1, SOP, Section 16: Double Checking the Sensors). Salinity can be determined by one of several methods. One method is to measure the specific gravity. This can be accomplished using a hydrometer or a refractometer. A calibrated plastic or glass hydrometer is not as useful, as one has to remove water to make the measurements, and a floating hydrometer needs to be used in a rather deep container like a graduated cylinder. A refractometer is very useful as only one drop of seawater is needed for each measurement. These can be obtained fairly cheaply. One should be aware, however, that the density of water will change with temperature. These instruments are calibrated to work within a certain range of temperatures or may have some built-in temperature compensation. Likewise, a portable conductivity sensor is convenient to take direct measurements within the aquatic rack system. Conductivity meters need to be calibrated for each use with a standard calibration solution of known conductivity. Unfortunately, these solutions have a limited lifespan. We use an inexpensive meter made by Hanna (see Figure S1 in Additional file 1, SOP, Section 16: Double Checking the Sensors), which has built-in temperature compensation and can be set to read in different units, such as millisiemens (1 mS is the reciprocal of 1 ohm), ppt, or units of specific gravity (note that pure water has a specific gravity of 1.000). Again, one should note that conductivity and density change with temperature (e.g., the density of seawater (about 35 mS) changes from 1.028 g/cm^3^ at 3.98 °C to 1.025 g/cm^3^ at 20 °C to 1.023 g/cm^3^ at a temperature of 80 °F; 27 °C = 80.6 °F). Likewise, we use a simple digital pH meter to validate the pH readings (see Figure S1 in Additional file 1, SOP, Section 16: Double Checking the Sensors).

### Room for improvements

While this system works very well and has reduced our labor significantly, there is still room for improvement. As discussed above, some of the main issues we have encountered are related to evaporation and salt creep. In the future, better seals on various lids, etc., would limit these issues. Tight tolerances for better fitting lids, and the use of silicone gaskets, etc., could make this possible. The system could also be designed with shallower drain troughs, placing the ends of the tank drain tubes closer to the bottoms of these troughs. This would help reduce splashing and salt creep.

The system takes up a fair amount of floor space (Fig. 1a). It might be possible to reduce this footprint by integrating some of these components within the aquatic rack system itself (perhaps by installing these on the right side or on the bottom deck of the rack).

Presently, the aquatic rack system and the feeding system have separate microcontrollers, with their own real-time clocks, and those should be carefully synchronized so that all events are carefully coordinated. Though the microcontrollers communicate with one another, a more complete integration of these systems, perhaps using a single microcontroller or at least a single shared clock, would be preferable. Likewise, the ATK automatic seawater top off system is a separate unit, and its activity could also be tied to those of the other systems.

Currently, we rely on fluorescent room lighting, which is on a timer for 12 h of “daylight” (7 am to 7 pm) followed by 12 h of darkness. One could incorporate built-in LED lighting and provide the necessary wavelengths, depending on what animals are being reared in the system (e.g., corals). Algal growth is unavoidable in marine aquaria. Though algal growth is not necessarily a problem, one should try to limit it. Some parts definitely benefit from blocking out the light in areas that are prone to algal growth, or in areas that should be free of algal growth, such as in the sump and the canister filters. Dark covers should be fashioned for the filter bowls, as described above (Fig. 3h). Periodically, tanks may need to be cleaned to remove excessive algal growth. In our case, the freshly hatched juvenile snails are positively phototactic, and some tend to climb above the waterline. These snails eventually perish through desiccation and the lack of food. Having light directed from below or the inclusion of darker lids on the tanks could help increase the survival of the snails.

The protein skimmer plays a key role in removing organic material and uneaten algae. This aquatic system could benefit from a more efficient/larger protein skimmer; however, the sump would have to be redesigned to accommodate such a skimmer.

For more precise regulation of the temperature, it would be best to integrate the chiller directly into the aquatic rack system, as mentioned above, and to use the Walchem 900 controller to regulate both the heater and chiller. The external chiller does have some internal protection against freezing, which would otherwise block water flow, but additional protection using the aquatic rack system’s flow sensor could be implemented to disconnect power to the chiller should the water flow be interrupted. This would help mitigate potential problems with freezing water. There is an additional unused solid state relay (“R8”) available in the Walchem 900 controller that could be used for this purpose with some additional circuitry (see Additional file 1, SOP, Section 14: External Chiller System).

## Conclusions

For the first time, we describe an automated aquatic system for laboratory culture of filter-feeding marine invertebrates. While our goal was to build a system to rear *Crepidula* snails for laboratory research, the equipment and aquaculture techniques should be of broad interest for laboratory cultures of other marine organisms, such as bivalves, crustaceans, sponges, polychaetes (e.g., feather duster worms), and corals. This system takes care of monitoring and adjusting essential water quality parameters and automatically feeds the animals. The total cost of this system was around $32,000 USD, which included freight, installation, training, and the chiller and all other parts needed to fabricate the feeding system. Some components may be unnecessary, and removing them would reduce the overall expense (such as the UV light, sump mechanical float valve, and pH dosing pump). While the cost of this system may seem high, it is not exorbitant when one considers the annual, recurring cost of labor needed to maintain standard aquarium systems to rear larger numbers of animals. For a new faculty member just starting their lab, this expense is rather small compared to other pieces of lab equipment. The required husbandry to maintain good animal health is fundamental to research, but this can be time-consuming. Recognizing that no aquarium system is truly hands-off, this system significantly reduces time spent on animal care, increasing researcher availability for more productive pursuits inside and outside the laboratory.

## Supplementary information

**Additional file 1.** Standard Operating Procedures (“SOP”), which describes how to operate and maintain the aquatic rack system. This file includes Figure S1-S4, and their legends.

**Additional file 2.** List of parts and suppliers for components used in the aquatic rack system. Note: Additional files 3, 4, 5, 6, 7, 8, 9, 10, 11, 12, 13, 14, 15, 16 are available at Github [41].

**Additional file 3: **Construction diagrams for fabricating various components used in the aquatic system. Dimensions are indicated in red and hidden holes and recesses are showing in green for various projections. **A**. The aluminum foot pads to help distribute the weight of the system on the floor. Four of these pads are required. The rounded recesses receive the stainless steel adjustable feet. **B-C.** The PVC covers for the drain troughs to reduce evaporation and splashing are machined from “¼”: thick grey plastic PVC. Two styles accommodate **B**) the drain trough for the six larger 10 liter tanks located on the bottom shelf, and **C**) the drain troughs serving twelve 3 liter tanks situated on each of the five upper shelves. For ease of installation, each of these covers consists of two pieces, as shown. **D**. The polycarbonate “Origami” cover for the right sump compartment is cut form a single sheet of “1/8” thick clear polycarbonate. Dashed lines indicate folds. All dimensions are indicated relative to the zero (0,0) reference point shown. **E.** Custom machined plumbing connections to adapt the Artica chiller to “3/8” right angle polypropylene compression fittings for the tubing that serves the sensor side loop. Two of these fitting are needed for influent and effluent sea water. These parts are used with the rubber seals provided by Artica for the original fittings. **F-H).** The adjustable aluminum metal clamp system used for the capacitance food reservoir, level sensor consists of three parts: **F**) the threaded post, **G**) the adjustable clamping block and **H**) the adjustable L-shaped bracket that holds the sensor. Machine screws (not shown) are needed to assemble these last three parts [41].

**Additional file 4.** Fritzing diagram (“Feeding_controller2.ino” circuit diagram) for wiring the feeding system microcontroller circuits, along with its specific connections to various peripheral devices (e.g., the AC power relays, peristaltic pump controller, capacitance food level sensor for the reservoir, and IR feeding line sensor), as well as the Walchem 900 controller. See Additional file 2 for the list of parts and suppliers [41].

**Additional file 5.** Arduino Uno sketch (“Aquarium_feeding_controller.ino” firmware program) needed to run the feeding system microcontroller [41].

**Additional file 6.** 3D printer file (“LCD_Bezel_98.stl”) for generating the plastic bezel for the LCD displays. This part is held in place using cyanoacrylic glue [41].

**Additional file 7.** 3D printer file (“Articulated_arm_base_plate_1.stl”) for generating the plastic base plate for the articulated arm that supports the feeding system’s microcontroller housing (part 1). This part is fastened to the microcontroller using industrial grade, double stick foam tape [41].

**Additional file 8.** 3D printer file (“Articulated_arm_base_plate_2.stl”) for generating the plastic base plate for the Articulated arm that supports the feeding system’s microcontroller housing (part 2). Two machine screws are needed to fasten this part to the unused door hinge mounting holes located at the top of the mini-refrigerator [41].

**Additional file 9.** 3D printer file (“tubing_holder_base.stl”) for generating the base of the plastic food line clamp for the IR feeding sensor (part 1) [41].

**Additional file 10.** 3D printer file (“Tubing_holder_top.stl”) for generating the hinged top of the plastic food line clamp for the IR feeding sensor, which snaps onto the base (part 2, see Additional file 9) [41].

**Additional file 11.** 3D printer file (“IR_LED_retaining_strap.stl”) for generating the plastic retaining strap for holding the IR LED in the IR feeding sensor (see Additional file 9). Two small machine screws needed to fasten these parts are not shown [41].

**Additional file 12.** 3D printer file (“IR_sensor_retaining_strap.stl”) for generating the plastic retaining strap for holding the IR sensor in the IR feeding sensor (see Additional file 9). Two small machine screws needed to fasten these parts are not shown [41].

**Additional file 13.** Fritzing diagram (“Peristaltic_pump_controller.fzz” circuit diagram) for wiring the programmable peristaltic pump controller [41]. See Additional file 2 for the list of parts and suppliers.

**Additional file 14.** Arduino Uno sketch (“Stepper_motor_controller.ino” firmware program) needed to run the peristaltic pump controller [41].

**Additional file 15.** Fritzing diagram (“DC_motor_controller_interface2.fzz” circuit diagram) for constructing the protein skimmer ATtiny85 control interface [41]. See Additional file 2 for the list of parts and suppliers.

**Additional file 16.** Firmware program (“ATtiny_motor_controller.ino”) for the protein skimmer control interface’s ATtiny85 microcontroller, along with specific connections to the Tunze 9004 DC motor control board and the Walchem 900 controller [41].

**Additional file 17.** 3D printer file (“Skimmer_control_box_housing.stl”) for generating the plastic housing for the protein skimmer control circuit (part 1). Two holes must be drilled into the sides to accommodate rubber grommets for the electrical cords [41].

**Additional file 18.** 3D printer file (“Skimmer_control_box_lid.stl”:) for generating the plastic lid for the protein skimmer control circuit’s housing (part 2, see Additional file 17) [41]. Once assembled, this lid is held in place using silicone cement.

## Data Availability

The relevant construction files generated during the current study are available on GitHub [41]. All other relevant information is included in this article and its supplementary information files.

## Abbreviations

ATO: Automatic Top Off
HEPA: High-Efficiency Particulate Air
lpm: liters per minute
PVC: Polyvinyl Chloride
RODI: Reverse osmosis deionized
SG: Specific gravity
SOP: Standard Operating Procedure
UV: Ultraviolet
