# Supplementary material for: An automated aquatic rack system for rearing marine invertebrates
Source: BMC Biol. 2020 May 4;18:46. doi: 10.1186/s12915-020-00772-w (PMC7199361; doi:10.1186/s12915-020-00772-w)
Supplement: Supplementary file 1 — Additional file 1. Standard Operating Procedures (“SOP”), which describes how to operate and maintain the aquatic rack system. This file includes Figure S1-S4, and their legends. [file 12915_2020_772_MOESM1_ESM.pdf]

Marine Aquatic Rack System Standard Operating Procedure (SOP): (28 February 2020) ** Version 20. Henry Lab, UIUC

Jonathan J. Henry, Maryna P. Lesoway, and Kimberly J. Perry

Department of Cell & Developmental Biology
University of Illinois
601 South Goodwin Ave.
Urbana, IL 61801

Note, please see the accompanying manuscript (Henry et al., 2020) and Additional File 2, "List of Parts and Suppliers" for more details regarding this system

This system was designed in collaboration with Iwaki Aquatics, Inc., and the automated feeding system was designed by Dr. Jonathan Henry (UIUC), which was funded by an NSF EDGE GRANT (1827533)

Supplemental Figures are included here (called Figures S1-S4). Figure notations (to Figures 1-5) refer to those included in Henry et al. (2020). Other figure references include the corresponding Additional Files that accompany Henry et al. (2020), see also **https://github.com/HenryLabUIUC*.***

1. Basic Control of the Aquatic System (Walchem Controller and Power Switches):
The main system is controlled by a Walchem 900 controller (model number WEL series 191653-20, Iwaki Aquatics, Inc., Holliston, MA; **Figure 4A-B**). Two electrical switches control power to the main system. A main power toggle switch (located on the bottom right of the Walchem 900 controller) will disconnect all power to the system. Note that system (e.g., main pump) power status is indicated by Alarm light A1 on the controller. A second, rotary switch (to digital input D1, on the bottom left) places the system in **standby** mode. In **standby** mode the main water pump, UV lamp, and skimmer will be turned off. In addition, the system's electromechanical, rotary bypass valve will return to filtering mode. In **standby** mode all sensors will continue to operate, and the system display panel will continue to be active. Alarms may still be triggered, depending on the sensor readings. For many servicing needs, one can use the rotary **standby** switch to place the system in **standby** mode. When servicing any electrical components the system must be shut **off** using the main power switch and unplugged. A dedicated, 20 amp 120VAC electrical circuit is required for the operation of this system. Additional electrical circuits are needed to power other devices, such as the protein skimmer and feeding system.

Note, for specific instructions related to the use and programming of the Walchem 900 controller, readers are referred to the manufacturer's instruction manual:
https://www.walchem.com/Literature/Controllers/W900/180686_W900%20Manual_E.pdf

Some words of caution: users must be careful when accessing the system's Walchem 900 controller to make any changes. All inputs are accepted immediately once they are entered, including any accidental changes (and no confirmation is requested when inputting changes). Any unintended changes could have a profound effect on system operation and animal welfare. At the end of this document, we include a complete list of settings used for our system.

2. Tools for Servicing the System (Figure S1):
Certain basic tools are needed to service the system (**Figure S1**). These include thin silicone rubber grip pads (similar to those used to open stubborn jars in the kitchen), which are handy for loosening and tightening the PVC unions. Likewise, a small filter wrench (Channellock, Inc. part no. 209, Meadville, PA) is useful for stuck PVC unions. Large and a small filter housing spanner wrenches are needed for loosening and tightening the filter bowls (Pentair parts 150296-75 and 150295-75, Pentair Aquatic Ecosystems, Apopka, FL). Two 12" standard adjustable wrenches are required for loosening and tightening various plastic pipe fittings. Basic straight and Phillips head screwdrivers for loosening and tightening clamps, etc. A specialized valve adjustment tool is needed for adjusting the tension on the large ball valves (provided by Iwaki Aquatics, Inc., Holliston, MA). In addition, an IR non-contact thermometer (e.g., EXTECH IR250, FLIR Commercial Systems, Inc., Nashua, NH) is also useful for monitoring the temperature of the main water pump housing. Handheld pH and conductivity sensors are also needed to asses water quality. The latter generally also incorporate temperature sensors as well for internal compensation. Spare fuses are needed in case the system fuse blows. Silicone grease is needed to lubricate the filter cartridge bowl O-rings. A roll of plumber's Teflon tape is needed for sealing certain plumbing fittings. In addition, a long-handled ice scraper is useful for removing salt deposits from the system, especially those forming in less accessible areas.

3. The Rack Shelving Unit (Figure 1A):

The aquarium system, including the rack shelving unit was constructed by Iwaki Aquatics, Inc. (Holliston, MA). The rack is made of welded and powder-coated/painted aluminum and holds up well to salt water. Four small adjustable stainless steel feet serve to level and support the rack. As the system is very heavy once it is filled (approximately 1100 lbs/ 550 KG at capacity), these feet could eventually cause impressions in the flooring. As a precaution, and to distribute this weight, we machined a set of four ½ inch thick, 4" x 4" aluminum pads with central recesses for the leveling feet (See **Figure 1A, C**, and **Additional File 3A**). The typical Iwaki rack can be constructed with either five or six shelves depending on the configuration of tanks, and has a foot print of approximately 5'-6" wide x 1'-4" deep). Smaller racks with fewer shelves can also be made to order. Tall units need to be anchored either to the wall or to the ceiling to prevent them from accidentally tipping over in the event of an earthquake or if someone were to lean/pull on the unit (**Figure 1A, C**). There is little that needs to be done to service the shelving unit itself, other than to periodically clean it with absorbent towels and fresh water to remove spilled sea water and salts.

The main rack occupies approximately 11 sq. ft (5'-6" wide x 2" deep), which includes some space between the back side of the rack and the wall, and it is 7'-8" feet tall. For access and cleaning the rack has been placed 10 inches away from the wall. The various reservoirs, feeding system and the chiller are all external components that take up additional floor space. The feeding system (**Figure 5A**) occupies 3 sq. feet (1'-6" wide by 2 feet deep) and the 5 gallon sea water reservoir (a plastic bucket, see **Figure 4H**) requires another 1 sq. foot of space (approx. 12 inches in diameter). The external chiller occupies 1.5 sq. feet (1' wide x 1'-6" deep). The two dosing tanks (external reservoirs) for the RODI water and a mix of calcium hydroxide, and calcium carbonate take up 1.5 and 3 sq. ft (15"x 15" and 15-½" x 21-½", respectively, see **Figure 4E**).

4. Water Flow (Figure 2):

4.1. Water Flow During Normal Operation:

An exploded diagram has been provided to show the main parts of the aquatic system and the various routes of fluid flow (**Figure 2**). Note that all valves and other key components are referenced using specific numbers and initials, which we have marked with adhesive plastic labels. Relays (R#), digital inputs (D#) and alarms (A#) are numbered, accordingly, as indicated in this document. Likewise, valves are numbered for convenience, but in no particular order. Except for the needle valve #6, ball valves rotate a total of only 90° (Handle positions on ball valves relative to the long axis of the PVC pipes indicate their operation: the parallel position "|" is open; the perpendicular position "T" is closed, see examples in **Figures 1L, 4N-O, Figure S2**). The ball valves typically rotate clockwise to close them and counterclockwise to open them. These directions are typically marked with arrows on the larger valve handles. Stiff, loose or leaky valves can be adjusted with a special valve adjustment tool (see below).
During **normal** operation the valves should be in the following positions:

1. Main pump motor inlet ball valve #4 should be completely open (**Figures 1L, 2; Figure S2A**)

2. Main pump outlet ball valve #1 should be completely open (**Figure 2; Figure S2B**)
3. Sensor loop ball valve #2 should be either completely open or ½ open (45° position; **Figure 2; Figure S2B**)

(Note valve #2 must be completely open when using the external chiller, if it is attached to the sensor side loop.)

4. Needle valve #6 should be opened just enough to keep side bio-reactor filter loop pellets moving (**Figures 2, 3L; Figure S2C**). Turning this valve clockwise will close it. Never over-tighten this valve to completely close it, as that may damage the needle.
5. Main bypass ball valve #5 should be completely closed, unless more bypass water is required to return to the sump to prevent cavitation or to limit water flow to the tanks (**Figures 2, 4N-O; Figure S2D**, see below).
6. Ball Valve #7 ("AT") should be completely closed (**Figure 2; Figure S2E**; this is the Accessory Tap Filler valve for the attached blue hose, see below).
7. Spring loaded "quick connect" drain valve #3 should be closed (**Figures 2, 3L**; **Figure S2C,** e.g., with nothing attached!) This is the main drain valve. This male valve automatically opens when the female connector is attached, see below.
8. Individual ball valves to each row of tanks (rows lettered A-F, from top to bottom) should be adjusted as needed: typically, ½ open (45° position). If there are no tanks on these rows they should be closed (**Figure 2; Figure S2F**).
9. Individual tank ball valves should be opened as needed (**Figures 1D, 2; Figure S2G**). Typically, these should be completely closed if there are no tanks or open fully or less, as needed. Typically, these are set to ½ open (45°) for the 3 l tanks and both are set completely open for each of the 10 l tanks.

4.2. Water Flow During Feeding
(Electromechanical bypass valve, input from R3, regulated by D5; Figures 2, 5I; Figure S2H):

Note that during feeding the filter loops (including the bioreactor side loop; **Figure 2 and 3K**) will be bypassed so that food can freely recirculate between the sump and the tanks. This is achieved by a motorized ball valve (**Figures 2, 5I; Figure S2H**). The bypass valve is regulated by relay R3. When the relay is open, the system is in filtering mode. When the relay is closed, the system is in feeding mode. Normally, the state of relay R3 is controlled by a digital input from the feeding system (D5). A small window on the bypass valve "BP" indicates its current status (**Figure S2H**). If the window is black, the unit is in filtering mode. If the window is white (side of stem colored with white paint), the unit is in feeding mode. When the valve is in the feeding position, Alarm light A3 will be illuminated. During the feeding mode, sea water will not pass through the filters due to the presence of a reverse flow check valve (**Figures 2, 3L**). Water flow will thus encounter less resistance due to the removal of the filters and no water will return to the sump via the small bioreactor filter loop. Therefore, during feedings the effluent, outlet pressure indicated on the round gauge will rise approximately 3psi (e.g., with a new pleated filter from approx. 10-13 PSI to 13-16 PSI; **Figure 2; Figure S2B**). The flow rate to the tanks will also increase somewhat during feeding from by approximately 3 lpm (e.g., with a new pleated filter from 29-30 l/min to about 32-33 l/min, or possibly as high as 34l/min). (Note that the level of water in the sump will be lowered by about 0.5 to 1 cm during feeding). The noise level of

water flowing through the main pump will generally increase during feeding. This is normal, but one should pay attention for any signs of cavitation (see below). The only bypass water that will return to the sump during feeding will come back via the sensor loop, unless the main bypass loop (valve #5, **Figures 2, 4N-O; Figure S2D**) has been opened somewhat. Note that depending on the flow rate, some bypass water must return back to the sump or cavitation may occur (see below). The UV lamp will continue to operate during feeding, as it requires flowing sea water to cool it (otherwise the PVC housing would melt!; **Figure 3I**). During feeding the skimmer is also turned off (**Figures 3C-F, 5G-H**). When the filters are turned back on after feeding, there may be a slight delay in the formation of new skimmate by the protein skimmer (though we have not yet encountered any such problems). There is normally no need to make any system adjustments during feeding.

Note that as the filters become clogged, the effluent outlet water pressure will drop (as will the overall flow rate). Pressure on the intake side of the filters will increase while that on the outflow side will decrease (the latter being the pressure read by the small round gauge; **Figure 2; Figure S2B**). This gauge can be used as an indicator for potential problems or when it is time to replace the pleated 50μm filter (**Figure 3G; Figure S2B**), though the reading is somewhat misleading. For instance, if one opens up the valves supplying the small tanks this pressure reading will drop and the flow rate will increase. Typically, filter replacement is required when the inlet and outlet pressure difference is 10psi. Once the pressure difference gets above that, there is a small risk that the filter cartridge can collapse/crush. In our system there is no second gauge that reads inlet water pressure to the filters, so the pressure difference cannot be determined. Most labs care more about the flow rate to the tanks than the pressure in the filters. There is a direct relationship between flow and pressure with pumps, so it's easier to just change the filter according to changes in flow rate. As the filters become clogged you will also notice that the noise level of the system will become quieter, as less splashing water returns to the sump. It is recommended to change the filter once the system's filtered flow rate drops to no lower than 20 lpm, but one can change the filter before that point. One can gain some additional time before needing to perform a filter change by simply opening up the valves that supply sea water to the manifolds for each row of tanks (valves A-F). This will also impact the flow occurring during feedings, which will also be elevated (one should limit the maximum flow during feeding to around 35-36 lpm). At some point though, the effluent water pressure will drop to low and the float switch will not rise, triggering a no-flow alarm. At that point the filter must be changed immediately, as this will begin to impact the reading of temperature, conductivity and pH, which may lead to lethal changes, as the system tries to compensate for the false readings! While in some other applications it may be possible to clean clogged filters, in our case the biofilm, finer debris and algae trapped by the filter material are much harder to remove simply by washing the filter, and, thus, the filters must be replaced. Furthermore, the material that is trapped by the filter will continue to break down within the system, so the longer the waste/food stays in the system, the more organics/nitrogenous waste will accumulate in the water. Filtering flow rates with new filters are around 29-30 lpm, and effluent pressures should return to around 10-12psi (32-34 LPM and 13-15psi during feeding). We have observed approximately two—three months of service life for each pleated filter.

5. Individual Tanks (Figures 1D, 2):

The polycarbonate tanks are manufactured by Pentair Aquatic Ecosystems (Apopka, FL; **Figure 1D-G**). These tanks accept slotted or nylon mesh "larval" baffles (**Figure 1F**). The larval baffles are available in three mesh sizes (400, 700 and 1000µm). These baffles slide into molded channels in the back of each tank and are fairly secure. Their purpose is to prevent animals from reaching the rear drain tube where they could travel to other parts of the system. In this system we use baffles with 400µm openings, which are small enough to prevent *Crepidula atrasolea* embryos or juvenile snails from escaping the tanks. Each tank is equipped with a plastic lid (**Figure 1E**). Unused holes in these lids are plugged with small white plastic snap caps to help limit evaporation and water spray (**Figure 1G**; McMaster Carr, part number 9688K25162PP050BG14, Elmhurst, IL). These plugs can be further sealed using silicone aquarium cement (**Figure S2G**).

1. NOTE: When servicing a tank, first shut off the tank's own valve(s)! Then remove the white tubing from the lid access hole(s) before removing the lid or the tank. Be aware that there may be a lot of moisture (typically fresh water condensate) under the lids. When removing a lid, be careful to drain this condensation back into the tank to prevent a mess.
2. If the tank is to be removed, tilt the tank backwards to drain off some sea water first. Remove the tank carefully so as not to spill the sea water. Lift the tanks straight up to clear the drain tube from the drain cover (**Figure 1H-J**), and the ridges on the bottom of the tank, which straddle the rack cross bars (**Figure 1E, G**). When done, reinstall tank and/or lid in reverse order, insert the tubing and then turn the tank valve(s) back on. It's easy to forget this last step. Remember to seat the tanks properly, as they have protruding plastic ridges on the bottom that must seat properly onto the metal rack cross bars (**Figure 1A-D**).

One can operate the system with any number of tanks in service. However, when taking tanks on or off line, they should be added or removed with sea water in them. Otherwise, the sea water level in the sump will change.

The mesh on the larval baffles will eventually accumulate a biofilm and waste products, which will impede the flow of drain water. This could lead to an overflowing of sea water from the tank, which could eventually drain the sump. These larval baffles must be cleaned periodically, and it is best to do this on a predefined schedule (every month or two).

Note that if the flow of water through any of the small valves that serve individual tanks becomes reduced, this may be an indication that they have become clogged. Opening and closing the valve may correct the problem, or one can try to unclog these valves by temporarily shutting off all the rest of the small valves that serve other tanks on that particular shelf. The greater force of the higher water pressure will usually dislodge any debris and restore normal water flow thru the clogged valve. If this does not correct the problem, it will be necessary to remove (unscrew) the valve from the manifold to manually clean or replace it.

6. RO and pH Reservoirs (Figures 1A, 2, 4E):

(pH pump currently not in use)

Two plastic reservoirs with lids hold RO (RODI) water and a concentrated salt solution, respectively (**Figures 1A, 2, 4E**). Be sure to periodically check the levels and refill the RO (dH_2O) water and pH salt reservoirs, as needed. Make sure the reservoir lids are correctly installed and the filler intake/vent tubes are in place! The filler tube has a cylindrical weight to keep it submerged. For the pH reservoir one can mix dH_2O with Na-bicarbonate (dissolved to about one half of its saturation point, or 40gm/liter) and Kalkwasser (Ca-hydroxide, dissolved to about half its saturation point, Bulk Reef Supply, Golden Valley, MN). Note, however, that we have not been using the pH pump, and have inactivated this by placing it in HOA off mode (see additional note, below). Rather, we adjust pH manually, as needed. On rare occasions, during the initial establishment of this system, the pH dropped by 0.1 unit. To correct this we added 5gm of Marine Buffer (Seachem, Madison, GA) per 40 gallons of sea water. This elevated the pH by 0.1 unit. More recently, we saw a slight elevation of pH. To correct this issue, we performed a series of partial water changes and began to supplement ionic calcium (see **Section 11: Monitoring Water Quality**, below).

7. Adjustment and Priming of the RO (input from R4) and pH (input from R5) Pumps (Figures 2, 4F):

RO and pH pump operation are indicated by Alarm lights A4 and A5, respectively. Normally, these small red and blue electromagnetic relay pumps, mounted on the back wall to the right side of our rack system, make a distinct rhythmic sound during pumping (**Figure 4F**). When the pumps are running dry and pumping air they will make a much louder popping sound. This is not good for the pumps and is caused by fluid level in the reservoirs being located below the weighted inlets of the supply/vent tubing, or the pump(s) may need to be re-primed. Priming is accomplished by opening the small round plastic valves on the very top of the pumps (**Figure 4F,** see the manufacturer’s instruction manual). Make sure there is enough liquid in the reservoirs. Allow the pumps to run by placing them in HOA “On” mode using the main system control panel. The pumps are primed when fluid starts to exit the vent tubes that hang back down into the reservoirs. At this point the small valves should be closed to allow the fluid to pump to the sump instead. Be sure to verify that this is working! After the problem is corrected return the pump control to HOA “Auto” mode using the main system control panel. RO pumping rate is set to 40 pulses per min. This must be set on the front panel of the RO and pH pumps themselves (**Figure 4F**; follow the manufacturer’s instructions, Iwaki Co. Ltd., Holliston, MA). Adjustments can only be made in the upward direction, so if you miss your desired setting you must go all the way up and back to zero to reset the proper value. These values are typically set to be “locked” on the RO and pH pump control faces so that one cannot accidentally change these (users are referred to the pump manufacturer’s manual for full operation). Pumping rates may be changed, if needed. For example, if the restored levels tend overshoot those desired, then the pumping rate can be lowered (e.g., conductivity overshoots and goes too low, triggering an alarm, during RO water pumping). With lower pumping rates the pump will tend to run longer but the water will have a better chance of mixing so values will be able to stabilize , which helps limit false readings made by the sensors.

So far, we have not seen any need for the pH dosing pump. pH levels appear to be holding fairly steady; though earlier we had noticed these values slowly drop once we added animals and began to feed them, and more recently (after eight months of operation) we saw a slight elevation in pH (see **Section 11: Monitoring Water Quality). We are considering a change to the use the pH pump to deliver periodic, scheduled doses of Ca++ to the system to enhance shell growth.

8. Servicing the Sump Pad Filter (Figures 2, 3A):

The sump has a coarse, fibrous polyester filter pad that sits on a perforated sieve plate (**Figure 3A**). Dirty sump pad filters may be carefully removed allowing any sea water to drain back into the sump and replaced with a fresh filter, as needed. To accomplish this, one must remove the Tent or Origami cover (**Figure 3B; Figure S3A-C**). It is not necessary to remove the slotted PVC drain diverter or to shut the system down to change the filter pad. Change the filter when it becomes discolored and sea water starts to spill over the edges rather than pass thru the filter material. These disposable filter pads should last a few months.

9. Servicing the Protein Skimmer (regulated by D5, input from R7, Figure 3C):

The sump contains a compact Tunze protein skimmer (Tunze Comline DOC Skimmer, 9004 DC, Penzberg, Germany) that fits inside the left compartment of the sump on the far left side (**Figures 2, 3C-F**). Protein skimmer status is indicated by Alarm light A7. It is illuminated whenever the skimmer is turned off, which occurs during a feeding cycle. The protein skimmer is located in the sump and runs automatically when the filters are active, but is turned off during feeding via a custom microcontroller circuit (**Figure 5G-H**; see **Additional File 15, Additional File 16, Additional File 17, and Additional File 18**). Normally, a low level output (0) from pin 3 of the Tunze motor controller's ATMEL 1724 microcontroller turns on the 12 volt DC motor, and a high level output (1) stops the motor. This is the output that is being intercepted by the accessory microcontroller interface, under control of a signal sent by the Walchem 900 controller, when it receives the over-riding command from the feeding station's microcontroller. In other words, when it is time for feeding, the feeding station sends a signal to the Walchem 900 controller to activate the bypass valve (see below). The Walchem 900 controller then sends a signal to the accessory skimmer control circuit which, in turn, interrupts the output of the DC motor controller's own microcontroller to turn off the skimmer. Note that the blue skimmer control power light (located inside the skimmer control box knob) will stay illuminated even during feeding (**Figure 5G**). One can override skimmer function and turn off the skimmer at any time for a preset period (approx. 10 mins) by pushing the small button on the small skimmer control box (**Figure 5G**, the blue light will then go off). The skimmer can be turned back on by pushing the button again or it will automatically turn on after the preset time interval. The speed of the skimmer DC motor is regulated by the illuminated knob on the control box (**Figure 5G**). Typically, this only needs to be set to the 30-50% position, as needed. If this is adjusted too high, excess sea water will enter the skimmer cup. If this is set too low, no skimmate will be formed. This needs to be adjusted empirically. Skimmate needs to be removed from the collection cup (**Figure 3D-G**), and its rate of formation depends on several factors, including the size and amount of bubbles and the concentration of dissolved organics. As supplied, the removable Tunze skimmer collecting cup does not have a drain tube. We have

installed a silicone drain tube that exits the sump on the left side through a bulkhead compression fitting located just above the high water line on the far left side of the sump (**Figure 3D, F**; silicone tubing: 0.188" ID x 0.375" OD, Dow Corning, Midland, MI). Be careful with the skimmer's silicone drain tube. If it becomes disconnected from the small collecting cup the skimmate will simply flow back into the sump. If it gets kinked and collapses inside the sump bulkhead connector, a leak may form. This tubing can be fed to an external tray to collect the skimmate (**Figure 3G**). Note, however, that sea water can enter the collecting cup, as described above (and also see note below), and this would drain out of the tubing to the bucket. A great deal of water could flow through this tubing, which is located just at or slightly below the high-water level in the sump! This could create a huge mess, though we have never encountered such a problem. Alternatively, the open end of the tubing can be elevated well above the water level in the sump and lowered to drain the skimmer cup, as needed. Periodically (once every week or so) one should wipe clean the inside of the skimmer collection cup, its airlift (chimney) and the lid, which will accumulate a great deal of algae and other proteins. More skimmate will form if one keeps the chimney clean (**Figure 3E**). The skimmer is normally controlled by relay (R7). When relay R7 output is low/off (0) the skimmer will run and when relay R7 is high/on (1) the skimmer will stop.

Note that the skimmer is held in place by two strong rare earth magnets (**Figure 3D)** enclosed within the blue and black plastic disks located on the outside, left side of the sump. Do not remove these magnets or attempt to reposition the skimmer. (Also, never put the two magnets together as you will not be able to separate them without tremendous difficulty!). The skimmer sits on top of a perforated platform inside the sump. The platform has four leveling, height adjustable legs. These are set so that the slotted openings in the skimmer cup are located just along the sump high water level (**Figure 3C**). Sea water must be able to enter these slots. The relative water level in the sump is critical for proper function of the skimmer. It should be about halfway up between the top and bottom edges of the intake slots located within the skimmer cup. If the water level is too low, or the skimmer is placed too high, no skimmate will form. If the water level is too high, or the skimmer is placed to low, the skimmer will pump excess water into the collection cup and its drain tube. Precise sump water level is maintained by the RODI pump and the ATO units (see **Section 6: RO and pH Reservoirs**, above, and **Section 33: Automatic sea water Top-Off system (ATO)**, below). Note that skimmer activity and the Walchem 900 front panel alarm light A7 are regulated by the input from the feeding system (to D5).

Note that the fine bubbles produced by the protein skimmer can also produce jet drops and a fine mist that collects under the lid and this moisture drips into the collection cup. This can fill the cup prematurely, and removes some "clean" system sea water. Careful adjustment of the DC motor's speed can help limit this mist and any lost sea water. Approximately 25-100 ml of liquid waste will be collected by the skimmer each day.

Note also that if you perform a partial sea water change, you may see some excess water being ejected into the skimmer cup, which may flood the collecting tray. This can continue for a couple of days, and it is best to monitor this. It is unclear why this happens, as the level of sea

water in the sump is not changed. One can simply turn down the skimmer settings, or elevate the skimmer drain tube to prevent the loss of sea water from the system while this is taking place.

10. Servicing the Cartridge Filters, General Instructions (Figures 2, 3G, K) :

The system has four cartridge filters (see **Figure 2**): 1) a 50µm pleated filter (**Figure 3G; Figure S3D**), 2) a biological filter (**Figure 3G**), 3) an activated carbon filter (**Figure 3G**), and 4) a small nitrate bio-pellet reactor (**Figure 3K**).

1. When servicing the cartridge filters, put the system in **standby** mode, or shut **off** the system. The main pump must be shut off. Wait for about one minute for all the sea water to drain back to the sump, once the system is placed in **standby**. Service each filter, one at a time, as needed.
2. Close valves #4, 1, 2, 6 and 5.
3. Drain sea water using valve #3 with a push-on, female quick connector and its short piece of tubing. (Use a large clean beaker to collect the sea water, which will be returned to the system.)
4. Also purge some air and additional sea water (via valve #3) from each filter by pushing the purge valves on the top back sides of the filter housings (**Figure S3E**).
5. Remove the stretchy nylon opaque sleeve, and use filter wrenches to loosen the filter bowl over a 3-5 gallon bucket to catch the sea water (**Figure S3D-F**).
6. Once loosened, use both hands to completely remove the filter bowls with their contents. Note that the filter bowls are full of sea water and media, and are very heavy. Do not let them fall and crack.
7. Be sure to reassemble filters correctly with the appropriate cartridges, gaskets, etc. and in the correct orientations. A little 95% pure (Danco #88693, Irving, TX) silicone grease may be used on the filter bowl O-rings and threads.
8. Filter bowls should only be tightened by hand. You may use the filter wrenches only sparingly. Do not overtighten the filter bowls, you can crack the plastic or seize the threads!
9. Return the valves back to their original open states.
10. Turn the system back **on**.
11. Return the collected, drained sea water back to the sump. As you will tend to lose some sea water, the sump level will be low and you will likely need to add additional sea water to the system to return the level back to normal. This can be done by simply turning on the ATO unit briefly, see **Section 33: Automatic sea water Top-Off system (ATO)**, below.

10.1. Servicing the Pleated Mechanical Filter (Figures 2, 3G):

The 50µm pleated filter (**Figure S3D**; GPE50 Keystone Giant Filter Cartridges, CECO, Telford, PA) should be changed when it becomes discolored and begins to impede sea water flow (reducing the flow rate to nearly 20 lpm, see above). These polyester filters are easy to install and have no polarity. The pleated filters typically have a life span of a few months. Note that when the pleated filter is changed, the system’s effluent pressure and flow rate should rise. The increased flow rate will tend to drop the water level in the sump, somewhat. Therefore, some additional sea water may need to be added to the system to raise the water to its nominal level. The

automatic top off unit (ATK) would also raise this level the next time (day) this unit will run. It is recommended to cover the filter bowl with dark, opaque material to discourage algal growth on this filter (**Figure 3H**).

10.2. Servicing the Biological Filter (Figures 2, 3G):

The main biological filter consists of a mix of 800 gm of Matrix (Seachem, Madison, GA) and 100 gm of de*Nitrate (Seachem, Madison, GA, see **Figure 3G)**. This highly porous media serves as a substrate for aerobic and anerobic bacteria to process ammonia, nitrite and nitrate. This quantity of media has over 1400 sq. meters of surface area to support bacterial growth. The inclusion of some de*Nitrate granules in the biofilter also helps to remove nitrates. Note that there is a small 3” diameter sponge-like filter inside the biological filter canister to help retain the pellets. The biological filter media should never need to be replaced. Typically this filter requires no servicing; however, it can be rinsed while still inside the cartridge using clean sea water and a 5 gallon bucket, if needed, to remove any debris. Never rinse this filter with fresh water as this would harm the bacteria! It is recommended to cover the filter bowl with dark, opaque material to discourage algal growth on the filter (**Figure 3H**).

10.3. Servicing the Activated Carbon Filter (Figures 2, 3G):

The activated carbon filter contains approximately 1-2 lbs of activated carbon pellets (**Figure 3G**; Kent Reef Carbon Pellets, Kent Marine, Franklin, WI). Activated carbon is a porous substance with a very high capacity for absorbing organic materials. The carbon pellets can also serve as a substrate for nitrifying bacteria. The activated carbon (Kent Reef Carbon, Kent Marine, Franklin, WI) should be replaced, as needed. This filter cartridge holds a considerable amount of activated carbon (about two pounds) and should last for a year or more. This is far more carbon than is needed and some recommend using far less or even no carbon at all. Unfortunately, there is no good way to tell when this filter is depleted. If the sea water should turn yellow, it might be time to change the activated carbon. Note that there is a small 3” diameter sponge-like filter inside the activated carbon canister to help retain the pellets, and this should be rinsed and reused if the media is changed. We recommend changing the activated carbon every one to three years. Only half of the carbon should be changed. When replacing the activated carbon, one needs to thoroughly rinse the new carbon in dH_2O to remove fine particulates. A mesh bag is provided for this purpose; however, one can also rinse the carbon directly in the cartridge using a large plastic bucket (only if all the carbon is being changed). One concern is that the activated carbon may impact the pH of the system. This issue seems to depend on the source of the carbon. One should monitor this carefully. Activated carbon may also remove certain trace elements from the sea water. Commercial preparations may be used to add back trace elements. If used, they should be added when the filters are in bypass (feeding) mode. Activated carbon may also release phosphates into the sea water, which is not desired. The activated carbon also acts as an additional biological filter, providing substrate for bacteria. One should cover the filter bowl with dark, opaque material to discourage algal growth on the filter (**Figure 3G**).

10.4. Servicing the Nitrate Bio-Reactor (Figures 2, 3K):

A small bio-reactor side loop filter (nitrate bio-reactor) has been included to encourage the growth of denitrifying bacteria (e.g., both aerobes and anaerobes, etc.) to help remove nitrogen (nitrates; see **Figure 3K**). A relatively small amount of water is slowly diverted to this side loop via the needle valve (#6; **Figures 2, 3L, Figure S2C**). Needle valve #6 should be opened enough to keep side filter loop PHA pellets moving gently. (Turning this valve clockwise will close it). Never over-tighten this valve as that may damage the needle. The PHA pellets (Vertex Aquaristik Pro-Bio Pellets, Huntington Beach, CA) must be kept moving to dislodge the accumulating bacterial byproducts in which the nitrogen is being sequestered, and to prevent the pellets from sticking together. These PHA pellets contain a source of carbon needed by the bacteria to remove the nitrates. It is best to keep the bio-reactor in a dark area away from light to prevent algal growth (we cover this canister with dark, opaque material). These nitrogen rich by-products need to be removed from the water using the protein skimmer. Ideally, the water from the side loop should be returned directly back to the sump, near to the protein skimmer's intake. To service the bio-reactor, place the system into **standby** mode or shut off the flow of water to the side loop (close valve #6).

If the flow of water is too slow or if a large amount of bacterial film and their by-products begin to accumulate, the pellets my stick together and stop moving. One can place a small 1/2" long yellow Teflon coated magnet (i.e., stir bar) inside the bio-reactor filter cartridge along with the PHA pellets. One can then use another strong rare-earth magnet, which is held externally by the hand, to move this stir bar and dislodge any stuck pellets without having to shut off water flow or open the filter. Alternatively, if the pellets refuse to move one should open the filter. To accomplish this, shut off water flow to the side reactor (Valve #6), bleed in some air by pushing the purge valve located on top of the filter holder, and then remove the filter bowl. As long as valve #6 is fully closed, it should not be necessary to shut off the main pump, or place the system into **standby** mode, to perform this service (One should verify that no water is returning back to the sump via the return hose connected to this filter, though it is perhaps safer to place the system in **standby** mode). Note that if the pump is running, some signs of cavitation may be detected. In that case, simply open up the main bypass loop just a little bit until cavitation stops (Valve #5). Note that the bio-reactor is located in a rather confined space next to the main pump, so be careful not to spill sea water when removing the cartridge bowl. Once the bowl has been unscrewed, it must be tilted to remove it; therefore, some water should first be removed using a large pipette to prevent spilling. *Be careful, the main pump motor with its electrical connection is located adjacent to the bioreactor!* Once enough water has been removed you can carefully tilt and lift out the filter bowl without spilling the remaining sea water. (By following this procedure, one should not have to remove the carbon filter to remove the bioreactor filter.) Once the bowl is removed, you will notice that the inner cartridge has a round cap and a slotted filter screen to prevent debris and pellets from leaving the filter, and these must be cleaned. Make note of their correct orientation for proper reassembly. Pull out the cartridge slowly and then slowly poor off some sea water remaining in the bowl and save this. The cartridge can then be placed back in the bowl, and vigorously moved repeatedly up and down inside the sea water remaining in the filter bowl to dislodge the bacterial film. One can also use the long plastic pipette to vigorously stir the pellets. Remove the inner cartridge from the bowl with the pellets and discard the dirty sea water left in the filter bowl. Repeat this

wash one more time with some more of the clean sea water. Only clean the little round slotted screen in the sink using distilled water and wipe clean using paper towels. Do not rinse the cartridge, bowl or pellets with anything other than sea water, so as not to destroy the bacteria! Reassemble the various part of the filter in the reverse order and screw the bowl back in place. Once the filter is replaced, restore water flow to the bio-reactor side loop (open valve #6 just enough to get the pellets moving). If they are not cleaned sufficiently, fragments of biofilm may continue to come off the pellets and be drawn into the slotted plastic filter screen, which could impede water flow, and this would need to be re-cleaned. Note, to get all the flowing sea water back into the bio-reactor's filter bowl, the purge valve needs to be pressed to remove the air (you may hear a little hiss as the air escapes). Stop pressing this button once all the air is gone and a little water starts to leak out of the purge valve. Be sure to return the main bypass valve #5 back to its previous setting when done servicing the bio-reactor. Some additional sea water will need to be added back to the system to replace that discarded during cleaning. It is a good practice to keep an eye on the bio-reactor and to periodically dislodge or clean these pellets.

The consumable PHA media in the small side loop filter should be replenished, as needed, when it is consumed (this typically has a very long lifespan equal to several months). Only about ¾ inch depth of fresh static media should be placed in the small side loop filter bowl (**Figure 3K**). The volume of the fresh, slowly tumbling consumable media will appear to be about 1 inch deep when the sea water is flowing. One must to be careful to keep these pellets moving, and not to add too great or too small a quantity of these pellets. It is far worse to add too much than too little. Too much PHA media, too little water flow (impeded movement of the pellets) or insufficient levels of oxygenation can create toxic levels of hydrogen sulfide. We have found that these pellets are reduced to approximately half their diameter after about 3-4 months of use. At that point some additional fresh pellets need to be added. When adding new pellets, do not discard the old pellets, which have the necessary bacteria growing on their surfaces.

<u>11. Monitoring Water Quality:</u>

Users are required to check the health of the biofilters by measuring the levels of ammonia, nitrites and nitrates on a regular (weekly) basis. We also measure levels of phosphate, general water hardness (KH), calcium, and pH regularly. Kits are also available to measure various specific water quality parameters. To monitor these levels we use test kits produced by API Marine (Mars Fishcare North America, Chalfont, PA), following the manufacturer's instructions. Kits and instruments are available from many other manufacturers, but we find these kits to be good compromise between cost, ease of use, and accuracy.

For animals that secrete calcareous elements (e.g., gastropods, urchins, corals), calcium levels should be maintained at high levels (~400ppt). In our previous system (Henry et al. 2017), a large bed of crushed coral provided useable calcium (measured at 380-420ppm), in addition to buffering capacity to maintain pH levels. In the recirculating system, the combination of reduced volume of crushed coral (**Figure 4M**) and a rapidly growing snail population reduced calcium levels, in turn increasing water hardness (KH) and pH. To address immediate concerns (after 8 months in service), we performed a series of partial water changes (maximum 20% of water volume at one time). Over the longer term, we have initiated the use of an ionic calcium

supplement, to increase calcium levels without affecting pH or carbonate levels. We use Reef Advantage Calcium (Seachem Laboratories, Madison, GA) to maintain calcium levels at 380-420 ppm and balance water hardness and pH, following the manufacturer's recommendations. Calcium chloride should be added slowly to the system, per the manufacturer's instructions, as excess doses can affect carbonate alkalinity and pH. Once calcium levels are stable, the user can determine how much supplemental calcium should be regularly added to the system, which will vary depending on population size and growth rates.

Many aquarium users add kalkwasser as a source of calcium, however, this can impact alkalinity (KH) and pH, and can be more challenging to dose. Water quality parameters should be checked regularly (weekly), and additional calcium added as required. For urgent situations, water changes (no more than 20% of the total volume at a time) can also be implemented. A calcium reactor may also be added to the system to automatically regulate calcium, alkalinity, and pH in the system.

12. Servicing the UV Lamp and Quartz Sleeve (input from R2, Figures 2, 3I):

(Currently not in use)

The UV lamps consists of a 57 watt Aqua Ultraviolet lamp (**Figure 3I**; part number A20057, Aqua Ultraviolet, Temecula, CA) contained inside a 19" long Classic quartz sleeve (part number A10057, Aqua Ultraviolet, Temecula, CA), which is located inside the plastic housing (**Figure 3I**). Power to the UV lamp is controlled by relay (R2).

*Turn **Off** the system to avoid electrical shock!*

The UV light helps sanitize the water by killing circulating microorganisms. The sanitizing efficiency of UV light is related to UV intensity and water flow rate (e.g., UV exposure amount/time). UV output diminishes with use, and therefore, the UV bulb has a limited lifespan and should be replaced once each year. In addition, the quartz sleeve that protects the UV bulb must also be inspected and cleaned if deposits have formed, whenever the bulb is changed. UV operating status is indicated by Alarm light A2. UV bulb illumination can also be verified by the presence of a pink/blue light seen through the transparent blue plastic bell located at the top of the bulb housing (**Figure 3I**). The UV bulb is 57 watts and is designed to have a 95% efficiency at the end of its 1 year rated lifespan (9000hrs; equal to 110μW/cm_2). At that time the quartz sleeve should also be inspected and cleaned, as needed. These parts are to be removed separately, starting with the UV bulb. First the system should be turned **off** and unplugged. The water should be allowed to drain back to the sump and main outlet valve #1 and sensor loop valve #2 should be closed (**Figure 2; Figure S2B**). The UV bulb may be removed alone without opening up the quartz sleeve sea water compartment by loosening the black nut located just above transparent blue plastic bell (**Figure 3I**). Only handle the UV lamp and quartz sleeve with clean gloves. Once loose, the bulb will slide straight up out of the holder along with its electrical connections. After the UV bulb is removed one can also remove the quartz sleeve by loosening the transparent plastic blue bell (**Figure 3I**). Once loose, the sleeve, which looks like a giant test tube, will slide straight up out of the holder. Note that unlike the UV bulb, the outer surface of the quartz sleeve is exposed directly to the sea water. The quartz sleeve may be dried and wiped clean using cotton wipes or cheese cloth. If scaled, some mild vinegar may be used to

dissolve the calcium deposits located on the outside of the sleeve only! Rinse the outside with distilled water when done. Do not let any liquid enter the quartz sleeve or contact the UV bulb or its electrical connections! They must remain completely dry. The bulb and sleeve are very fragile. Great care must be taken so that they are not scratched or broken. The bottom, closed end of the quartz sleeve is particularly fragile. Reassemble these parts in the reverse order, making sure that the fittings have been tightened to prevent leaks.

The UV bulb must be cooled by running sea water, otherwise the schedule 40 PVC black plastic housing will melt. Do not operate the UV lamp, if there is no running sea water! The UV ballast, which is contained in its own separate plastic housing, should last for several years (**Figure 3J**). When it fails it typically shorts closed and the circuit's fuse will blow. Note that the same fuse also supplies power to the main pump motor, so the water pump will also stop, triggering a no/low flow condition that will shut down the system.

** The UV lamp provides for a very aseptic supply of water to the tanks. This may limit the number of beneficial bacteria in the system but it should help limit the spread harmful microbes or other planktonic organisms. In the past, we have never needed to worry about problems with such contamination when using simple 30 gallon aquaria. Hence, it may prove that we do not require the UV lamp. Use of natural sea water, operation of the system near natural marine sources of contamination, or culture of more sensitive organisms may necessitate use of the UV lamp. So far, we have never turned on the UV lamp. Any effect of the UV irradiation on the phytoplankton food added to the system, is undetermined.

13. Aquarium System Temperature Regulation (heater regulated by S14, input from R6, Figures 2, 3E):

A titanium heater (Finnex TH500-X, 500 watts, Chicago, IL) is located on the floor of the left sump compartment, just below the protein skimmer (**Figure 3E**). Heater status is indicated by Alarm light A6 (active when illuminated). As supplied, our aquarium system has no active cooling, and the only controlled input is from this heater, which can only be used to raise the water temperature. (However, see additional external chiller, below). The activity of the heater (R6) is regulated by readings on the temperature probe located within the pH probe (S14). (Alternatively, it can be set to be regulated by the temperature probe located within the conductivity sensor (S13)). In addition, the main pump and the UV lamp provide a constant source of heat. To some extent, aquarium system temperature is related to room temperature. As room temperature rises, so can the water temperature. Room temperatures must, therefore, be kept low for maintaining tropical animals. Ideally, the room temperature should be 18-22°C. While the inclusion of covers on the tanks and the sump will limit evaporation, they will also trap heat and limit the rate of cooling. Therefore, without a chiller, or a cold room temperature, it is not possible to completely seal the aquarium system from evaporation, as it will begin to overheat. Ideally, aquarium water temperature should be maintained at 27°C for *Crepidula atrasolea* snails. Note that the readings on the main system controller may not accurately reflect those inside the individual tanks. Temperatures should be verified using a reliable thermometer. Snails can tolerate a temperature range of 23-29°C, but the extreme

temperatures put a great deal of stress on the animals and will kill them. Alternatively, one can use a small aquarium chiller to lower temperatures, as needed (see below).

If the room temperature is going to get too hot (e.g., hot summer months, failure of building air conditioners, etc.), it will be necessary to install a water chiller. The best configuration is to have the chiller installed during production, with the chiller loop located directly inside the sump and regulated by a common temperature control circuit used for the heater. Alternatively, one can tap off of the system to divert some pressurized water to a small chiller, as described below (**Figures 2, 4C, K-L; Figure S2B**). Please note that chillers will also generate their own heat, which can elevate room temperature even further. The water chiller does not need to be used at all times (see below), but is a permanent installation to the system. Chillers require a specified flow of sea water typically of at least 3 gallons/min. The chiller must also be rated to handle at least 25 PSI of inlet water pressure, which is the maximum rated output pressure of the water pump, though in practice water pressure to the chiller will be much lower. One may may need to use a pressure regulator to lower the input pressure to the chiller, and reduce the flow rate to one specified for the chiller. As we supply water to an external chiller via the sensor side loop, there is little concern, as pressures there are much lower. Input water to the chiller can also come from the Acc. Tap filler hose (**Figure 2; Figure S2E**), which can deliver greater pressures and flow rates (controlled by valve 7). The chilled water needs to be returned directly back to the sump (**Figures 2, 4N**), and those lines should be enclosed with foam insulated tubing secured with zip ties (**Figure 4K-L**).

<u>14. External Chiller System (Figures 2, 4K):</u>
An external 1/10 hp titanium chiller (JBJ Arctica DBA 075, JBJ Aquarium, Inglewood, CA; **Figures 2, 4K**) has been installed to lower sea water temperatures, as needed. The chiller is a simple flow-thru system and does not have an internal pump. Therefore, one must feed the chiller with pressured water from the rack system. To accomplish this, the chiller receives the effluent sea water from the sensor side loop (**Figures 2, 4C, K-L;** see **Additional File 3E**), which ranges between 10-14 psi or lower as the pleated filters become clogged. Note that this particular chiller does not require a pressure regulator, as the pressures are below the maximum rated safe pressures. The chilled water is returned directly back to the sump using the side loop's final effluent tubing connection located directly in the sump cover (**Figures 2, 4N**). The chiller will maintain cold water temperatures to within 1°F of its set point. Note that unlike the rack system, the chiller readings/settings are in °F. It is important to set the chiller to a temperature above that normally desired for the system (i.e., the heater temperature setting), so that the chiller and heater do not end up competing against one another. For example, if the desired system (heater) temperature is set to 27°C (80°F), then the chiller should be set to 28-29°C (82-83°F). This ensures that it only turns on when the sea water temperature well exceeds that of the desired heater temperature, and does not chill the water below that of the normally desired heater water temperature (which would in turn re-activate the heater, ad infinitum). The actual setting of the chiller must be determined empirically. We have the chiller set to 82°F which will be activated at that temperature and will turn off once the water reaches 81°F. For accuracy, the chiller's displayed temperature reading must initially be calibrated, as described by the manufacturer (**Figure 4K**). The chiller is essentially a permanent installation and can be

left on at all times. It will only be activated when the water temperature rises above the chiller's set point. Alternatively, the chiller power can be turned off (un-plugged), if warm room/water temperatures are not expected. Sea water will simply flow unimpeded through the chiller's plumbing system. The chiller water flow and hose connections should be inspected periodically and back-flushed, as recommended by the manufacturer.

Note, that the addition of the chiller reduces the flow of water through the sensor side loop. Currently, with the chiller installed, far less than ten gallons per minute flow through the chiller and side loop when in feeding mode (and even less when in filtering mode). The chiller actually specifies a 960 liters per hour, maximum flow rate (16 liters per min). While system flow rates range from 20-33 liters per minute (depending on the mode and condition of the filters), the flow through the sensor side loop is much less and will not exceed the chiller's specifications. If the flow rate is too slow, however, it could cause the sea water to freeze, blocking flow to the chiller and sensor side loop (sensed by the float valve; **Figures 2, 4C**), which would activate a system alarm. The chiller has built in protection to sense when the coils become too cold, which will shut down the chiller (but not the aquarium system). As a safety precaution, the sensor side loop float switch could be wired to control power to the chiller, such that if water flow is interrupted, the chiller could be powered down using an accessory circuit. There is an unused relay within the Walchem 900 controller that can be used for this purpose (R8). Note that if water flow is interrupted to the sensor side loop, those sensor readings will begin to change dramatically. This will have an impact on the regulation of temperature, conductivity and pH, which could harm the animals. Alternatively, one could add the appropriate fittings to supply water to the chiller by using the accessory filler tap (valve # 7; **Figure 2; Figure S2E**); however, this would rob the tanks of some water flow. In this case one would also need to install a new drain port in the top of the sump cover to return the chilled water back to the sump.

Never tilt or turn the chiller (compressor) onto its side. If the chiller is moved or tipped over, it should be left standing upright for at least 4 hours until the power is restored, to allow the oil to return back to the compressor.

<u>15. Servicing the Sensors (recalibration, Figure 4C; Figure S4):</u>

When servicing the sensors (e.g., for cleaning or recalibration) in the side loop, one can simply shut off the sensor side loop ball valve (#2) by turning it clockwise 90 degrees, see **Figure S4A**. (Be sure to Disconnect power to the chiller first, as one should never let the chiller run without flowing sea water). Shutting off the side loop water flow alone will not place the system in a **standby** mode, as the main pump will continue to run, but the flow switch (D3) will immediately register a "No flow" alarm. Pressure on the effluent water gauge will rise 2-3 psi. Alternatively, one can put the system in **standby** mode, which will shut down all water flow, and both the flow sensor (D3) and the paddle wheel flow sensor will immediately register "No Flow" alarms. Note that during re-calibration, various alarms may be thrown, as the sensors in the side loop will respond to changes in water conditions. This is due to the fact that when water flow is shut off to the sensor loop, the readings for temperature will begin to fluctuate and those for conductivity and pH will tend to drop over time. In addition, when the system is placed in **standby** mode the water level in the sump will rise by a few cm, as water drains back

from the tanks, which may trigger a high water level alarm. However, while performing the actual sensor calibrations when inside the Walchem 900 controller's menu driven system, alarms and any automatic adjustments of water conditions will be temporarily suspended. Afterwards, be sure to restore flow to the side loop gradually by slowly turning back on valve (#2). If the flow switch does not rise back upwards to signify flow, the no flow alarm will remain active. In that case, one can simply turn of the valve (#2) and turn it back on more quickly. As long as the flow switch is lifted, the sensor loop is getting enough water flow. (In addition, be sure to Restore power to the chiller).

As noted above, sensor readings can change with changes in flow rate and will drift in static water when water flow is interrupted to the sensor side loop or the system is placed in **standby** mode. It may take some time for these readings to stabilize and return to normal after water flow is restored (one to two days), so one should monitor the system very closely until they are stable. It may be necessary to temporarily place the RO and pH pumps in HOA **OFF** mode, until the readings have stabilized so that they do not change the desired parameters (see below).

15.1. Calibration of the pH probe (output to S13, Walchem 191653-20, Figure 4C; Figure S4B-D):

The system is equipped with a Walchem pH sensor (**Figures 2, 3C; Figure S4B**; model number WEL series 191653-20, Iwaki Aquatics, Holliston, MA). The pH probe must be calibrated periodically, as needed, and whenever it is replaced (once each year). This is accomplished by entering the calibration menu on the Walchem 900 main system controller. Follow the instructions. The probe must be removed for this process. Calibration is exactly like that of a standard lab pH meter. The sensor is removed by loosening the large grey plastic union using ones hands or a filter wrench (**Figure 4C; Figures S1, S4B-C**). A two-point calibration is performed using both pH 7.0 and 10.0 calibration solutions (Walchem part number 51370, pH 7.00 and Walchem part number 51371, pH 10.00, Iwaki Aquatics, Holliston, MA; **Figure S4D**). These calibration solutions have a limited shelf life. Be sure to rinse and dry the probe thoroughly between solutions and before reinserting it back into the sensor loop. Follow the Walchem 900 on-screen instructions. The pH probe plugs into the pH probe preamp. While the probe itself is exposed to sea water, under no circumstances can any water contact the internal preamp connections! The probe simply plugs into the upper preamp. Therefore, the pH probe must be thoroughly dried if one is replacing the pH probe element. When reinstalling the probe, turn it so that both the thin metal temperature sensor and the larger pH probe are both exposed to the cross direction of flowing water (see **Figure S4C**). Retighten the PVC union to prevent leaks! Do not over-tighten this fitting. Note that unlike the conductivity sensor, the pH probe should be replaced every year. Actual system pH should be 8.3, but one may find the system readings to be off somewhat. Actual pH should be verified independently using a second device. The output pH pump set point for regulating pH level (if activated) should be adjusted accordingly to achieve the actual desired pH. Note, however, that we have not been using the pH dosing pump to regulate pH, as this was found to be unnecessary.

15.2. Calibration of the Conductivity Sensor (output to S11, Walchem #103906-10, Figure 4C; Figure S4B, E):

The system is equipped with a Walchem contacting conductivity sensor (**Figures 2, 4C; Figure S4B, E**; model number 103906-10). This sensor has a range of 0-300,000 µS, with a resolution of 10µS, but is designed specifically to monitor higher salinities (Iwaki Aquatics, Holliston, MA). The conductivity sensor must be calibrated periodically and when it is replaced. The probe must be removed for this process. Calibration is accomplished by entering the calibration menu on the main system controller. The long, thin stainless steel sensor is removed by loosening the upper-most black plastic compression nut (**Figure 4C, Figure S4E**). Two adjustable wrenches are needed to ensure that the lower black plastic fitting does not come loose (**Figure S4E**). To calibrate, enter the calibration menu on the main system controller. Follow the Walchem 900 on-screen instructions for calibration. A single calibration solution (Part number YSI 3167, 060907, YSI, Inc. Yellow Springs, OH) is used with a conductivity of 1000µS/cm at 25°C. These calibration solutions also have a limited shelf life. We use a shortened graduated cylinder (plastic 100ml cylinder with the top half removed) to calibrate this long sensor (**Figure S1**). Be sure to rinse and dry the probe thoroughly with dH_2O before and after calibration and before reinserting it back into the sensor loop. When returned back to the system, the probe is inserted all the way down into the opening. Retighten the PVC compression nut to prevent leaks! Do not over-tighten this fitting! Actual system conductivity should be around 50.6msem/cm2. tThis reading depends on the temperature (Specific gravity is equal to 1.024). The conductivity sensor should have a long lifespan of several years. Given the single point calibration, one may find the readings to be off somewhat. Actual conductivity should be verified independently using a second device. The RO pump output set point for regulating conductivity should be adjusted accordingly to achieve the actual desired level.

15.3. Potential Problems after Calibration!

We have found that the readings can change dramatically immediately after recalibration, even when the actual sea water parameters have not changed at all. These issues occurs for some few hours or even up to two days after calibration. Readings should eventually stabilize. However, one may need to readjust the alarm set points, as well as the set points of the appropriate control relays and monitor these over several hours or even days, using other meters to validate these readings and ensure the adjustments are correct.

***Readings made by the pH sensor can slowly drift over time, and those readings need to be carefully monitored! We have generally found these readings to get higher over time. Any adjustments need to be made accordingly! We find it best to recalibrate these probes when the readings are found to have drifted significantly. Currently, we do not rely on this sensor to make any automated system adjustments.*

***The Conductivity sensor can also deliver erratic readings, and those readings need to be carefully monitored! Any adjustments need to be made accordingly! In our experience, we have found these readings to eventually stabilize after recalibration and can stay fairly constant for long periods of time. We find it best to recalibrate this probes only when the readings are found to have drifted significantly. Double check all readings using separate equipment, such as that shown in **Figure S1**!*

16. Double Checking the Sensors:

We periodically verify sensor readings using a glass thermometer, as well as handheld digital pH and conductivity meters (see List of Parts and Suppliers, as well as the tools shown in **Figure S1**).

17. Sea Water:

The system uses Instant Ocean Reef Crystals artificial sea salt (Spectrum Brands, Inc. Blacksburg, VA, UPC code 51378 01800). This sea water is mixed using RODI to a specific gravity of 1.024 and a pH of 8.2 to 8.3 at 27°C, with the addition of Replenish (Seachem, Madison, GA), following the manufacturer's instructions to maintain general water hardness. Note that we have found that *Crepidula* snails raised using the standard formulation of Instant Ocean sea salt developed shell defects (Henry et al., 2017). Instant Ocean Reef Crystals sea salt is formulated with higher concentrations of calcium and trace elements.

18. Bags of Crushed Coral/Shell (Figures 2, 4M):

Two nylon mesh bags (Acurel 8" x 13" Filter Lifeguard Media Bags, Acurel, Cranberry, NJ) containing a total of 15lbs of crushed coral/shell are placed to the far right side in the system sump to serve as refugia for bacteria, and as a source of calcium for added buffering and to support shell growth. There should be no need to service the crushed shell/coral, though one might inspect the mesh bags to be sure they have not split open and spilled any shell/coral debris. If any debris were to be sucked into the main pump, it would destroy the impeller. These bags can be viewed through the sump window, with the help of a flashlight (**Figure 4M**).

19. Accessing the Right Sump Compartment (Figures 2, 3A-B; Figure S3A-C):

If it is necessary to gain access to the right sump compartment, one must remove the slotted PVC drain diverter (**Figure 3A**). Place the system in **standby** mode and let the system sit for 10 mins to allow all the water to empty back into the sump (otherwise water will drip down the back, outside of the sump once the slotted drain divertor is removed (see below). Remove the sump Origami cover (**Figure 3A-B**), and loosen the union joint that secures the diverter completely to remove the diverter. Remove the filter pad (It holds a lot of water, so let the pad drain on edge to allow the water to flow back into the sump, do not squeeze it dry, as you will release any trapped debris). Tilt up the entire top sump cover frame about 1-2" inches in the front, only enough to slide out the plastic, perforated filter pad plate (**Figure S3C**), We have added a small plastic zip tie handle to facilitate its removal. Remember that there are several other connections to the top sump cover and one must be careful not to stress these. Reassemble all the components in the reverse order, and be careful to retighten the slotted drain diverter union to prevent any leaks.

20. Main Pump Inlet, (Figures 1L, 2; Figure S2A):

Water circulation is provided by an Iwaki seal-less magnetic drive pump (MD-70RLZT) with a polypropylene centrifugal impeller (**Figure 1L**). Water to the pump flows through ball valve # 4 (**Figure 2; Figure S2A**). This 2/7 hp pump has a rather steep pump curve with a maximum capacity of 11.4 gallons per min and a maximum head pressure of 66.6 feet. This pump can deliver a maximum system pressure of 42.7psi. The main pump inlet located within the sump

should have a plastic, slotted guard to prevent large debris from blocking the opening and being sucked into the pump. This inlet guard should be swiveled to point downwards inside the sump. Inspect periodically to make sure the guard is still in place and not clogged. Note that outlet valve #4 delivers water pulled in from this opening to the main pump and should be completely open during **normal** operation.

The pump should never be allowed to run dry. Several safety features are built into the system to prevent this from occurring. A simple electromagnetic float valve monitors flow through a sensor loop (**Figures 2, 4C**). A separate flow meter also monitors flow rate through the entire system (**Figures 2, 4N; Figure S2D**). If the latter sensor detects reduced flow, the pump will be turned off. Likewise, the sump has an ultrasonic water level sensor (Flowline DL10-01 Ecopod, Los Alamitos, CA; **Figures 2, 4O**). If the sump water level falls below a certain level, the pump will be shut off.

<u>21. Pump Impeller Cavitation:</u>
The main pump (**Figures 1L, 2**) is controlled by relay R1. Cavitation occurs when the main pump inlet pressure drops, and the outlet pressure rises. Under the lower inlet pressures, the inlet water can flash into steam and many bubbles will form, which then collapse violently causing shock waves that pit and erode the pump housing and impeller. This condition is very destructive. Cavitation is noted by a loud grounding noise. The sound is loud and very distinct: sounding like gravel or sand is moving through the pump. Cavitation can occur when the pump is starved of inlet water (e.g., pump inlet valve #4 is not completely open), the sump inlet cage is clogged, sump water level is too low (below the pump's cut water level), or air has entered the inlet side from leaks in the PVC unions. Higher differential pressure on the outlet side may also cause cavitation, such as when the filters are clogged or outlet valve #1 is not completely open or there is not enough bypass water returning back to the sump via the side loop, sensor loop or main bypass loop to help reduce outlet pressure (**Figure 2**, see **Section 30: The Main Bypass Valve**, below).

<u>22. Drain Valve (#3) and Accessory Water Filler Tap (Valve #7) (Figures 2, 3L; Figure S2C, E):</u>
The rack system includes a main drain port (Valve #3), as well as a filler port (Valve #7) that can be used to remove sea water from the system, should water changes be necessary (**Figures 2, 3L; Figure S2C, E**). Valve #7 and its attached blue hose (**Figure 2; Figure S2E**) can be used to withdraw small or large amounts of sea water, if needed, when the system is running. Note that the water will exit with considerable pressure. This valve can also be used for partial water changes to remove water from the system (see **Section 23: Automatic Mechanical Float Filler Valve**). Valve #7 can also be used to drain the system to the cut level of the main pump. Likewise, drain valve #3 (**Figure 3L; Figure S2C**) can be used to tap water or to empty the system, but be aware that the water will also exist with considerable pressure while the main pump is running (Not recommended!). To open this valve, one must insert the quick connector with an attached hose, and be certain to secure this hose so that it does not get loose (**Figure 3L; Figure S2J**; all hoses should be secured with hose clamps!). **Warning**! If the main pump is running, water will leave this valve with considerable pressure. It is not recommended that valve #3 be used to drain the system while the water pump is on, as that water is under very

high pressure. It is best to use the accessory filler valve (#7) for this purpose. Water can also be drained from valve #3 by gravity alone, when the pump is not running, and the system is shut **off!** Note that if the system is being emptied completely, any remaining water in the sump located below the opening of the main pump inlet would have to be removed from the sump by siphon or scooped/soaked up after the main pump is shut off. Do not run the main pump dry, or let the main pump suck air at any time!

<u>23. Automatic Mechanical Float Filler Valve "FV" (for addition of sea water if the level should drop, Figure 3D):</u>

(Currently not in use)

The sump is equipped with a simple, mechanical float valve for topping off the system, should the water level drop below a certain level (**Figure 3C-D**). Make sure the end of the short white tubing fitted to this valve stays elevated well above sump water level when not in use to prevent any possible leaks, as the valve opening is located at or just below the normal sump water level! Note that this valve could be used for automated or semi-manual, weekly water changes (approx. 10% per week) to reduce the levels of nitrates (see notes related to **Accessory Water Filler Tap (Valve #7, Figure 2; Figure S2E)**. These types of mechanical valves are somewhat unreliable, as they may stick open or closed. In addition, these float valves do not have very tight dead-bands for precise control of the water level. Therefore, we do not use this feature. Instead, we have installed an automatic top off unit that uses redundant optical sensors to detect changes in the water level and a small electromechanical pump (see below).

<u>24. Float Switch (output to D3, Figures 2, 4C; Figure S4B):</u>

An electromechanical float switch ("ft") senses if there is some flow in the system (**Figures 2, 4C; Figure S4B**). The switch incorporates a magnetic Hall effect sensor. This component has one moving part and generally does not need any service unless it should get stuck in either the open or closed position.

<u>25. Servicing the Paddle Wheel Flow Sensor (output to D2, Figures 2, 4N; Figure S2D):</u>

The system is equipped with a paddle wheel flow sensor to monitor the sea water flow rate (**Figures 2, 4N**). If it is necessary to remove the paddle wheel flow sensor "fm", be sure to shut **Off** the system! Otherwise, this sensor will be ejected with great force! Next, drain system water as described under **Section 10: Servicing the Cartridge Filters**. Remove the left side grey PVC plastic sump cover (**Figure 4N**), angling so that any residual water will drip into the sump. Remove the stainless steel U-shaped retaining wire by pulling it to the right. This part is marked with a yellow warning tag (**Figure 4N; Figure S2D**). Then pull the cylindrical sensor straight out while bracing the sensor housing with your other hand. Be careful not to jostle the PVC pipes and fittings, which are poorly supported in this area -- otherwise a leak may develop. One can rotate the sensor slightly to help ease it out. If needed, one can use a small flat blade screw driver to help pry out the sensor to get it started, but be careful not to chip/scratch the plastic. When done, reinsert the sensor and the retaining wire. Some silicone grease may be used on the sensor O-ring, if needed.

26. Calibration of the Echopod: Sump Water Level Sensor (output to S21, Figures 2, 4O):
An ultrasonic sensor is located in the lid of the sump to detect the sump water level (**Figures 2, 4O**). A long PVC tube, or "stilling well," is attached to this sensor to keep the water level steady for more accurate readings. The water level readings can be calibrated using the main system control box, as needed, but generally no adjustments should be required once this is set up. The small exposed vent hole for the Echopod's stilling well should be kept open. Remove any sea salts that may have dried inside this opening. The water level can also be visualized directly through the window located within the front panel of the sump (**Figure 4M**). Once everything has been set up, a normal operating water level line (for filtering mode) should been printed on a sticker affixed along the side of this window for quick reference. When the system's main pump is not running, however, the sump water level may not be visible as it will rise above the top edge of this window.

27. Preventing Evaporation:
Seams located around the lids to the tanks and the sump, and large openings over the drainage troughs and sump, permit evaporation and salt accumulation. One should make sure that all openings are covered (**Figures 1D, G-I, K; 3B; see Additional File 3B-D**). This includes plugging the small unused holes located in the tank lids. Covers are also located on the trough drains, down spouts, the sump filter pad area (i.e., the clear plastic "Origami" cover) and above the sump heater and protein skimmer compartment. To limit evaporation from the drain troughs, we machined a set of PVC covers that contain a series of smaller holes to accept the tank drain pipes (**Figure 1H-I**). However, sealing the system increases system temperature, as described in **Section 13: Aquarium System Temperature Regulation**, above.

28. Sump Covers (Figures 1K, 4N):
There are two covers for openings in the top of the sump. One smaller, grey PVC plastic cover is located over the left sump compartment that houses the heater and skimmer, and was provided by Iwaki Aquatics, Inc. (**Figures 1K, 4N**). This cover should always remain in place and has a small slot added along the back side for the skimmer power cord. Another small hole accepts the Teflon feeding line. The right compartment that carries the coarse filter and perforated filter plate and receives the return drain water has a 1/8" thick clear polycarbonate "Origami" style cover to prevent splashing and some evaporation (**Additional File 3D,** see warnings above, regarding increasing water temperatures described in **Section 13: Aquarium System Temperature Regulation**). There is not a lot of room above the sump in this area and this special design was needed to fit over this opening. The Origami cover is fashioned from a single piece of 1/8 inch think polycarbonate folded on a sheet metal break (see **Additional File 3D** for diagram). In addition, a small piece of Saran wrap is first draped over the slotted PVC water diverter to cut down splashing (**Figure S3A**).

29. Salt Creep (Figure 4G, J):
Dried sea salts will collect wherever sea water is able to escape the system (e.g., **Figure 4G, J**; around drains, covers, vent holes, etc.). These should periodically be removed by scraping and using a damp cloth. Significant measures have been taken here to limit the effects of water loss

and salt creep (see **Sections 5: Servicing Individual Tanks, 27: Preventing Evaporation, 28: Sump Covers**, and **33: Automatic sea water Top-Off system).**

Note also that one can lengthen the plastic tubing that supplies sea water to each tank. If the ends are located below the water surface, there will be less splashing and less salt creep will form around the lids and plugs. Keeping the tubes shorter helps with oxygenation and allows the user to more easily verify that there is water flow. The drawback to this approach is that one will not be able to easily judge how much water is flowing to the tanks, and there may be somewhat less aeration of the water.

30. Main Bypass Valve (V5, Figures 2, 4N-O; Figure S2D):

The system includes a main bypass loop controlled by valve #5 (**Figures 2, 4N-O; Figure S2D**). This loop/valve allows some sea water to return directly to the sump, and needs to be adjusted to prevent excessive outlet pressures and pump impeller cavitation. Outlet pressure can be monitored using the round effluent pressure gauge. Presently, during Filtering mode this pressure runs at about 10-11 PSI. During Feeding mode, it climbs to approximately 13-14 PSI. So far, we have not needed to use the bypass loop and have kept this valve #5 closed, since enough water usually returns to the sump via the tanks, side filter loop and the sensor loop. As more tanks are brought on line, there is less need to use the main bypass loop, as even greater water flow returns via the tanks, side filter loop and sensor loop. In this case, the valve can be left closed. However, if one reduces the flow to the tanks or uses a smaller number of tanks this valve would need to be opened, just enough to drop outlet pressures and to stop cavitation from occurring.

31. Metal Components Exposed to Sea Water (Risks of Corrosion):

Certain metal components are continuously exposed to sea water that need periodic inspection for corrosion. These include the stainless steel bolt securing the handle of the sump lid, the stainless steel outlet pressure gauge, the ferrite rotor for the automatic top off ATK pump, the stainless steel conductivity sensor, a stainless steel hose clamp securing the hose fitted to valve #7, and the stainless steel temperature probe included with the pH probe. Other parts may possibly be exposed to splashing or dripping sea water, including the stainless steel leveling feet and aluminum foot pads, the stainless steel reinforcing bands located around each fitting for the small ball valves, and the aluminum rack. Note that the titanium heater is relatively impervious to the effects of sea water.

32. Reverse Flow Check Valve (Figures 2, 3L; Figure S2I):

The reverse flow check valve (“CV”) is a gravity and flow/pressure operated mechanical valve that prevents water from entering the back side of the main filter loop during the feeding mode (**Figures 2, 3L; Figure S2I**). It generally should not require any service. The housing is clear plastic and one can visually inspect the valve flap to see that it opens (in filtering mode) and closes (in feeding mode) appropriately.

33. Automatic Sea Water Top-Off System (ATO, Figures 2, 4H-J):

This system consists of a Neptune Systems ("ATK") Automatic Top-off Kit (ver. 2, Morgan Hill, CA) automatic top-off kit, which automatically adds artificial sea water to the sump to compensate for any drop in sea water level due to salt loss (**Figures 2, 4H-J**). Water spray, splashing and salt creep eventually reduce the overall volume and additional sea water must be added to raise the sump level to allow for proper operation of the protein skimmer, etc. The ATO unit consists of a microcontroller, two optical water level sensors mounted in a magnetic bracket, a small self-priming water pump ("PMUP"), plastic tubing and an anti-siphon break. The reserve sea water is stored in a 5 gallon plastic paint bucket (**Figures 2, 4H-I**). Two small holes are drilled into the lid to allow for passage of the two sensor wires, motor power cord and plastic water line. Freshly prepared artificial sea water is added to the bucket, but never all the way to the top (see below). Depending on the relative heights of the water in the sump vs. the external reservoir, an anti-siphon break must be installed in the tubing. The anti-siphon break must be located inside the reservoir bucket above the level of the reserve sea water! It has a small hole for the side vent that must angle downward so that ejected sea water will be directed back into the reservoir. It is normal for some sea water to be expelled from this hole during pumping. The small PMUP water pump is placed upright in the bottom of the bucket (**Figure 4I**). As precipitate can form in artificial sea water, a small sieve is used to protect the pump. For this purpose, we place the pump intake inside of The Republic of Tea's "The People's Brew Basket" (Novato, CA) nylon mesh tea strainer, which is 3" in diameter and 2-1/2" tall (**Figure 4I**). The 24 VDC pump has a magnetic drive impeller and was originally designed to pump RODI water. The impeller shaft has a magnetic drive impeller with an exposed ferrite rotor. These ferrite ceramic magnets have excellent corrosion resistance. We have not experienced any problems with corrosion, but it is best to periodically monitored this on a regular basis to make sure the rotor spins freely. To prevent additional pumping from occurring during a feeding cycle, which temporarily lowers the sump's water level, the microcontroller is put on a 24 hour timer (**Figure 4H**), so that it only runs once each day for 30 mins (during a non-feeding, filtering cycle). For extra security, this time window is set to operate during normal working hours so that operators are more likely to be present to observe any problems that may occur with water delivery. When the unit is on a blue light will be illuminated on the microcontroller. During pumping a green light will be seen. Any problems will trigger and audible alarm and a red light will be illuminated. Whenever an alarm is sounded, the unit goes into arrest and the power must be cycled to reset the ATO.

Two optical sensors monitor water level in the sump. These are mounted in a magnetic bracket located inside the front right corner of the sump (**Figures 1K, 2, 4M**). The lower sensor is the main sensor and the upper sensor serves as a redundant backup, should the main sensor fail. (Due to space constraints, an additional backup in the form of a small mechanical float valve, has been removed from the bracket.) Due to the presence of the sump filter pad and sieve plate, the bracket had to be rotated 180 degrees for better clearance. This simply reverses the two optical sensor inputs that need to be plugged into the microcontroller (# 1 and #2). These two electrical plugs have been re-labeled so that they now correspond to the proper numbers on the microcontroller. The bracket for the optical sensors must be positioned at the correct height for the desired sump water level to be achieved. A strong magnet and sticky rubber pads hold the bracket in place (**Figures 2, 4M**). The correct water level should be located at the

center of the pyramidal lens covering the main (lower-most) sensor. To get the proper adjustment, this has to be done while the system is running and during a non-feeding cycle. The course filter pad and sieve plate will need to be removed (see **Section 19: Accessing the Right Sump Compartment**). Once correctly positioned, the external orange magnet must not be moved. From time to time, pre-pared artificial sea water will need to be added to the 5 gallon plastic reservoir bucket. Sea water added to the system should be prepared at least a day in advance to allow the salts to dissolve and be able to adjust and stabilize the salinity. Do not mix artificial sea salts directly in the reservoir. A third optical sensor is installed in this reservoir to monitor its sea water level, and to ensure that the pump will not run dry. When low, the alarm will sound, alerting users to add more sea water. Again, if there is an alarm, the power must be interrupted to reset the system (which is normally accomplished each day by the 24 hour wall timer). This third optical sensor is held in place by a small magnet, so care is needed to make sure the position is not changed (**Figure 4I**). The sensor should be set approximately two inches from the bottom of the reservoir: the cut level of the small pump. There are no settings that can be adjusted on the stock ATK microcontroller; however, more complex control can be accomplished, if this unit is attached to a Neptune Systems "Apex Controller." The plastic supply tubing from the PMUP is directed to the far right back corner of the sump (**Figures 2, 4J**). To allow for the passage of this tubing and the two wires for the optical sensors into the sump, a small amount has to be cut off of the very back-right corner of the removable coarse filter sieve plate. Zip ties are used to secure the tubing to the far right rear aluminum rack upright support bar (**Figure 4J**). Note that when combined with the Iwaki RODI pump that counters evaporation, water level can be precisely maintained to within 1-2mm inside the sump.

<u>34. Feeding System (output to D5, Figure 5A):</u>

The feeding system was constructed by Dr. Jonathan Henry, UIUC (**Figure 5A-B; Additional File 4, Additional File 5, Additional File 6, Additional File 7, Additional File 8, Additional File 9, Additional File 10, Additional File 11, Additional File 12, Additional File 13, Additional File 14**). The feeding system consists of an Arduino Uno microcontroller (**Figure 5C, Additional File 4, Additional File 5**) that regulates the activity of a custom peristaltic pump (see below, **Additional File 2, Additional File 13, Additional File 14** and **Figure 5B, D).** The microcontroller receives several sensor inputs. These sensors determine 1) the level of food present in the food reservoir via a non-contact capacitance sensor (**Figure 5E,** see **Additional File 3F-G**), 2) whether food has actually been loaded into the Teflon feeding line, and 3) when food is cleared from that line to ensure accurate and complete delivery of the phytoplankton via a pulsed infrared sensor (**Figure 5F, see Additional File 7, Additional File 8, Additional File 9, Additional File 10, Additional File 11, Additional File 12**). The microcontroller also operates two 120 volt AC relays located inside a remote outlet box (**Figure 5C, Additional File 4, Additional File 5**) to 1) activate a magnetic stir plate (used to resuspend the food in the food reservoir 5 mins before feeding; **Figure 5B**) and 2) to activate the aquarium air-pump (used to finally purged residual food from the Teflon feeding line; **Figure 5C, D**). A battery backup real-time clock (RTC) reports the current time and date and lets the microcontroller know when it is time to activate and terminate the feeding cycles. Note that one may wish to reset the correct time on the RTC for any daylight savings time changes. This has to be done using a different Arduino program sketch. The timing of feeding cycles must be programmed into the Arduino microcontroller's sketch. Note that we

have currently set feeding cycles for 24 mins every 2 hours, with 2.8 ml of diluted food being delivered during each feeding (36.6ml/day, equal to equal to 2.24x10^9 cells/ml). Since this food is pre-diluted to a strength of 75% with sterile dH_2O, the actual amount of concentrated undiluted food being delivered is 2.1ml/2 hrs, or 25.2ml/day. A digital hi/low (1/0) output communicates with the Walchem 900 aquarium system controller (signal sent to solid state relay (D5) in the Walchem 900 controller) to tell the aquarium when it is time to activate a feeding cycle and redirect the filter bypass valve. Likewise, a digital hi/low (0/1) pulsed output sends a signal to the peristaltic pump to activate a pre-programmed feeding cycle. These parameters (including the number of steps (volume) and speed (steps per min) must be entered directly into the custom stepper motor controller's memory using its joystick interface (see **Additional File 13, Additional File 14).** Once set, these pumping programs are non-volatile and are stored permanently in memory, to be recalled in the event of a power failure. Thus, there is no need to adjust the feeding system if the power should cycle.

The feeding system microcontroller has an illuminated push button power switch (**Figure 5C**). When the system is on and in a non-feeding (filtering) cycle this light will be green. When the system is on and in a feeding cycle (non-filtering) the light will turn blue. If a fault is detected and an alarm is triggered, the light will be red.

The feeding system's Arduino Uno also has an attached ethernet shield, which can be connected to a computer network. Text messages and/or emails will be sent to predefined users: whenever, 1) the food becomes too low in the food reservoir, 2) when no food is loaded into the Teflon feeding line, and 3) if the food is not completely cleared from the Teflon feeding line.

Please note that the alarm indicator light A3 for feeding cycles on the Walchem 900 controller (actually an indicator of the position of the bypass valve) is only activated when the bypass loop valve is actually in the bypass (feeding) position. This differs from the regular vs. feeding mode indication provided on-line through the V-touch server portal, which depends on the state currently being sent by the feeding controller (to D5). Unless someone has gone into the Walchem controller to change these states, they should be the same. Note that if the power were to be interrupted during a feeding cycle, the system would revert back to a non-feeding (filtering) mode, until the next feeding cycle is triggered.

For the peristaltic pump, we purchased a small pump head and stepper motor and developed our own custom stepper motor microcontroller circuit and program (See **Additional File 2, Additional File 13, Additional File 14**). The pump head accepts a short length of 4mm OD x 2mm ID silicone tubing, which is connected to barbed Luer lock hose adapters, a single Luer lock Y connector and a one-way check valve (Qosina, Cole-Parmer, Vernon Hills, IL). Together, these create a pump to withdraw food from a reservoir and dispense this to the aquarium system (**Figure 5B-D, and Additional File 2**). The food is ultimately passed to the aquarium system through 1/8" Teflon tubing. A special flangeless compression fitting is used to attach the 1/8" Teflon tubing to this valve system (HPLC waste line adapter, HPLC-LL-1/8-KIT; CPLabSafety, San Francisco, CA; **Figure 5D**). This tubing is directed to the left sump compartment through a

hole drilled in the top of the small removable PVC sump cover located directly above the aquarium system main water pump intake (**Figures 2, 4N**). Note that the silicone tubing and other parts (**Figure 5D**) will need to be sanitized and/or replaced periodically. Any problems in the delivery of the food will be detected by the IR food line sensor (**Additional File 9, Additional File 10, Additional File 11, Additional File 12**) and users will be alerted to the problem via email and text messages, and they should correct the issue as soon as possible. Such problems typically involve only one or both of the feeding valves. For cleaning, parts of the feeding system including the silicone tubing, Luer lock connections, supply tube and Teflon feeding line should be removed, rinsed several times with sterile dH_2O and finally cleaned with 2-3 rinsed of 70% ethanol. After cleaning, the parts should be dried and reassembled. Parts should be checked to make sure they are tight. These inexpensive parts should be replaced every 6-12 months or sooner, as needed. At the same time, the food reservoir should be cleaned, autoclaved and refilled with fresh food.

We dilute the food to a 75% concentration with sterile dH_2O. It is important to prime the feeding pump to make sure the food is flowing properly. In any case, users will be alerted when there are problems with food delivery via an email or text message.

Formerly, we tried using a syringe pump to deliver the food, but this required a more elaborate arrangement of parts and was prone to problems involving air bubbles. The use of the peristaltic pump has proven to be very reliable and precise in terms of the volume of food that is delivered. Furthermore, this setup is less costly. One note of caution, however, as the silicone tubing wears out the volume delivered may change. Peristaltic pumps that use stepper motors are ideal as they produce high torque with precise degrees of rotation. The same input from the main feeding controller is used to trigger a preset number of steps (at the preset speed (rpms)) to deliver the correct, pre-calibrated volume of food. The volume of food will depend on the diameter of the silicone tubing used in the peristaltic pump. Therefore, one will have to calibrate the volume and number of steps needed to deliver the desired amount of food. The stepper motor has 200 steps per revolution and is rated for no more than 60RPM. We generally set the speed to 50RPM.

35. General Leaks:

Water leaks may form at any time. It may be prudent to place the system in **standby** mode to inspect and repair these leaks. If these leaks occur at various PVC fittings or unions they need to be inspected and tightened. Look for any cracks, and use the appropriate tools to service these parts (see **Figure S1**). Generally, these are to be hand-tightened, but a little coaxing with a wrench may be required. Silicone rubber kitchen jar pads are useful for getting a slip-proof grip for loosening or hand-tightening these parts. Alternatively, one can use the Channel Lock oil filter/PVC wrench (Channellock, Inc., Meadville, PA) to tighten or loosen these fittings. Excessive force should be avoided, so as not to damage the plumbing. Over-tightening these parts may cause them to crack or seize.

36. Dripping Valves:

Occasionally, a ball valve may drip when it is supposed to be shut off, or a valve may not provide sufficient flow, and these valves may need to be tightened or cleaned. To service the larger ball valves with red handles, one must use an adjustable wrench and a special ½" valve seal carrier key, TSK 1-005, to tighten the valve seat (**Figure S1**). This is done by loosening the union and accessing the seat through the main valve opening where it can be tightened using the key (follow the manufacturer's instructions). For the smaller ball valves that serve each tank (with the blue handles), no adjustments can be made. One would simply have to replace those valves. This requires that one loosen the entire valve manifold at its union so you can rotate the manifold downward, which will then allow the valve to be unscrewed and replaced. Teflon tape is used to help seal these small valve threads.

37. Alarms:

Notifications are sent to users whenever conditions fall outside the predefined ranges. Hi, Lo, HiHi and LoLo alarm limits may be specified for all measurable conditions. Note that these ranges are specified independently of those actually used to control those parameters (e.g., conductivity, temperature, pH, etc. See Walchem 900 instruction manual). Whenever an alarm condition is activated, Alarm light A8 will be illuminated and a text message and email will be sent to the users. Messages will also be sent whenever alarms are cleared. Note that unlike the feeding controller, these alarm notifications are only sent once and are not repeated. Note that deadbands for the inputs and outputs can be adjusted, as needed. For inputs, these deadbands set the values (limits) for when alarms will be sounded and when they will be cleared. Likewise, for outputs, these deadbands set the (values) limits for actuating any corrections (e.g., when the heater is turned off) and when those corrections will be stopped (e.g., when the heater is turned off). Note also for the outputs one also has to specify the direction for these corrections (e.g., whether they will be forced higher or lower, see settings below).

38. Inoculating the System:

New systems require establishment of the (bacterial) biological filter before animals can safely be introduced. When first inoculating the system with bacteria, inactivate the UV lamp (HOA OFF mode) the skimmer (HOA OFF mode) and the feeding bypass valve (HOA OFF mode) by entering these settings in HOA. One can add Seed bacteria (Aquavitro, Seachem, Madison, GA) or Dr. Tim's One and Only Live Nitrifying Bacteria (Dr. Tim's Aquatics, Ltd., Moorpark, CA) to the sump following the manufacturers' instructions, and allow the system to circulate in filter mode. In addition, or alternatively, one can add some filtered sea water from an already established system to inoculate the biofilters (in our case we added 1 liter of filtered water, in addition to Dr. Tim's solution). To prevent any larger organisms, embryos or larvae from being transferred to the new system, we filtered this water by gravity using number 1 Whatman filter paper, which has a porosity of about 11µm. We then added ammonium chloride (9ml of 50mg/ml solution per 40 gallons, or a final concentration of 2ppm in the system) for the bacteria and monitor ammonium, nitrite and nitrate levels. To cycle the aquarium using ammonia, add enough of a 50mg/ml solution of ammonium chloride ($NH_4^+Cl^-$, VWR, 0621-500g, Radnor, PA) to reach a concentration of 2mg/L (ppm). We added 9ml of 50mg/ml ammonium chloride solution per 40 gallons, to a final concentration of 2ppm in the system. Continue dosing with ammonium chloride every two to three days to maintain ammonium levels at 2ppm

until they begin to drop and nitrite levels start to rise. During the initial cycling of the aquarium, levels of ammonia and nitrite should be measured every 2-3 days. To monitor the levels of ammonia, nitrites and nitrates, we use test kits produced by API Marine (Mars Fishcare North America, Chalfont, PA). During this process, occasionally allow some water to circulate through the feeding bypass loop by activating the motorized bypass valve for a few mins (HOA, Hand Mode). Within two weeks, levels of ammonia should start to decrease (<1ppm), and nitrite levels will rise. The appearance of nitrite in the system indicates the presence of ammonia-oxidizing bacteria. Reduced doses of ammonia can continue to be added to the system every few days, being careful not to exceed 5ppm. Higher levels (>5ppm) of ammonia and nitrites will inhibit the growth of nitrite-oxidizing bacteria. Nitrite levels will slowly increase, and should spike around 2-3 weeks after the initial addition of ammonia. Continue to monitor ammonia and nitrite levels in the system, adding low doses of ammonia every few days, keeping nitrite levels below 5ppm. Continue until nitrite levels drop to 0ppm. The inoculation procedure should only have to be done once when first setting up the system.

In our own experience, we observed no change in the highly elevated levels of ammonia and no detectable levels of nitrite and nitrate nine days after we added Dr. Tim's. Being impatient, we began to add SEED over the next 8 days following the manufacturer's instructions. By the 7th day we started to detect nitrites, and four days later there was no detectable ammonia, and detectable (high) levels of nitrites and nitrates. At this time a very hardy animal (e.g., a blue damsel fish) was added to the system. The addition of an animal provides a small amount of ammonia to support some of the bacteria in the system. If not using an animal, continue to add ammonia every few days, until both ammonia and nitrite levels recover to zero. When the ammonium and nitrite levels have finally been reduced to zero, it is safe to begin adding other live animals. It can take several weeks for this to occur, and one must have patience. Nitrate levels may be very high after this procedure, though the system is designed to reduce these levels over time, which was eventually our experience. If nitrate levels remain high, one should perform water changes. Up to a quarter of the sea water may be exchanged each time with freshly mixed sea water to reduce nitrate levels; though, in our own experience, we observed these to fall to 0ppm over the course of several weeks without any water changes. In our system, with a relatively light animal load, we observed only 5ppm nitrate after 7 months of use, and have not yet needed to perform any water changes.

39. Replacing the Main Pump (Figures 1L, 2):

The pump will last a long time, but eventually will need replacement. Generally, the first parts to fail are the bearings, and this will be noticeable by a change in sound, which will become louder and eventually become annoying.

To replace the main pump (**Figure 1L**), first shut **off** the system and disconnect the power. Wait for about one minute for all the sea water to drain back to the sump. Close valves #4, 1, 2, 6 and 5 (see **Figure 2; Figure S2**). Drain sea water using valve #3 with the push on female quick connector and its short piece of tubing. (Use a large clean beaker to collect the sea water, which will be returned to the system.) Purge some air and additional sea water (via valve #3) from each filter by pushing the purge valves on the top back sides of the filter housings.

Disconnect the power line to the motor within the Walchem 900 controller and free the power cord. Be sure to observe the proper connections for future reference and to properly reinstall the motor. Place absorbent towels beneath the PVC unions for valve #4 and over the motor under/around the union for the motorized ball valve. Loosen the PVC unions connecting the main pump to valve #4 (**Figure S2A**) and finally the motorized bypass ball valve (**Figure 5I, Figure S2H**). Loosen/remove the bolts that attach the motor to the bottom shelf (**Figure 1L)**. These bolts are held on using simple wing nuts. Keep track of the locations of the nuts, bolts and rubber motor mounts. Before removing the pump, note that it is very important to have something ready (e.g., the new replacement motor/impeller, to support the heavy motorized ball valve, as well as the free disconnected unions for valves #1 and 4! An extra set of hands is useful. Install the new pump motor by reversing these steps. Reconnect the motor's power cord. Return any collected sea water back to the sump. Restore system power, while carefully inspecting for any leaks.

40. Additional Warnings and Advice:

When done servicing the system, be sure to close and open the appropriate valves and turn the system back on!

Warning!! Hands must be washed in tap water only, before working with the system. No soap or detergents should ever be allowed to enter the system, as these substances are toxic to the animals. Likewise, no perfume or hand lotion should be used by operators of the system!

The following plastics are compatible with sea water, including: Nylon, PVC, Teflon, Acetyl (Delrin), polyproplyene. Titanium and glass are also fairly impervious to the effects of sea water. Aluminum and stainless steel will eventually corrode.

41. References:

Henry JQ, Lesoway MP, and Perry KJ, (2020). An Automated Aquatic Rack System for Rearing Marine Invertebrates. BMC Biol, *In Press*.

Henry JQ, Lesoway MP, Perry KJ (2020) HenryLabUIUC (Henry Lab) Github. https://github.com/HenryLabUIUC.

Henry JQ, Lesoway MP, Perry KJ, Osborne CC, Shankland M, and Lyons DC. (2017). Beyond the sea: *Crepidula atrasolea* as a spiralian model system. Int J Dev Biol, 61: 479-493.

Walchem (2019) W900 Series Water Treatment Controller Instruction Manual. https://www.walchem.com/Literature/Controllers/W900/180686_W900%20Manual_E.pdf.

42. Master List of all System Controller Settings:

The following is a list of the Walchem 900 settings used for our aquatic system.

42.1. Inputs:

Cond (S11)
LoLo Alarm (mS/cm): **51.4**
Low Alarm (mS/cm): **51.9**
High Alarm (mS/cm): **54.5**
HiHi Alarm (mS/cm): **54.7**
Deadband (mS/cm): **0.1**
Reset Calibration Values: **Confirm**
Cal Required Alarm (days): **0**
Smoothing Factor (%): **0**
Cell Constant (1/cm): **10.000**
Cable Length (m): **1.00**
Gauge: **22 awg/0.35 mm2**
Default Temp (°C): **25.0**
Name: **Cond**
Alarm suppression: **D1, D3**
Temp Compensation: **NaCl**
Units: **mS/cm**
Type: **Contacting Cond**

Temp(S12)
LoLo Alarm (°C): **-20.0**
Low Alarm (°C): **-20.0**
High Alarm (°C): **260.0**
HiHi Alarm (°C): **260.0**
Deadband (°C): **0.5**
Reset Calibration Values: **Confirm**
Cal Required Alarm (days): **0**
Smoothing Factor (%): **0**
Name: Temp
Alarm Suppression: **D1, D3**
Element: **RTD 1000ohms**

pH
LoLo Alarm: **7.70**
Low Alarm: **7.8**
High Alarm: **8.3**
HiHi Alarm: **8.3**
Deadband: **0.10**

Smoothing Factor (%): **0**
Reset Calibration Values: **Confirm**
Cal Required Alarm (days**): 0**
Buffers: **Manual Entry**
Default Temp (°C): **25.0**
Cable Length (m): **1.00**
Gauge: **22 awg/0.35mm2**
Electrode: **Glass**
Alarm Suppression: **D1, D3**
Name: **pH**

Temp(S14)
LoLo Alarm (°C): **23.0**
Low Alarm (°C): **25.0**
High Alarm (°C): **26.7**
HiHi Alarm (°C): **28.5**
Deadband (°C): **0.5**
Reset Calibration Values: **Confirm**
Cal Required Alarm (days): **0**
Smoothing Factor (%): **0**
Name: Temp
Alarm Suppression: **D1, D3**
Element: **RTD 1000ohms**

Sump Level (S21)
LoLo Alarm (°C): **14.00**
Low Alarm (°C): **28.00**
High Alarm (°C): **30.50**
HiHi Alarm (°C): **31.50**
Deadband (°C): **0.5**
Reset Calibration Values: **Confirm**
Cal Required Alarm (days): **0**
4 mA Value (cm): **0.00**
20 mA Value (cm): **38.00**
Units: **cm**
Smoothing Factor (%): **0**
Transmitter: **2 Wire Loop**
Name: **Sump Level**
Alarm Suppression: **D1**
Type: **AI Monitor**

Unassigned (S22)
Type: **No Sensor**

Sys On/Off (D1)
Open Message: **System Off**
Closed Message: **System On**
Interlock: **When Open**
Alarm: **Disabled**
Total Time: **When Open**
Reset Time Total: **Confirm**
Name: **Sys On/Off**
Type: **DI State**

Flowmeter (D2)
LoLo Alarm (l/m): **3.80**
Low Alarm (l/m): **36.00**
Deadband (l/m): **1.0**
Totalizer Alarm: **0**
Reset Flow Total: **Confirm**
Set Flow Total: **0**
Scheduled Reset: **Disabled**
K Factor (pulse/l): **68.68**
Flow Units: **l**
Rate Units: **min**
Smoothing Factor (%): **90**
Name: **Flowmeter**
Type: **Paddlewheel FM**

Sensors (D3)
Open Message: **NO FLOW**
Closed Message: **Have FLOW**
Interlock: **When Open**
Alarm: **When Open**
Total Time: **When Closed**
Reset Time Total: **Confirm**
Name: **Sensors**
Type: **DI State**

Unassigned (D4)
Type: **No Input**

Feed Mode (D5)
Open Message: **Filtering**
Closed Message: **Feeding**
Interlock: **When Closed**
Alarm: **Disabled**
Total Time: **When Open**

Reset Time Total: **Confirm**
Name: **Feed Mode**
Type: **DI State**

Unassigned (D6)
Type: **No Input**

Unassigned (D7)
Type: **No Input**

Unassigned (D8)
Type: **No Input**

Unassigned (D9)
Type: **No Input**

Unassigned (D10)
Type: **No Input**

Unassigned (D11)
Type: **No Input**

Unassigned (D12)
Type: **No Input**

Raw Value (V1)
LoLo Alarm (mA): **0.00**
Low Alarm (mA): **0.00**
High Alarm (mA): **21.00**
HiHi Alarm (mA): **21.00**
Deadband (mA): **1.05**
Input: **Sump Level (S21)**
Smoothing Factor (%): **0**
Name: Raw Value
Alarm Suppression: **None checked**
Type: **Raw Value**

Unassigned (V2)
Type: **Not Used**

Unassigned (V3)
Type: **Not Used**

Unassigned (V4)

Type: **Not Used**

Unassigned (V5)
Type: **Not Used**

Unassigned (V6)
Type: **Not Used**

Unassigned (V7)
Type: **Not Used**

Unassigned (V8)
Type: **Not Used**

42.2. Outputs:

Pump (R1)
HOA Setting: **Auto**
Setpoint (cm): **20.00**
Deadband (cm): **7.00**
Duty Cycle Period (MM:SS**): 00:00**
Duty Cycle: **100.0**
On Delay Time (HH:MM:SS): **00:00:00**
Off Delay Time (HH:MM:SS): **00:00:00**
Output Time (HH:MM:SS): **00:00:00**
Reset Output Timeout: **Confirm**
Min Rely Cycle (sec): **0**
Hand Time Limit (HH:MM:SS): **00:00:00**
Reset Time Total: **Confirm**
Input: **Sump Level (S21)**
Direction: **Force Lower**
Name: **Pump**
Interlock Channels: **D1**
Activate With Channels: **None Selected**
Mode: **On/Off**

UV (R2)
HOA Setting: **Off**
Setpoint (l/m): **4.00**
Deadband (l/m): **1.00**
Duty Cycle Period (MM:SS**): 00:00**
Duty Cycle: **100.0**
On Delay Time (HH:MM:SS): **00:00:00**
Off Delay Time (HH:MM:SS): **00:00:00**

Output Time (HH:MM:SS): **00:00:00**
Reset Output Timeout: **Confirm**
Min Rely Cycle (sec): **0**
Hand Time Limit (HH:MM:SS): **00:10:00**
Reset Time Total: **Confirm**
Input: **Flow Rate (D2)**
Direction: **Force Lower**
Name: **UV**
Interlock Channels: **D1**
Activate With Channels: **None Checked**
Mode: **On/Off**

Bypass (R3)
HOA Setting: **Auto**
On Delay Time (HH:MM:SS): **00:00:00**
Off Delay Time (HH:MM:SS): **00:00:00**
Output Time (HH:MM:SS): **00:00:00**
Min Rely Cycle (sec): **0**
Hand Time Limit (HH:MM:SS): **00:00:00**
Name: **Bypass**
Interlock Channels: **D1**
Activate With Channels: **D5**
Mode: **Manual Control**

RO Pump (R4)
HOA Setting: **Auto**
Setpoint (mS/cm): **53.00**
Deadband (mS/cm): **0.1**
Duty Cycle Period (MM:SS**): 00:00**
Duty Cycle: **100.0**
On Delay Time (HH:MM:SS): **00:00:00**
Off Delay Time (HH:MM:SS): **00:00:00**
Output Time (HH:MM:SS): **00:00:00**
Reset Output Timeout: **Confirm**
Min Rely Cycle (sec): **0**
Hand Time Limit (HH:MM:SS): **00:10:00**
Reset Time Total: **Confirm**
Input: **Cond (S11)**
Direction: **Force Lower**
Name: **RO Pump**
Interlock Channels: **D1, D3**
Activate With Channels: **None Checked**
Mode: **On/Off**

pH Pump (R5)
HOA Setting: **Off**
Setpoint: **7.80**
Deadband (cm): **0.10**
Duty Cycle Period (MM:SS**): 00:00**
Duty Cycle: **100.0**
On Delay Time (HH:MM:SS): **00:00:00**
Off Delay Time (HH:MM:SS): **00:00:00**
Output Time (HH:MM:SS): **00:00:00**
Reset Output Timeout: **Confirm**
Min Rely Cycle (sec): **0**
Hand Time Limit (HH:MM:SS): **00:05:00**
Reset Time Total: **Confirm**
Input: **pH (S13)**
Direction: **Force Higher**
Name: **pH Pump**
Interlock Channels: **D1, D3**
Activate With Channels: **None Checked**
Mode: **On/Off**

Heater (R6)
HOA Setting: **Auto**
Setpoint (°C): **25.7**
Deadband (°C): **0.50**
Duty Cycle Period (MM:SS**): 00:00**
Duty Cycle: **100.0**
On Delay Time (HH:MM:SS): **00:00:00**
Off Delay Time (HH:MM:SS): **00:00:00**
Output Time (HH:MM:SS): **00:00:00**
Reset Output Timeout: **Confirm**
Min Rely Cycle (sec): **0**
Hand Time Limit (HH:MM:SS): **00:05:00**
Reset Time Total: **Confirm**
Input: **Temp (S14)**
Direction: **Force Higher**
Name: **Heater**
Interlock Channels: **D1, D3**
Activate With Channels: **Non Checked**
Mode: **On/Off**

On/Off (R7)
HOA Setting: **Auto**
Setpoint (cm): **6.00**
Deadband (cm): **0.40**

Duty Cycle Period (MM:SS**): 00:00**
Duty Cycle: **100.0**
On Delay Time (HH:MM:SS): **00:00:00**
Off Delay Time (HH:MM:SS): **00:00:00**
Output Time (HH:MM:SS): **00:00:00**
Reset Output Timeout: **Confirm**
Min Rely Cycle (sec): **0**
Hand Time Limit (HH:MM:SS): **00:10:00**
Reset Time Total: **Confirm**
Input: **Sump Level (S21)**
Direction: **Force Higher**
Name: **On/Off**
Interlock Channels: **None Checked**
Activate With Channels: **D5**
Mode: **On/Off**

Alarm (R8)
HOA Setting: **Auto**
Output: **Normally Open**
Min Relay Cycle (sec): **0**
Hand Time Limit (HH:MM:SS): **00:00:00**
Reset Time Total: **Confirm**
Name: **Alarm**
Select Alarms: **Select Alarms**
Interlock Channels: **None Checked**
Activate With Channels: **None Checked**
Alarm Mode: **Selected Alarms**
Mode: **Alarm Output**

Unassigned (C1)
Mode: **Unassigned**

Unassigned (C2)
Mode: **Unassigned**

Unassigned (C3)
Mode: **Unassigned**

Unassigned (C4)
Mode: **Unassigned**

Unassigned (C5)
Mode: **Unassigned**

Unassigned (C6)
Mode: **Unassigned**

Unassigned (C7)
Mode: **Unassigned**

Unassigned (C8)
Mode: **Unassigned**

Config:

Global Settings
Date(YYYY-Mmm-DD):
Time(HH:MM:SS):
Name:
Location:
Global Units: **Metric**
Temperature Units: **°C**
Alarm Delay (MM:SS): **00:30**
HVAC Modes: **disabled**
Language: **English**

Ethernet Settings
TCP Timeout (sec): **1**
VTouch Status: **Enabled**
Live Connect Status: **Enabled**
Update Period (min): **15**
Reply Timeout (sec): **15**
DHCP Setting: **Enabled**

Email Report Settings
Report #1: **Alarm**
Report #2: **Data Log**
Report #3: **Summary**
Report #4: **Data Log**
Email Addresses: **7**
SMTP Sever: **express-smtpo.cites.illinois.edu**
SMTP Port: **25**
From Address: **no-reply@illinois.edu**
Email Server: **SMTP**

Display Settings
Home 1: **Sys (ON/Off (D1)**
Home 2: **pH (S13)**

Home 3: **Temp (S14)**
Home 4: **Cond (S11)**
Home 5: **Flowrate (D2)**
Home 6: **Sump Level (S21)**
Home 7: **Sensors (D3)**
Home 8: **feed Mode (D5)**
Key Beep: **Disabled**

File Utilities
Data Log Range: **1 Day**
Log Frequency: **10 seconds**
Export Data: **Download**
Export Event Log: **Download**
Export System Log: **Download**

43. Micro Controller Details:

Settings for the feeding system and peristaltic pump are explained in the comments provided in the relevant program files (see **Additional File 5** and **Additional File 14**).

<u>44. Supplemental Figure Legends:</u>
(Note, Main Figures 1-5 are published in the accompanying manuscript, Henry *et al*., 2020)

Figure S1. This image shows the basic tools needed to service the system. aw, adjustable wrenches; co, handheld conductivity meter; dc, drain quick-connector for activating main drain valve. (Note short clear tubing attached to this part. In this configuration the connector is only used to drain a small amount of residual sea water from the system, after it has been turned off for servicing). fu, spare fuses; gc, short graduated cylinder for calibrating the pH and conductivity probes; fw, adjustable filter wrench for the PVC unions; ir, handheld infrared thermometer; pH, handheld pH meter; sg, silicone grease for lubricating O-rings; sw, large and small plastic spanner wrenches for the filter bowls; va, ball valve adjustment tool; sd, assorted straight blade and Phillips screwdrivers; sp, silicone grip pads; tt Teflon tape (for sealing threaded parts).

Figure S2. Valves that control water flow. **A.** Intake valve for the main water pump (v4) valve is shown open for normal operation. The PVC Union (un) that must be loosened to change out the water pump is also visible. **B.** Main outflow valve (v1), shown open for normal operation. Valve (v2) supplying the red sensor side loop tubing is shown fully open. Outflow water pressure gauge (pg) is also visible. **C.** Main drain valve (v3). Needle valve (v6) supplies water to the bioreactor. Check valve (see I, below) is visible at back nbext to the carbon fiulter. **D.** Bypass valve (v5) is shown closed, just next to the flow meter. This valve can be opened somewhat to divert water back to the sump, as needed (see SOP, **Section 30: The Main Bypass Valve**). **E.** Accessory filler valve (v7) supplies water to the blue tubing for removing water from the system. This valve is shown closed. One of the manifold valves (vF) is seen in the back that supplies water to the manifold serving the bottom (F) row of tanks. **F.** Manifold valve supplying water to the D row of tanks. This valve is shown partially open (45 degrees). **G.** Tank valves supplying water to individual tanks. These valves are shown partially open (45 degrees). **H.** Motorized bypass valve (bv). During a feeding cycle diversion of water away from the filters, to the bypass loop is indicated by white color showing in the window. **I.** Check valve (cv) that prevents water from passing backwards through the canister filters during feeding. **J.** Drain quick-connector that opens up the main drain valve (v3) after it is pushed into place. A small push-button on the side disconnects this part and shuts the spring loaded valve. cf. carbon filter; cv, check valve; dc, main drain valve quick-connector; fm, flow meter; mn, manifold; pg, pressure gauge; sm, sump; tv, tank valves; un, union; v1, main effluent valve; v2, Sensor side loop valve; v3, main drain valve; v4, main water pump intake valve; v5, bypass valve; v6, needle valve for bioreactor; v7; accessory filler valve; vD, manifold valve for row D; vF, manifold valve for row F; wd, window; wp, water pump.

Figure S3. Replacing the mechanical filters. **A.** The clear plastic Origami cover has been removed exposing the large opening over the right compartment of the sump and the coarse filter pad. Note cellophane that is draped over the slotted drain diverter to limit splashing and to direct the drain water through the coarse filter pad. **B.** Removal of the coarse filter pad. This pad acts like a large sponge and retains a lot of sea water. Remove the pad slowly and tilt it upwards to allow residual sea water to drain back into the sump before discarding the pad. **C.** After the old

pad has been removed, the perforated sieve plate is exposed, which supports the filter pad. A new pad is placed on top of the sieve plate, and the cellophane and Origami covers are replaced. If access is needed to the right compartment of the sump, the sieve plate can be removed by lifting up the top lid of the sump about an inch or two and sliding the plate out towards the viewer. The slotted drain divertor will need to be removed to lift the top lid. **D.** Preparation for replacement of the pleated mechanical filter cartridge. Note white coloration of the new cartridge compared to the old cartridge inside the filter bowl. A bucket is used to collect sea water that may spill during replacement. **E.** To help drain some water from the filter, air is allowed to enter the bowl by pressing the red purge valve **F.** The filter bowl is loosened using the filter wrench, as shown. Continue to unscrew the bowl using one's hands. Pull the old cartridge out of the bowl and replace it with the new one. Screw the filter bowl back in place and tighten it using the filter wrench, as needed. bk, bucket; cl, cellophane; fp, filter pad; fw, filter wrench; mf, mechanical filter; pf, pleated filter; pv, purge valve; sd, slotted drain diverter; sm, sump; sp, sieve plate; tl, top lid of sump.

Figure S4. Calibrating the pH and conductivity sensors. **A**. Prepare for calibration by turning off Valve (#2). **B**. Loosening the large gray union that retains the pH probe. A filter wrench can be used to help loosen stuborn fittings. **C**. Removal of the pH probe from the sensor side loop for calibration. Yellow arrows show the direction of water flow through the sensor side loop. Note that both ends of the pH sensor probe and its internal stainless steel temperature sensor should be oriented (rotated) so that they are exposed to the on-coming flow of sea water. **D**. Calibration of the pH probe using standard calibration solutions. Follow the on-screen instructions after entering the appropriate Walchem 900 calibration menu. **E**. Two adjustable wrenches are used to loosen the conductivity sensor. Only the top black plastic fitting is to be loosened. The stainless steel probe simply slides out. Unlike the pH probe, there is no specific orientation for the conductivity probe, as long as it is seated deep enough to that a sufficient amount of sea water flows through the tip. Calibration of the conductivity probe is similar to that of the pH probe using a single standard calibration solution. Follow the on-screen instructions after entering the appropriate Walchem 900 calibration menu. cd, conductivity sensor; cs, pH 7 calibration solution; ft, float switch; pH, pH sensor, tm, temperature sensor; un, union; v2, sensor side loop valve.

44. Table of Contents:

Section Number	Section Heading	Page Number
1.	Basic Control of the Aquatic System	1
2.	Tools for Servicing the System	1
3.	The Rack Shelving Unit	2
4.	Water Flow	2
4.1.	*Water Flow During Normal Operation*	2
4.2.	*Water Flow During Feeding*	3
5.	Individual Tanks	5
6.	RO and pH Reservoirs	6
7.	Adjustment and Priming of the RO and pH Pumps	6
8.	Servicing the Sump Pad Filter	7
9.	Servicing the Protein Skimmer	7
10.	Servicing the Cartridge Filters, General Instructions	9
10.1.	*Servicing the Pleated Mechanical Filter*	9
10.2.	*Servicing the Biological Filter*	10
10.3.	*Servicing the Activated Carbon Filter*	10
10.4.	*Servicing the Nitrate Bio-Reactor*	10
11.	Monitoring Water Quality	12
12.	Servicing the UV Lamp and Quartz Sleeve	13
13.	Aquarium System Temperature Regulation	14
14.	External Chiller System	15
15.	Servicing the Sensors	16
15.1.	*Calibration of the pH probe*	17
15.2.	*Calibration of the Conductivity Sensor*	17
15.3.	*Potential Problems after Calibration*	18
16.	Double Checking the Sensors	19
17.	Sea Water	19
18.	Bags of Crushed Coral/Shell	19
19.	Accessing the Right Sump Compartment	19
20.	Main Pump Inlet	19
21.	Pump Impeller Cavitation	20
22.	Drain Valve and Accessory Water Filler Tap	20
23.	Automatic Mechanical Float Filler Valve	21
24.	Float Switch	21
25.	Servicing the Paddle Wheel Flow Sensor	21
26.	Calibration of the Echopod: Sump Water Level Sensor	22
27.	Preventing Evaporation	22
28.	Sump Covers	22
29.	Salt Creep	22
30.	Main Bypass Valve	23

31. Metal Components Exposed to Sea Water (Risks of Corrosion) 23
32. Reverse Flow Check Valve 23
33. Automatic Sea Water Top-Off System 23
34. Feeding System 25
35. General Leaks 27
36. Dripping Valves 27
37. Alarms 28
38. Inoculating the System 28
39. Replacing the Main Pump 29
40. Additional Warnings and Advice 30
41. References 30
42. Master List of all System Controller Settings 31
42.1. Inputs 31
42.2. Outputs 35
43. Micro Controller Details 40
44. Supplemental Figure Legends 1-4 41
45. Table of Contents 43
