# Supplementary material for: An automated aquatic rack system for rearing marine invertebrates
Source: BMC Biol. 2020 May 4;18:46. doi: 10.1186/s12915-020-00772-w (PMC7199361; doi:10.1186/s12915-020-00772-w)
Supplement: Supplementary file 2 — Additional file 2. List of parts and suppliers for components used in the aquatic rack system. Note: Additional files 3, 4, 5, 6, 7, 8, 9, 10, 11, 12, 13, 14, 15, 16 are available at Github [41]. [file 12915_2020_772_MOESM2_ESM.pdf]

List of Parts and Suppliers for Components used in the Aquatic Rack System: (Henry Lab, UIUC)

Additional File 2

Jonathan Q. Henry, Maryna P. Lesoway, Kimberly J. Perry

Department of Cell & Developmental Biology
University of Illinois
601 South Goodwin Ave.
Urbana, IL 61801

Ver. March 16, 2020

Figure notations (Figures 1-5) refer to those included in Henry et al. (2020), and additional figures (Figures S1-S4 are supplemental figures included with Additional File 1, SOP). Other file references include correspondingly numbered Additional Files that also accompany Henry et al., (2020), which are all available at https://github.com/HenryLabUIUC/Automated-Aquatic-System.

Feeding system peristaltic food pump, and associated parts (to deliver the food), Figure 4A-D:

1 x 120 Can beverage cooler (mini-refrigerator), stainless steel with glass door (model no. DBC120BLS, Danby Products, Inc., Guelph, Ontario, Canada). **Figure 5A**.
Used to maintain the food and feeding pump equipment at 4°C. Note that a 1”-3/8” dia. hole has to be drilled through the back side wall, just to the left side above the compressor compartment to permit access for various power cords, the airline and control cables. This hole can be drilled straight down (in a direction towards the floor), using a hole saw, starting from the inside, through the plastic liner, insulation and finally the sheet metal. Exposed sharp sheet metal edges must be covered with grommets to prevent cutting of the cords and any shock hazard. Once the cables are routed, the hole should be packed with foam insulation. An additional 1/8” dia. hole has to be drilled though the left or right side of the cabinet (plastic liner, insulation and sheet metal) to allow passage of the Teflon food line. WARNING! The location of this small hole is critical, so as not to puncture the cooling and heat exchange coils. On this particular model it is safe to drill this tiny hole either on the left or right side (3-1/4” down from the top surface of the outer cabinet and 5-1/4” back from the front surface of the outer cabinet (not including the door)).

1 x Peristaltic pump with stepper motor (Peristaltic pump with 42 stepper motor dosing tubing hose pump small flow 0-160ml/min (with 42 motor), INZOK, https://www.amazon.com/Peristaltic-Stepper-Dosing-Tubing-0-160ML/dp/B07PYR2P7K?th=1) see **Figure 5D**.

Used as a precise means of delivering the food. Pumping speed (steps per min. and volume (i.e., number of steps) are entered directly into the peristaltic pump controller. Note that even though the motor is listed as a “42 stepper motor,” it is supplied as a 1.8 degrees per step or 200 steps per revolution stepper motor, which is ideal and permits fine volume control.

Silicone tubing (4mm OD x 2mm ID, or similar). Various sources. (e.g., part no. EW-96201-33, Ultra Platinum Silicone tubing, 5/64 ID x 9/64 OD, 25 feet part no. EW-96201-33, Cole Parmer, Vernon Hills, IL).
One piece is supplied with the peristaltic pump, but replacement tubing will be required. High quality tubing lasts for a very long time, but eventually it will become stretched and its elasticity will change as it wears out. This can lead to gradual changes in the volume that is being delivered. Six inches of tubing is used for the pump. See note about tubing connections, below.

1 x Luer lock Y connector with spin lock (part no. 90190, Qosina, Ronkonkoma, NY).
Used to assemble the food pump and to inject air into the feeding line. This part has two female Luer lock fill ports and a single male Luer lock connector with a spin lock.

1 x HPLC waste line adapter (part no. HPLC-LL-1/8-KIT; CPLabSafety, San Francisco, CA).
Used as part of the food pump to connect the Teflon feeding line to very end of the Y connector.

PTFE, Teflon tubing (feeding line; 6-8 feet long, as needed) 1/16” ID, 1/8” OD. (part no. 53XL53, Grainger Industrial Supply, Alsip, IL)
Used as part of the food pump to deliver food to the sump compartment. Length should be kept to a minimum.

2 x 1/8” Barbed to male Luer lock connectors. (part no. 11525, Qosina, Ronkonkoma, NY)
Used to attach the silicone tubing to the Luer lock Y connector and the airline tubing to the Luer lock one-way check valve, listed below. Note that a fine wire may be used to prevent the silicone tubing from slipping off the barbed connector by twisting this tightly around the tubing just below the flared, barbed end of the connector (like a fine twist tie). Do not over-tighten this wire, as it could cut through the silicone tubing.

1 x 1/8” Barbed to female Luer lock connector. (part no. 11524, Qosina, Ronkonkoma, NY)
Used to attach the silicone tubing to the reservoir tubing. Note that a fine wire may be used to prevent the silicone tubing from slipping off the barbed connector by twisting this tightly around the tubing just below the flared, barbed end of the connector (like a fine twist tie). Do not over-tighten this wire, as it could cut through the silicone tubing.

1 x Luer lock tubing (900 SPI 3/16 OD x 48” long, part no. 30526-28 Cole Parmer, Vernon Hills, IL).
Used to draw food from the reservoir. Has both male and female ends. Cut off the female end, and use only the remaining tubing with the male end. Trim this tubing as short as possible so that the cut end just reaches the bottom of the reservoir.

1 x Small aluminum box (BUD Industries, Inc., part number CU-234)

Used to mount the peristaltic pump. A square opening must be machined in the lid of the box with screw holes for mounting the peristaltic pump. Another hole must be drilled to accommodate the cable gland.

1 x Water proof cable gland for cable, PG7 size. Various suppliers, (https://www.amazon.com/Connectors-Plastic-Protectors-Waterproof-Adjustable/dp/B07RHJM435/ref=sr_1_9?keywords=cable+glands&qid=1572968857&sr=8-9) Used to secure the cable and prevent water incursion into the aluminum project box.

1 x 1000 ml screw top Pyrex bottle (part no. 1395, Corning, Germany). **Figure 5E**.
Used as the food reservoir. A hole can be drilled in the screw cap to accept the trimmed Luer lock tubing.

1 x Teflon stir bar. 1" long. (part no. 1451351, Thermo Fisher Scientific,)
Used to resuspend the food.

1 x small 5" x 7" magnetic stir plate (part no. CLS6795410D. Corning, Tewksbury, MA). **Figure 5B**.
Used to resuspend the food.

1 x Machined ½" diameter, by 9 inches long aluminum rod and adjustable bracket. Bar has threaded end (1/4" – 20). Used to hold the capacitive sensor that detects the presence of food in the food reservoir. Adjustable bracket has a clamping screw to secure its position (height) on the bar. (These parts mount directly onto the stir plate, which has a threaded hole on the back. (See **Additional File 3F-H**, **Figure 5E)**.

Feeding system air injection parts (to purge the feeding line), Figure 5C,D:

1 x Air Pump (Uniclife part no. UL40, 4.0 watts, 4.0 L/min, 0.016 MPa pressure, dual twin outlet air pump, or equivalent, Uniclife, https://www.amazon.com/Uniclife-Aquarium-Outlets-Accessories-Adjustable/dp/B01EBXI7PG/ref=zg_bs_2975471011_2?_encoding=UTF8&psc=1&refRID=HHXH8MKDCFSMQQ7KKVQ7). **Figure 5C**.
Used to purge the feeding line of residual food. (Use both pump channels joined with a barbed Y connector for maximum air pressure needed to purge the Teflon feeding line).

1 x Clear aquarium airline tubing (approx., 3/16" OD x 6 feet long).
Used to supply air from the air pump to purge the feeding line. It is attached to a barbed Luer lock connector (see above). (This tubing is supplied as two pieces with the Uniclife air pump)

1 x Barbed dual-ended straight connector.
Used to connect the two airline tubing pieces together (supplied with the Uniclife air pump)

1 x Barbed Y connector.

Used to connect both air pump, air channels together to increase air pressure (supplied with the Uniclife air pump. Cut two short 2 inch long pieces off of the clear airline tubing to connect this Y connector to the pump outlets)

1 x Luer lock one-way check valve, female to male (SAN plastic with silicone diaphragm, pack of 10, part no. UX-30505-92, Cole-Parmer, Vernon Hills, IL). Cracking pressure = 0.174psi (part no. 11582, Qosina, Ronkonkoma, NY). Cracking pressure = 0.217psi. Used as a key part of the food pump: As an alternative, a duckbill check valve can be used (part no. 80065, Qosina, Ronkonkoma, NY). Cracking pressure = 0.433 psi. These provide a good seal to prevent backflow.
Used as part of the food pump to prevent liquid food from entering the purge airline. This part is connected to the Y connector (one can use two of these check valves in series, if desired, for extra security, though this does not appear to be necessary).

Feeding System Micro-controller: (to regulate automated feeding), Figure 5C:

To build this microcontroller unit please see instructions, fritzing diagram (“Feeding controller2.fzz”) and Arduino program sketch (“aquarium_feeding_controller9IRFinal copyl.int” program file). See **Additional Files 4-5** and **Figure 4C**.

3 Wire Capacitive Proximity Sensor Switch (4cm dia.; part no. 1 x Ljc30a3-h-z/bx Dc, Uxcell, https://www.amazon.com/dp/B0147XIWE6
This part has its own three conductor cable. Used to detect food in the reservoir. This part is held in position using the bracket described in **Additional File 3F-H**.

1 x USB + Serial Backpack Kit with 16x2 RGB backlight positive LCD - Black on RGB (Adafruit product no: 782. https://www.adafruit.com/product/782)
Used to display the time, date and status of the feeding cycles.

1 x Plastic bezel for LCD display (3D printed decorative plate to cover exposed edges of the LCD display and opening on the microcontroller housing. See “LCD_Bezel_98.stl” see **Additional File 6**).

1 x 9 VDC 1000mA switching power supply. This wall transformer has a 2.1mm dia. center positive male power plug. (Adafruit product no. 63, https://www.adafruit.com/product/63).
Used to provide power to the microcontroller unit.

1 x 19mm dia. RGB LED illuminated On-Off push button switch. (Adafruit product no. 3426, Adafruit. https://www.adafruit.com/product/3426).
Used to control power to the microcontroller unit and to display feeding system status via the built in LEDs.

1 x Arduino Uno microcontroller (SMD version, https://www.amazon.com/dp/B007R9TUJE/).
Used to regulate the feeding cycles and communicate with the Walchem 900 controller.

1 x Arduino Ethernet Shield (SunFounder Ethernet Shield W5100 for Arduino UNO R3 Mega product no. 2560 1280 A057, https://www.amazon.com/dp/B00HG82V1A). Used for an internet connection to relay various faults to the users. Note that there are different versions of the Wiznet ethernet chip. The Arduino program is designed to work with the W5100 chip, which is a slightly older chip. Some other chips are not compatible with the libraries used for compiling the Arduino program, and would require a slightly different script

1 x PCF8523 Real Time Clock (Adafruit product no. 3295, https://www.adafruit.com/product/3295).
Used as an internal clock to time the feeding cycles.

1 x 3 volt CR1220 Battery for Real Time Clock (RTC). (Adafruit product no. 380, https://www.adafruit.com/product/380)
Used to provide power to the real time clock. This should last for 4-5 years.

1 x small solderable copper plated perf board 1-7/8" x 2'1/4' (Circuit Specialists, Tempe, AZ, part no. 64-8932).
Used to mount various electronic components, the optocouplers and terminal blocks)

2x 10K resistors, ¼ watt, various suppliers.

1 x 2.2K resistor, ¼ watt, various suppliers.

5x 1K resistors, ¼ watt, various suppliers.

2 x MCT2E optocouplers (part no. MCT2E-ND, Digikey, https://www.digikey.com/products/en?keywords=MCT2E-ND).
Used for switching and electrical isolation of various parts.

2 x 6 pin DIP socket (part no. 571-1-2199298-1, Mouser Electronics, https://www.mouser.com/ProductDetail/TE-Connectivity/1-2199298-1?qs=fK8dlpkaUMsQ%252BTzExsKLsw%3D%3D)
Used for mounting the optocouplers.

4 x 2-pin terminal blocks (Adafruit product no. ID 724, https://www.adafruit.com/product/724).
Used for ease in wiring internal connections to the various data cables.

6 x nuts and machine screws of 4-40 size, 1/2" long, various suppliers.
Used for mounting the Arduino Uno and perf board.

4 x 2-56 machine screws, 1/4" long, various suppliers.
Used to secure the LCD.

6 Plastic standoffs, for 4-40 machine screws, 3-4mm long, various suppliers.
Used to mount the Arduino Uno and perf board.

2 small plastic standoffs for use with the 2-56 machine screws, 2mm long, various suppliers.
Used to mount the LCD.

24 gage insulated hook up wire, various suppliers.
Used for various electrical connections.

1 x Project box, 5.3” x 3” x 1.9” (part no. MB-113, All Electronics, Van Nuys, CA, https://www.allelectronics.com/item/mb-113/abs-project-box-5.3-x-3-x-1.9/1.html).
Used to house the relay module and outlets. Note that various openings will need to be machined to permit mounting of various components.

Used to house parts for the microcontroller. Note that various openings will need to be machined to permit mounting of various components.

5 x Water proof cable glands for cables, PG7 size. Various suppliers, (https://www.amazon.com/Connectors-Plastic-Protectors-Waterproof-Adjustable/dp/B07RHJM435/ref=sr_1_9?keywords=cable+glands&qid=1572968857&sr=8-9)
Used to secure the cables and prevent water incursion into the microcontroller project box.

4 conductor cable (18 feet), various suppliers
Used to connect the microcontroller to the stepper motor controller and Walchem 900 controller.

DB9 connector (9 pin male), various suppliers
Used to connect the microcontroller to the stepper motor controller.

Heat shrink tubing, various suppliers
Used to insulate various connections.

Remote relay outlet box to control power for the air pump and the stir plate, (Figure 5C):

4 conductor cable (6 feet), various suppliers
Used to connect the relay box to the microcontroller.

1 x Three prong grounded 120 volt dual power outlet, 8 feet long, various suppliers
Used to provide 120VAC power to the switched outlets for the air pump and stir plate. Note that the common hot wire connection between the two outlets has to be cut, as they are separately controlled by the relay module.

1 x Relay module (part no. MCIGICM 2 Channel DC 5V Relay Module for Arduino UNO R3 DSP ARM PIC AVR STM32 Raspberry Pi with Optocoupler Low Level Trigger Expansion Board, https://www.amazon.com/dp/B072BY3KJF).
Used to regulate power to the two outlets.

2 x Waterproof cable glands for cables to prevent water incursion. Need 1 x PG7 and 1 x PG9 sizes, various suppliers (see above).
Used to secure the cables and prevent water incursion into the outlet box.

1 x Project box, 5.3” x 3” x 1.9” (part no. MB-113, All Electronics, Van Nuys, CA, https://www.allelectronics.com/item/mb-113/abs-project-box-5.3-x-3-x-1.9/1.html).
Used to house the relay module and outlets. Note that various openings will need to be machined to permit mounting of various components.

1/2" self-adhesive Kwik Klips, adjustable cable clips (Used for managing electrical cords on the feeding and aquarium systems. (GKK0-1550 Kwik Klips, https://www.amazon.com/dp/B00002NAOE).
Used for external cable management around the system.

Mounting bracket for feeding system microcontroller unit, includes two 3D printed parts: (see Articulated arm base plate .stl files included in, Additional Files 7-8, and available on Henry Lab web site, www.life.illinois.edu/henry). Note that other arrangements can also be made for mounting the microcontroller. Figure 5C:

1 x Articulated Arm, all metal construction, Camvate 7” (147mm) articulated arm V1 black and red, with ¼-20 threads. (Camvate, https://www.amazon.com/dp/B017OTDZTM). **Figure 5C**.
Used with the two 3D printed parts, detailed below, to position the microcontroller, see **Additional Files 7-8**.

1 x Plastic circular, beveled plate (has center tapped with ¼-20 hole for articulated arm) to be affixed to the control box by heavy duty double stick foam tape. (see “Articulated arm base plate v10.stl” file provided).

1 x Semi-circular mounting plate (has center taped ¼-20 hole for articulated arm). Two counter-bored holes accept 10-32 cap screws for mounting this plate on the top of the beverage cooler using the unused door hinge threaded holes on the top of the mini refrigerator. Door is reversible so either left or right side can be used, but be aware of the arrangement of cables to best mount the arm. (See “Articulated arm base plate v10.stl” file provided).

External feed line sensor (Monitors food delivery, see documents provided in a separate file), Figure 5F:

1x Teflon feeding line clamp consisting of two parts, base and top (See “tubing holder v2.stl” files provided). See **Additional Files 9-10**.
Used to mount the IR LED and sensor.

1 x 5mm IR LED (Adafruit product ID 387, https://www.adafruit.com/product/387).
Used as a pulsed source of IR light.
This part is mounted (glued into) to the base of the food line clamp.

IR 38Mhz detector, TSOP4838 (Jameco part no. 2229351 (or TSOP1839), Jameco Electronics. Belmont, CA https://www.jameco.com/shop/ProductDisplay?catalogId=10001&langId=-1&storeId=10001&productId=2229351)

This part is mounted (glued into and secured with a screw-on retaining strap) to the base of the food line clamp.

Retaining straps (See “retaining strap v3.stl” file provided) **See Additional Files 11-12**.

2 x Water proof cable glands for cables to prevent water incursion. PG7 size, various suppliers. See above.
Used to secure the cables and prevent water incursion into the box.

4 conductor cable (6 feet), various suppliers
Used to connect the remote sensor to the microcontroller.

1 x Small water-proof box (BUD Industries, Inc., part number PN-1320)
Used to protect the components of the IR sensor.

<u>Peristaltic Food Pump Stepper Motor Controller: (used to regulate food delivery), Figure 5C:</u>

To build this microcontroller unit please see instructions, fritzing diagram (“peristaltic_pump_controller.fzz”) and Arduino program sketch (“stepper_motor_controller.int” program file). See **Additional Files 13-14** and **Figure 5C**.

1 x Arduino Uno microcontroller (SMD version, https://www.amazon.com/dp/B007R9TUJE/).
Used to regulate food delivery and communicate with the Walchem 900 controller.

1 x USB + Serial Backpack Kit with 16x2 RGB backlight positive LCD - Black on RGB (Adafruit product no: 782. https://www.adafruit.com/product/782)
Used to display the settings (i.e., speed, and number of steps) for the stepper motor.

1 x Project box, 5.3” x 3” x 1.9” (part no. MB-113, All Electronics, Van Nuys, CA, https://www.allelectronics.com/item/mb-113/abs-project-box-5.3-x-3-x-1.9/1.html).
Used to house the relay module and outlets. Note that various openings will need to be machined to permit mounting of various components.

1x 10K resistor, ¼ watt, various suppliers.

1 x Analog 2-axis joystick with select button and breakout board. (Adafruit product no. 512, https://www.adafruit.com/product/512).
Used to input settings for speed and number of steps.

1 x small solderable copper plated perf board 1-7/8” x 2’1/4’ (Circuit Specialists, Tempe, AZ, part no. 64-8932).
Used to mount electronic components, and to make various wire connections.

1 x Plastic bezel for LCD display (3D printed decorative plate to cover exposed edges of the LCD display and opening on the microcontroller housing. See "LCD_Bezel_98.stl" see **Additional File 6**).

4 conductor cable (8 feet), various suppliers
Used to connect the stepper motor controller's microcontroller to the Feeding system's microcontroller and to the stepper motor in the peristaltic food pump, which is located inside the mini-refrigerator.

1 x DB9 connector (9 pin female), various suppliers
Used to connect the microcontroller to the feeding controller.

1 x 9 VDC 1000mA switching power supply. This wall transformer has a 2.1mm dia. center positive male power plug. (Adafruit product no. 63, https://www.adafruit.com/product/63).
Used to provide power to the microcontroller unit.

2 x Waterproof cable glands for cables to prevent water incursion. Need 2 x PG7 size, various suppliers (see above).
Used to secure the cables and prevent water incursion into the project box.

6 x nuts and machine screws of 4-40 size, 1/2" long, various suppliers.
Used for mounting the Arduino Uno and perf board.

4 x 2-56 machine screws, 1/4" long, various suppliers.
Used to secure the LCD.

6 x Plastic standoffs, for 4-40 machine screws, 3-4mm long, various suppliers.
Used to mount the Arduino Uno and perf board.

2 x small plastic standoffs for use with the 2-56 machine screws, 2mm long, various suppliers.
Used to mount the LCD.

24 gage insulated hook up wire, various suppliers.
Used for various electrical connections.

1 x 16mm dia. Green LED illuminated On-Off push button switch. (Adafruit product no. 482, Adafruit. https://www.adafruit.com/product/482).
Used to control power to the microcontroller unit.

<u>Protein Skimmer (Used to remove uneaten food and dissolved organic material), Figure 3C-F:</u>

1 x Tunze Comline 9004 DC DOC skimmer (DC motor version with DC motor controller) (Tunze, Penzberg, Germany). **Figure 3C**.

1 x Plastic bulkhead connector single port, for 3/8" OD silicon tubing, with NPT threaded connector (5694T161, McMaster Carr, Elmhurst, IL) and **½"** ID 3.32 O-ring and Teflon tape. Corresponding ¼" NPT threaded nut and **½"** ID 3.32 O-ring and Teflon tape to seal connection. Hole for this fitting is drilled into side of Tunze collecting cup towards the bottom. Inner shoulder located in the central passage way of this of fitting is drilled out to allow for complete thru passage of the silicone tubing.
Used as a continuous drain connection for the collecting cup silicone tubing.

1 x Plastic bulkhead connector dual ports, for 3/8" OD silicon tubing (To be installed in the side wall of the sump (**Figure 3F**). 5694T17, McMaster Carr, Elmhurst, IL). Inner shoulder located in the central passage way of this fitting is drilled out to allow for complete through passage of the silicone tubing.
Used to allow for passage of the silicone skimmer drain line through the wall of the sump.

Silicone tubing, Silastic Laboratory Tubing, Four feet long, 0.188" ID x 0.375" OD. (part no. 515-014, Dow Corning, Midland, MI).
Used as a drain tube for the collecting cup.

Custom motor control interface (regulates power to the protein skimmer via the Walchem 900 controller), Figure 5G:
Fritzing diagram ("DC motor controller interface2.fzz") for assembling the circuit, and program for the ATtiny85 chip ("ATtiny_motor_controller copy.ino"). See files provided in **Additional Files 15-16**.
Used to automatically turn off protein skimmer during feeding.

Fine insulated wire, size 30, solid strand, any 30 gauge wire wrapping wire should work (https://www.amazon.com/dp/B07WRTWWZW)

1x ATtiny85 operating at 1MHz (Sparkfun, COM-09378, https://www.sparkfun.com/products/9378). Note this is programmed using Arduino IDE and SparkFun Tiny AVR Programmer (see below).

Tiny AVR Programmer (part no. PGM-11801, https://www.sparkfun.com/products/11801). Required to program the ATtiny85, above.

3x 10K resistors, ¼ watt, various suppliers

3x 1K resistors, ¼ watt, various suppliers

1 x Zener diode *removed from DC motor control board (see **Figure 5H,** details published in Henry et al., 2019).

1 x MCT2E optocouplers (part no. MCT2E-ND, Digikey, part no. MCT2E-ND, Digikey, https://www.digikey.com/products/en?keywords=MCT2E-ND).
Used for switching and electrical isolation of various parts.

1 x 6 pin DIP socket (part no. 571-1-2199298-1, Mouser Electronics, https://www.mouser.com/ProductDetail/TE-Connectivity/1-2199298-1?qs=fK8dlpkaUMsQ%252BTzExsKLsw%3D%3D)
Used for mounting the optocoupler.

1 x 8 pin DIP socket.
Used for mounting the ATtiny85 chip.

1 x 10µf capacitor.

1 x small solderable copper plated perf board 1-7/8" x 2'1/4' (Circuit Specialists, Tempe, AZ, part no. 64-8932). Trim to fit inside housing.
Used to mount various electronic components, the optocouplers and terminal blocks..

1x custom cut insulating thin plastic sheet (same size as perf board)
Used as insulation.

4 conductor cable (8 feet), various suppliers
Used for connecting the interface to the Walchem 900 controller.

24 gauge insulated hook up wire

2 x Rubber grommets (5/16" OD)
Used to secure the cable, provide strain relief and prevent water incursion into the housing.

2 x small zip ties, various suppliers
Used to secure the cables.

3D printed housing and cover plate to enclose the circuit (See 3D printer stl file: "control box v7.stl" provided). See **Additional Files 17-18**.
Note that once installed the lid can be secured using a small amount of silicone cement.

Additional components needed for the rack system (e.g., sea salts, media for the various filters, and components needed to culture small animals, and to reduce splashing, evaporation and to regulate sea water level): Note, most of the standard components that make up the rack system provided by Iwaki Aquatics, are not listed here.

Instant Ocean Reef Crystals artificial sea salt (Spectrum Brands, Inc. Blacksburg, VA, UPC code 51378 01800).
Used as the artificial sea salts to prepare the sea water, following the manufacturer's instructions.
Seed Bacteria (Aquavitro, Seachem, Madison, GA) or Dr. Tim's One and Only Live Nitrifying Bacteria (Dr .Tim's Aquatics, LTD, Moorpark, CA).
Used initially to seed the biofilter.

"Marine Buffer" (Seachem, Madison, GA).
Used to raise the pH of the sea water, as needed.

Filters and filter media

Two Acurel Filter lifeguard media bags, 9" x 13" (prod. ID 42982 08033, Acurel, Cranbury, NJ). Placed inside the right-hand sump compartment and used to contain the crushed shell and coral, so that these course debris cannot escape and enter the water pump and filters. These bags should be securely tied shut once filled.

Crushed coral (Geo-Marine Argonite formula 15lb bag. Product ID 0847900120, CaribSea, White City, FL).
Used as a source of calcium and as a buffer for the sea water. The substrate is placed inside two fine mesh bags, above, which go into the right sump compartment, so that the coarse debris cannot enter the water pump and filters.

Biological filter media consisting of a mix of 800 gm of Matrix (Seachem, Madison, GA) and 100 gm of de*Nitrate (Seachem, Madison, GA).
Used as a substrate to support aerobic, anaerobic and facultative bacteria growth.

1-2 lbs of activated carbon pellets (Kent Reef Carbon Pellets, Kent Marine, Franklin, WI).
Used to remove dissolved organic compounds from the sea water.

Polyhydroxyalkanoate (PHA) polymer pellets (Vertex Aquaristik Pro-Bio Pellets, Huntington Beach, CA).
Used to support nitrifying bacteria, should be replenished, as needed.

Replenish, (Seachem, Madison, GA).
Used to restore general hardness to RODI.

Automatic top off unit to add sea water, Figure 4H-I:

System automatic top off unit (ATK unit, Neptune Systems, Morgan Hill, CA).
Used to automatically add sea water. **Figure 4H**.

Optical sensor with magnetic mount (OS-1-M, Neptune Systems, Morgan Hill, CA).
Used to monitor the level of sea water inside the reservoir. **Figure 4I.**

Small nylon basket ("The People's Brew Basket", The Republic of Tea, Novato, CA).
Used as a filter to prevent debris and ppt from entering the small ATK PMUP water pump. **Figure 4I.**

Clean 5 gallon plastic paint bucket with lid, various suppliers.
Used as the reservoir for the sea water. Note that small holes need to be drilled through the lid to allow for the passage of the power cord, optical sensor cable and plastic tubing. **Figure 4H.**

Parts to help limit evaporation and water loss:

1/2” Off-white plastic snap in plugs for panels with tapered prongs (need 200) off-white color. Used to cover the 1/2" holes in tank lids. (Part no. 9688K251; 100 pcs each, 9688K25162PP050BG14, McMaster Carr, Elmhurst, IL.). **Figure 1G**.

Custom machined 1/4” thick PVC covers for the drain troughs (**See Additional File 3B-C). Figure 1H-I.**

Polycarbonate “Origami” cover for the large sump drain opening (**See Additional File 3D, Figure 3B.**

<u>Culture tubes for animals:</u>

Ultra-stiff mesh plastic canvas (for needle point). (#7 mesh = openings per inch). Used to create a curved barrier to keep culture tubes submerged inside the small tanks. Darice Canvas 3pc. Designer Ultra stiff Canvas 12”x18” (product ID 826709455). Darice, Inc. Strongsville, OH. Each piece can be cut with scissors to make two barriers (20cm x 22.5cm). **Figure 1D, J**.

Screened culture tubes (See Henry et al., 2017). Small culture chambers to contain animals are constructed from either 50ml or 15ml screw top polypropylene tubes (Corning # 430897 (50ml) and Nunc # 339651 (15ml) Corning Inc., Corning, NY; and Nalgene Nunc, Rochester, NY, respectively), with open ends covered by fiberglass screens (see Henry et al., 2017). **Figure 1D**.

<u>External Chiller, Figure 4K:</u>

1 x Artica Titanium Chiller, part no. DBA-075 (JBJ Aquarium, Inglewood, CA). Used to lower sea water temperature. **Figure 4K**.

2 x Custom machined PVC adapters for the water chiller (See documents provided in a separate file). (**See Additional File 3E).**
Used to adapt chiller water connections to 3/8” plastic tubing. **Figure 4L**.

3/8” OD tubing by ¼ NPT male threaded right angle polypropylene compression fittings (part no. 9087K23, McMaster Carr, Elmhurst, IL). Needed to hook up the plastic tubing. **Figure 4L.**

2 x Sets of clamps/bolts/cables to secure the rack.
Used to secure the top of the rack and prevent it from tipping over. Exact parts depend on where the rack is located and what supports are available to attach these parts. **Figure 1B**.

4 x Custom machined ½ inch thick, 4” x 4” square aluminum foot pads. Each has a central 1/8” deep by 1’ -3/8” dia. recess for the leveling feet.
Used to distribute the weight of the rack system. **Figure 1C**.

<u>Useful/Essential tools (needed to service the system): Additional File 1: Figure S1.</u>

1 x 9” Channellock filter and PVC slip joint pliers (Channellock, Inc., Meadville, PA).

1 x Pentex SW 1A filter wrench (Pentair, Minneapolis, MN).
Used for small bio-reactor filter bowl.

1 x Pentex SW 3 filter wrench (Pentair, Minneapolis, MN).
Used for large filter bowls.

1 x Lab glass alcohol thermometer -20°C to 120°C, or certified waterproof digital thermometer.

1 x Apera Instruments SX610 pH pen tester (Apera Instruments, Columbus, OH).

1 x Hanna Instruments Salinity Tester (model no. HI98319 Marine Line Salinity Tester, Hanna Instruments, Inc. Woonsocket, RI).

1 x Test kit to monitor ammonia, nitrites and nitrates. API Marine (Mars Fishcare North America, Chalfont, PA). Note that the API "Saltwater Master Test Kit" includes solutions to test for all three, in addition to pH. In addition, the "Reef Master Test Kit": will measure calcium, carbonate hardness, phosphates and nitrates.

1 x Extech IR thermometer, IR250. Optional, used to measure temperature of the main water pump housing to assess its operating condition (Extech, Nashua, NH).

Long handled (extendable) ice scrapper, various suppliers.
Used to remove stubborn salt deposits.

Spare/replacement parts:

Backup Iwaki seal-less magnetic drive pump with a polypropylene centrifugal impeller (model no. MD-70RLZT, Iwaki Aquatics, Holliston, MA).

Walchem WEL series pH electrode (model no. 191653-20, Iwaki Aquatics, Holliston, MA).

Walchem contacting conductivity sensor (model no. 103906-10, Iwaki Aquatics, Holliston, MA).

Conductivity calibration solution with a conductivity of 1000µS/cm at 25°C (Part no. YSI 3167, 060907, YSI, Inc., Yellow Springs, OH).

pH calibration solutions (Walchem part no. 51370, pH 7.00 and Walchem part no. 51371, pH 10.00, Iwaki Aquatics, Holliston, MA).

Coarse filter pads (AQ1139, 12" X 12" size. Iwaki Aquatics, Inc., Holliston, MA)

10" x 4.5" 50 micron pleated polyester filter cartridges, Keystone, GPE-50-10 (equivalent part no. SPC-45-1050, Water Filters Fast, Hot Springs, AR; or part no. AQ1091, Iwaki Aquatics, Inc., Holliston, MA).

Activated carbon pellets (Kent Reef Carbon Pellets, Kent Marine, Franklin, WI).

Polyhydroxyalkanoate (PHA) polymer pellets (Vertex Aquaristik Pro-Bio Pellets. Huntington Beach, CA).

Extra 10 liter Pentair polycarbonate tanks (part no. PC90, Pentair Aquatic Ecosystems, Minneapolis, MN).

Extra 3 liter Pentair polycarbonate tanks (part no. PCT3-D, Pentair Aquatic Ecosystems, Minneapolis, MN).

400µm Screened baffles, 10 liter (part no. 10-4-A, Pentair Aquatic Ecosystems, Minneapolis, MN).

Screened baffles, 3 liter (part no. 3-4-A, Pentair, Minneapolis, MN).

Injection molded lids, 10L (part no. LID90I-4, Pentair Aquatic Ecosystems, Minneapolis, MN).

Injection molded lids, 3L (part no. LID2751-5, Pentair Aquatic Ecosystems, Minneapolis, MN).

Luer lock Y connectors, each with two female Luer lock fill ports and single male Luer lock connector with spin lock, ABS, PP plastic (part no. 84049, Qosina, Ronkonkoma, NY).

Luer lock one way check valves, female to male, SAN plastic with silicone diaphragm, female to male, pack of 10 (part no. UX-30505-92, Cole-Parmer, Vernon Hills, IL). Cracking pressure = 0.174psi, or (part no. 11582, Qosina, Ronkonkoma, NY). Cracking pressure = 0.217psi

Luer lock tubing, 48" long with male and female ends (part no. 900 SPI 3/16 OD, 30526-28 Cole Parmer, Vernon Hills, IL).

Barbed Luer lock connector, male (part no. 11527, Qosina, Ronkonkoma, NY).

HPLC waste line adapter (part no. HPLC-LL-1/8-KIT, CPLabSafety, San Francisco, CA).

PTFE Teflon tubing (feeding line) (6-8 feet long, as needed) 1/16" ID, 1/8" OD. (part no. 53XL53, Grainger Industrial Supply, Alsip, IL)
Used as part of the food pump to deliver food to the sump compartment. Length should be kept to a minimum.

Classic quartz sleeve 57 watt 19" long (part no. A10057, Aqua Ultraviolet, Temecula, CA).

UV lamp 57 watt Aqua Ultraviolet lamp (part no. A20057, Aqua Ultraviolet, Temecula, CA).

Acknowledgements:
This work was supported by an NSF EDGE grant (1827533) to J.Q.H. (J.J.H).

References:

Henry JQ, Lesoway MP, and Perry KJ, (2020). An Automated Aquatic Rack System for Rearing Marine Invertebrates. (BMC Biology, In Press).

Henry JQ, Lesoway MP, Perry KJ, Osborne CC, Shankland M, and Lyons DC. (2017). Beyond the sea: *Crepidula atrasolea* as a spiralian model system. Int. J. Dev. Biol. 61: 479-493.
