# Supplementary figures and images for: An automated aquatic rack system for rearing marine invertebrates
Source: BMC Biol. 2020 May 4;18:46. doi: 10.1186/s12915-020-00772-w (PMC7199361; doi:10.1186/s12915-020-00772-w)

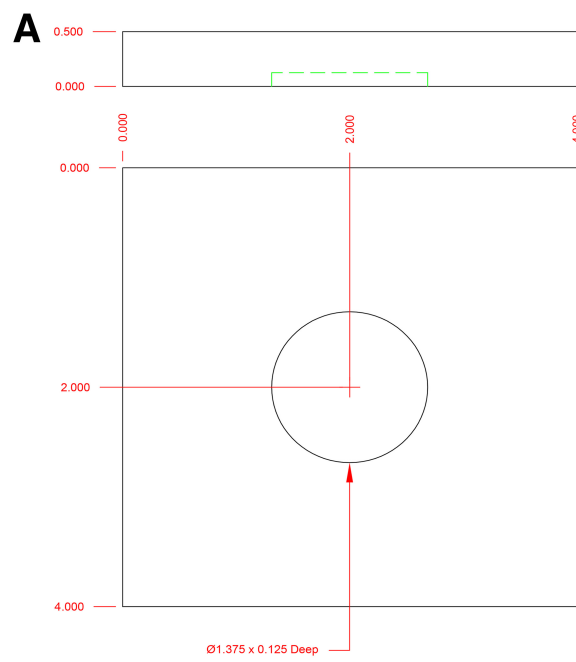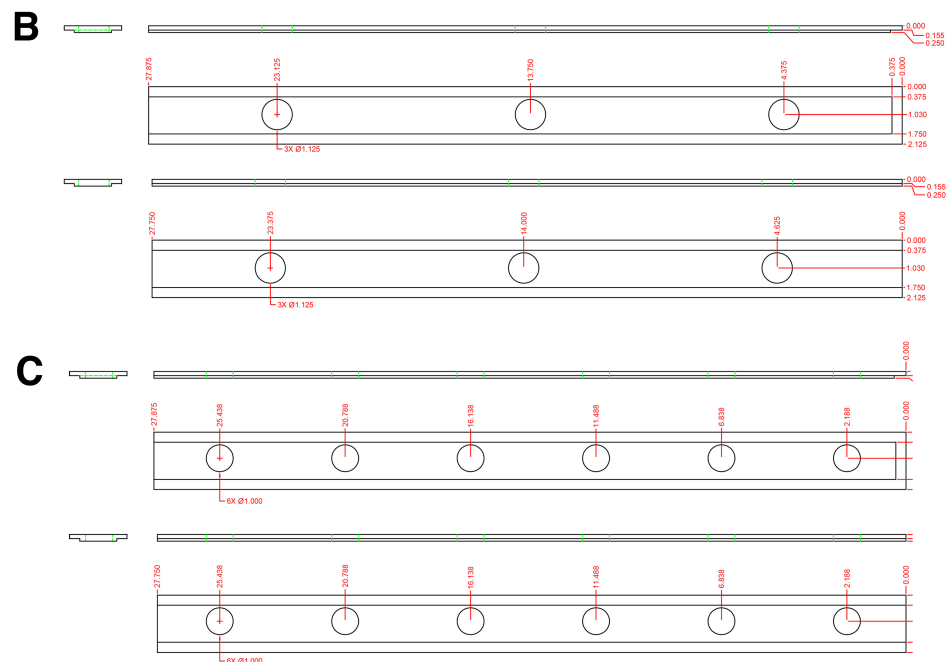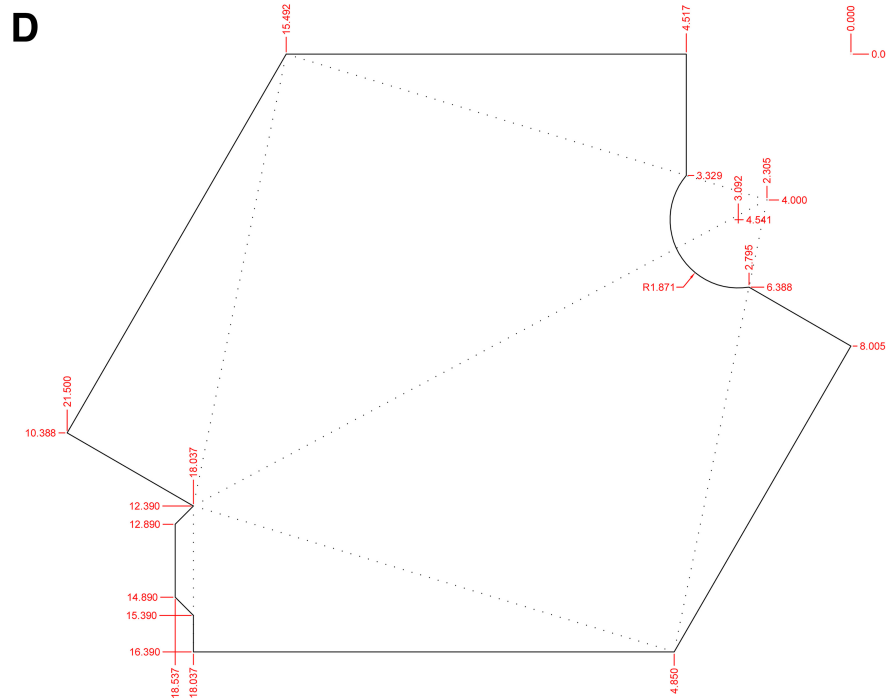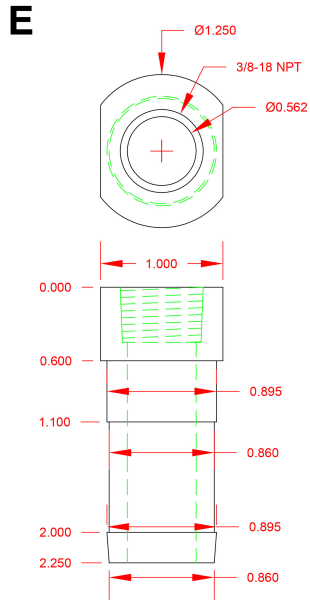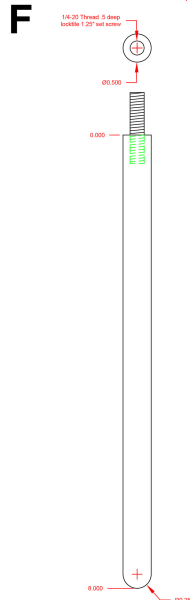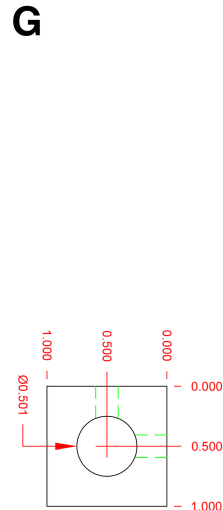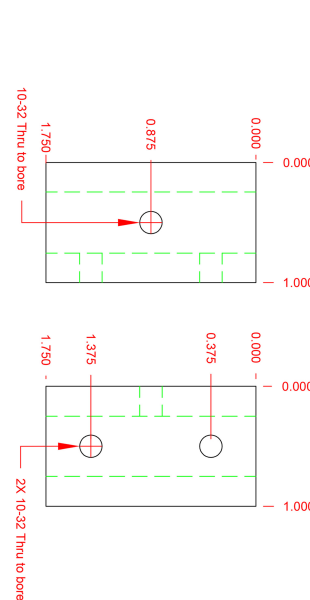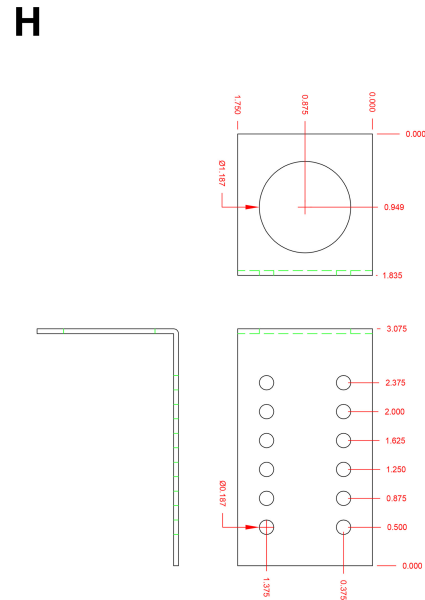

Supplement: Supplementary file 3 — Additional file 3: Construction diagrams for fabricating various components used in the aquatic system. Dimensions are indicated in red and hidden holes and recesses are showing in green for various projections. A. The aluminum foot pads to help distribute the weight of the system on the floor. Four of these pads are required. The rounded recesses receive the stainless steel adjustable feet. B-C. The PVC covers for the drain troughs to reduce evaporation and splashing are machined from “¼”: thick grey plastic PVC. Two styles accommodate B) the drain trough for the six larger 10 liter tanks located on the bottom shelf, and C) the drain troughs serving twelve 3 liter tanks situated on each of the five upper shelves. For ease of installation, each of these covers consists of two pieces, as shown. D. The polycarbonate “Origami” cover for the right sump compartment is cut form a single sheet of “1/8” thick clear polycarbonate. Dashed lines indicate folds. All dimensions are indicated relative to the zero (0,0) reference point shown. E. Custom machined plumbing connections to adapt the Artica chiller to “3/8” right angle polypropylene compression fittings for the tubing that serves the sensor side loop. Two of these fitting are needed for influent and effluent sea water. These parts are used with the rubber seals provided by Artica for the original fittings. F-H). The adjustable aluminum metal clamp system used for the capacitance food reservoir, level sensor consists of three parts: F) the threaded post, G) the adjustable clamping block and H) the adjustable L-shaped bracket that holds the sensor. Machine screws (not shown) are needed to assemble these last three parts [41]. [file 12915_2020_772_MOESM3_ESM.pdf]
